# Child Centred Approach to Climate Change and Health Adaptation through Schools in Bangladesh: A Cluster Randomised Intervention Trial

**DOI:** 10.1371/journal.pone.0134993

**Published:** 2015-08-07

**Authors:** Md Iqbal Kabir, Md Bayzidur Rahman, Wayne Smith, Mirza Afreen Fatima Lusha, Abul Hasnat Milton

**Affiliations:** 1 Department of Community Medicine and Clinical Epidemiology, Centre for Clinical Epidemiology and Biostatistics, School of Medicine and Public Health, Faculty of Health and Medicine, The University of Newcastle, Newcastle, New South Wales, Australia; 2 Department of Epidemiology, National Institute of Preventive and Social Medicine, Mohakhali, Dhaka, Bangladesh; 3 Department of Public Health and Community Medicine, Faculty of Medicine, University of New South Wales, Sydney, New South Wales, Australia; 4 Climate Change and Health Promotion Unit, Ministry of Health and Family Welfare, Dhaka, Bangladesh; University of New South Wales, AUSTRALIA

## Abstract

**Background:**

Bangladesh is one of the most vulnerable countries to climate change. People are getting educated at different levels on how to deal with potential impacts. One such educational mode was the preparation of a school manual, for high school students on climate change and health protection endorsed by the National Curriculum and Textbook Board, which is based on a 2008 World Health Organization manual. The objective of this study was to test the effectiveness of the manual in increasing the knowledge level of the school children about climate change and health adaptation.

**Methods:**

This cluster randomized intervention trial involved 60 schools throughout Bangladesh, with 3293 secondary school students participating. School upazilas (sub-districts) were randomised into intervention and control groups, and two schools from each upazila were randomly selected. All year seven students from both groups of schools sat for a pre-test of 30 short questions of binary response. A total of 1515 students from 30 intervention schools received the intervention through classroom training based on the school manual and 1778 students of the 30 control schools did not get the manual but a leaflet on climate change and health issues. Six months later, a post-intervention test of the same questionnaire used in the pre-test was performed at both intervention and control schools. The pre and post test scores were analysed along with the demographic data by using random effects model.

**Results:**

None of the various school level and student level variables were significantly different between the control and intervention group. However, the intervention group had a 17.42% (95% CI: 14.45 to 20.38, P = <0.001) higher score in the post-test after adjusting for pre-test score and other covariates in a multi-level linear regression model.

**Conclusions:**

These results suggest that school-based intervention for climate change and health adaptation is effective for increasing the knowledge level of school children on this topic.

## Introduction

In the development of climate science and policy making the adverse effect of climate change on human health has been recognized relatively late [1,2]. The recent Fifth Assessment Report (AR5) of the Intergovernmental Panel on Climate Change (IPCC) reinforces the need for societies to take adaptive actions to protect human health from the adverse consequences of climate change [3]. Climate sensitive health determinants and outcomes increase threat to children’s health and family livelihoods in Least Developed Countries [4, 5]. To protect the health of affected inhabitants, highly strategic interventions for adaptation will be needed over the next 20–30 years [6, 7].

Since 2007, Bangladesh ranked highest on the risk index of climate victims prepared by the IPCC [8]. The lives and livelihood of 36 million people in the southern coastal regions are directly affected by climate change [9]. Although it is obvious that children are most vulnerable subpopulation because of their greater exposure and sensitivity to health outcomes, there has been a lack of investigation into the health impacts on children associated with climate change [10, 11]. The World Health Organization (WHO) developed internationally comparable children’s environmental health indicators (CEHIs) to allow consistent tracking of the state of children’s (0–14 years) environmental health. Review of recent research on climate change and child health shows that child-centred adaptation has been relatively limited [12–14]. The concept of intergenerational equity, the actions we take today to protect the future generations, requires prioritisation to achieve sustainable development [15–17].

With children being innately vulnerable, it is important to direct prevention efforts to reduce their exposure and susceptibility to adverse health effects from climate [10]. Schools must hold the capacity for curriculum development to enhance adaptability to climate change so that children are familiar with the concept and practices associated with the advocated changes. Several global examples reveal that the key to building resilience and adaptive capabilities in children lies with preparing them at the school levels [18].

In a test implementation of a school-oriented drug prevention programme “study without drugs” involving of sixty second grade students at a junior high school in Paramaribo, Surinam post–testing showed that participating students obtained an increased knowledge of drugs, and that their skills to resist drugs were enhanced [19]. Previously, in a cluster randomized trial of sex education in seven high schools in Belize, Central America a greater improvement in knowledge was observed in the intervention group than in the control group [20]. The ‘Michigan Model Nutrition Curriculum’ was evaluated among 576 grade seven children. The study showed enhanced nutritional knowledge in the intervention group; students in the intervention group were more likely to eat fruits and vegetables and less likely to eat junk food than the control group [21]. A school-level cluster randomized control trial on adolescent cigarette smoking was conducted among 7^th^ and 8^th^ grade students in four junior high schools in southern China. The mean knowledge scores from baseline increased more in the intervention group than in the control group, although there was little change in the attitude scores [22]. Similar to the Save the Children’s experience the best success was achieved when children participated actively in risk reduction strategies rather than being passive recipients of services [23].

In 2008 the South East Asia Regional Office of the World Health Organization (WHO) developed a manual for school children to promote a child-centred approach to achieve a lifestyle adaptation for reducing the health vulnerability. Based on this WHO manual, the Climate Change and Health Promotion Unit (CCHPU) of the Ministry of Health and Family Welfare, Bangladesh developed a school manual on ‘Climate Change and Health Protection’ that was printed in the local language Bangla to make it easy to understand for teachers, students, and their families [24]. A range of stakeholders from government and civil society, experts on climate change and on the development of educational manuals, public health specialists and communication experts contributed to preparing the manual. The National Curriculum and Textbook Board under the Ministry of Education endorsed the manual and distributed it to high school students from classes six to ten for supplementary reading. The manual has seven chapters: chapter one deals with issues of climate and how the climate is changing. Climate change and its effect on health, climate sensitive diseases and other environmental health issues are discussed in chapter two. In chapter three, the risk management of health hazards due to Climate Change is addressed. Conservation of the environment and natural resources of Bangladesh is the content of chapter four. Chapter five comprises three case studies that illustrate climate change and health protection issues. Chapter six describes how to reduce environmental pollution (air, soil, water, river, sound) and how to keep a healthy life style. Chapter seven outline the plan for three days of practical lessons on climate change and health adaptation.

It was anticipated that this manual will help to increase the knowledge level of high school students on health adaptation to climate change. No study has been conducted so far to examine the effectiveness of this manual in increasing the knowledge of the school children in climate change which would contribute to child centred adaptation. An approach is evidence based when its functional effectiveness has been proved through scientific research [25, 26]. Recently two systematic reviews were published on public health interventions to adapt to climate change [27, 28]. A recent Evidence-Based Public Health (EBPH) approach to Climate Change Adaptation (CCA) review revealed that developing appropriate intervention methods could build the evidence base in lower-resource settings such as Bangladesh [28].Other studies also emphasized the need for intervention and knowledge-translation research relevant to CCA and introduce evidence-based methods into public health practice [29–36].

Schools are used as emergency disaster shelters in coastal Bangladesh during extreme weather events such as cyclones and, flooding. According to a government report, flood-related diarrhoeal diseases have increased in recent years. In coastal areas, salinity intrusion is aggravating fresh water scarcity, leading to increased water borne diseases. By 2050, the eastern and southern parts of Bangladesh will be highly vulnerable to diarrhoeal incidence. Seasonal cholera outbreaks can become a regular phenomenon in future. Spatial distribution of vector- borne diseases will change and increase in now low risk areas. An increase in temperature and humidity, as well as high population density may cause more diseases and aggravate the total burden of diseases [37]. Providing school children with knowledge on climate change and health protection may be an important initial step for child-centred health adaptation to reduce the future burden of climate sensitive diseases.

In this novel cluster randomized intervention trial we evaluated the effectiveness of this ‘Climate Change and Health Protection’ manual in increasing the knowledge level of school children.

## Methods

### Recruitment and randomisation of schools

In Bangladesh, thirty upazilas (sub-district) of seven coastal districts distributed in five different regional divisions were selected as most vulnerable for climate change and extreme weather events. This trial was conducted among the secondary school participants of these widely distributed areas of Bangladesh from August 2012 to June 2013. Two secondary schools were randomly selected from each of the 30 upazilas. From each selected school, all the students studying in class seven (grade seven) were recruited. Class seven students were selected because they are early teens (13–14 years) in the second year for high school and could be followed for three to five consecutive years.

Among 30 upazilas, 15 were randomly allocated to the intervention group and 15 to the control group. We randomly allocated upazilas as opposed to schools in order to avoid contamination because all secondary schools in an upazila work under a single association to run their academic activities. Random selection of schools and random allocation of the upazilas were carried out by an independent statistician generating and sorting random numbers in a SAS (version 9.3) program. Out of the 60 schools, 30 received the intervention while the other 30 served as controls.

### Sample size calculation

To obtain 90% power with a two-sided 5% significance level for detecting a 25% improvement in the climate change related knowledge among the students of intervention school compared to the control school, assuming baseline knowledge of 15%, we required 65 students (total of 130) in each group. Assuming an intra-cluster correlation of 0.3 among school children with an average cluster size of 50 (m) the design effect (deff) is deff = 1 + (m-1) × ICC (Intra-cluster correlation) = 1 + (50–1) × 0.3 = 15.7. Thus, after taking into account the design effect we required 130*15.7 = 2041 students. In rural Bangladesh the expected compliance rate among the school children is approximately 70%. To account for non-compliance, we needed to recruit at least 2041/.70 = 2916 children. Therefore, on average, 50 students from class seven from 60 selected schools (total of 3000 approx.) were recruited in this intervention trial.

### Recruitment and training of study personnel

Before starting field activities, intensive week-long training was provided for the recruited field supervisors, facilitators and interviewers of the study. Three teachers (headmaster, class teacher of year seven and science teacher) from each of the 60 schools attended a three-day TOT (Training of the Trainers) session at divisional headquarters about the school manual. These teachers then acted as the facilitators and resource persons for the intervention at their schools. The year seven students were recruited through their class teachers, and enrolled with the written consent of the parents/guardian.

### Development and pre-testing of climate change knowledge questionnaire

We reviewed literature for questionnaires about climate change and health knowledge, attitude and behaviour, but we found very few that were suitable to evaluate specific school-based interventions. We also contacted experts in the field to check for availability of a valid tool to assess climate change knowledge. None of the available tools were specific for school children. Thus we developed a questionnaire using a combination of questions from the contents of the manual. This was used for a trial run among 100 year seven high school children in two rural high schools similar to the study schools. On the first day of the three-day training we arranged a pre-test of the questionnaire, then provided the training based on the manual. On the last day tested the students again, using the same questionnaire. The following day (day 4), we discussed the language and topics of the questions in an open session with the students and the teachers. Based on that discussion we edited the questionnaire. The final modified knowledge questionnaire comprised a set of 30 questions on various aspects of climate change and health issues and took around 20 minutes to complete. Twelve of these questions tested knowledge on the health effect of climate change, concerning: water-borne disease, vector- borne diseases, malnutrition, mental health, extreme weather events, school health curriculum, effect of ultraviolet rays, environmental pollution, climate sensitive vectors and health adaptation. Eight questions tested knowledge about climate and factors involved in its change: global warming, greenhouse gases, sea level rise and, water reserves. Six questions tested climate change adaptation and mitigation knowledge, four focused on natural conservation, solar energy and water resources. We also developed a questionnaire to collect descriptive statistics on each participant about their family and socio-economic condition, and a check list of school level characteristics to get an understanding about the facilities available in the schools of these vulnerable areas.

### Ethical approval and consent

Recruitment commenced on 1 August 2012 and final follow up was completed on 31 May 2013. The study protocol was approved by the Bangladesh Medical Research Council and by the Human Research Ethics Committee of the University of Newcastle, Australia (H2012-0163). At the beginning of the study, written informed consent was obtained from the headmasters of each school to confirm the school’s participation in the study. School teachers handed an information sheet describing the purpose of the study and individuals’ rights as study participants to the participants’ parents or care givers. Parents or care givers signed that after reading the information notes and students brought them back to the class teachers. Class teacher submitted the consent forms to the headmaster and the data collectors collected all the consent forms from the headmaster before the pre-test at the beginning of the study.

### Delivery of the intervention

All the students in groups performed a pre-test of 30 uniform short questions on climate change, health adaptation and mitigation issues. A total of 1515 students from 30 intervention schools received the intervention in the form of a three- day, formatted training programme presented in the classroom by the teachers trained on the use of the school manual. All of the intervention students received a printed manual written in the common local language (Bangla) to be kept with them for further reading and practice. The manual contains seven chapters that include essential knowledge about climate change and health issues, the ‘do’s and don’ts’ during extreme weather events, and adaptation activities. The science teacher used the topics in the manual as basis for a weekly 45-minutes classroom discussion over a six month period. A leaflet containing general message on climate change and health was distributed among the 1778 students of the 30 control schools after the pre-test, and students were asked to keep it for reading. Participants of the control group did not get the manual; they had only the classroom discussions by the science teacher. Six months later, students from both control and intervention schools were again tested on the pre-test questionnaire.

### Blinding

At the beginning the interviewers were blind about the intervention or control upazila. A unique code number was provided to each of the participants in both intervention and control group to use on their score sheet for the pre-test. The six digit code was comprised of the cluster code of the school, and the class roll number of the student. The data collectors handed over the coded questionnaire-answer-sheets to external evaluators for marking. The post-test was conducted with the same code numbers for each student by a different set of data collectors in both intervention and control schools. There was a considerable geographical distance between each of the randomly allocated upazilas, so all intervention schools were far away from the control schools. This procedure ensured an unbiased, blinded evaluation of the pre- and post-intervention scores of the participants. The investigators were not involved in the assessments. The evaluators (outcome assessors) and the statistician (M.B.R) were blinded to the group allocation until after the analysis was complete.

### Data collection

The trained recruited interviewers went to each school on a scheduled date and collected the basic information of each student with their household composition and socio-demographic variables (family size, type of family, type of household, education, occupation and age of family members, monthly total family income) in the classroom setting with the help of class teachers. Then the pre-test was taken in a separate coded question-answer sheet. The interviewers also collected data on each school’s characteristics using an observation checklist. Pre-test in all the intervention and control school were completed within two weeks-time period. The pre-test was evaluated by different assessors. Then baseline data was collated at individual and cluster group level, according to the coding. Investigators visited the schools for follow-up during the intervention period. Six month after the pre-test, on completion of the intervention, a post-test was conducted in each of the schools by different interviewers than those that applied the pre-test. The post-test was taken under the conditions as the pre-test. A group of outcome assessors marked the post-test answers and cross-checked data were double-entered. Correct answers scored +1 and incorrect ones scored zero (maximum total = 30).

The quality control team consisting of the investigators monitored the performance of field personnel, in 10% of participating schools, the quality control team independently cross-checked data on the school level check list.

### Statistical analysis

The summary statistics were reported as means, with standard deviations (SD) for continuous variables or percentages, with 95% confidence intervals (CI) for categorical variables. Descriptive statistics on student level and school level characteristics were calculated separately and comparisons made between intervention and control groups. For the comparison of student level variables, we conducted cluster-adjusted chi-squared tests for categorical variables and cluster-adjusted independent samples t-test for continuous variables. Variables significant at 0.25% level in the univariable analyses were included in the base model for model building.

We modelled a multivariable linear regression using percentage of post-intervention test score as the outcome variable under the Generalized Estimating Equation (GEE) framework to account for clustering by school, after adjusting for potential confounders and pre-intervention test score. We then used exchangeable correlation structure to adjust for clustering in the GEE model. In the initial model, we included all variables that were significant at 25% level, along with the main exposure variable (randomization arm) in the univariable analysis. A backward elimination method was subsequently used to remove the variables that did not have any confounding effect, i.e. could not make meaningful change (±10%) in the regression coefficient of the randomisation arm. Any variable that was not a confounder but significant in the multivariable model at 5% level was retained for increased precision of the estimates. After deciding on the final model, the variance inflation factor (VIF) was calculated to determine any evidence of multicollinearity.

To check the effect of the intervention on each answer to the 30 questions separately we also fitted a cluster-adjusted multivariable log-binomial model under the GEE framework. In these analyses the binary response (correct vs incorrect answer) for each question was used as the outcome variable to estimate the relative risk (RR) of giving a correct answer, and this was adjusted based on the pre-intervention result for the same question, on other potential confounders, and on significant variables, adopting the backward elimination approach as described above.

We also examined the cause of ‘lost to follow-up’ (did not appear in the post-intervention test) by fitting a log-binomial model using lost (yes/no) as the outcome variable and all other student level and school level variable in addition to the pre-intervention score as the explanatory variables. All the information for the explanatory variables was collected during the pre-intervention test. All the analyses were conducted in Stata version 13.

## Results

**Fig 1:**
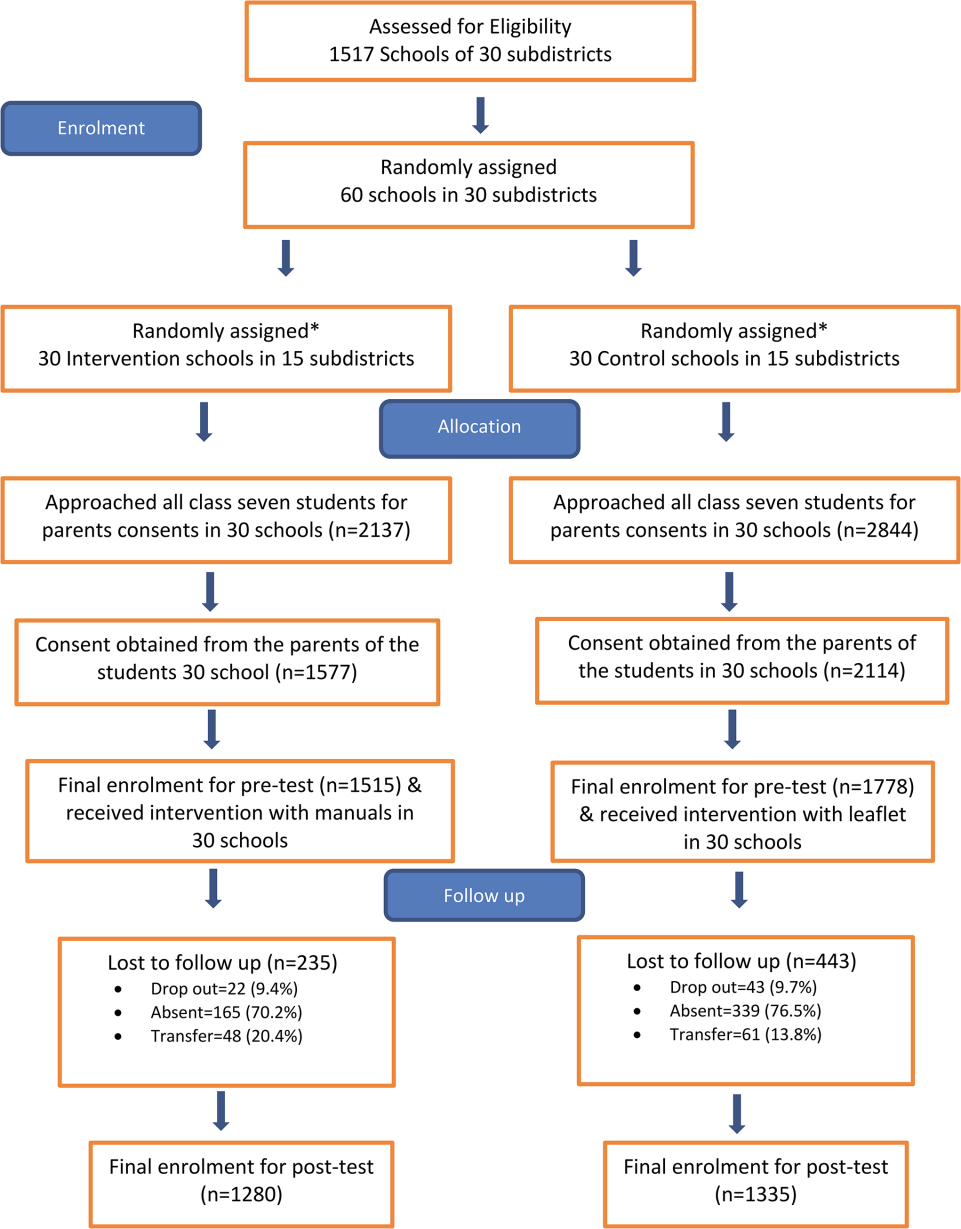
Flow chart of recruitment and follow up [Title of Fig 1]. *we randomized upazila (subdistrict) to assign as an intervention or control group.

A total of 3293 students from 60 schools from 30 subdistricts of seven coastal districts of Bangladesh were recruited for the trial. After randomization, 1515 students from 30 intervention schools and 1778 students from 30 control schools performed the pre-intervention test. The participant selection process for the trial is described in Fig 1. About 20.6% (24.9% in the control and 15.5% in the intervention group) of the students who sat for the pre-intervention test did not appear at the post-intervention test. None of the student-level variables including pre-intervention test score and school level variables were significantly different between the students who did and did not appear at the post-intervention test. Thus, we can conclude that the dropout was completely random, and that excluding the drop-out from the analyses would not bias the results.

None of the student level variables were significantly different between the control and intervention groups (Table 1). Only two of the 15 school level variables were significantly different between the groups (Table 2). A higher proportion of the intervention schools had a community clinic nearby (P = 0.007), and smaller proportion of intervention sites had a plantation program (P = 0.001) before the activities of CCHPU. This demonstrates that the randomization was effective. Most of the school children (86%) came from a low income family (<BDT 15000 equivalent to < US $200 monthly). The family head was farmer or day labourer in 45% families, a service holder in 20%, and a small and medium business holder in 21%. The father was the head in 93% of families and the majority lived in non-brick *kacha* (fence and corrugated sheets) or *mud* houses (Table 1).

**Table 1:** Descriptive statistics of the participants (n = 3293) at enrolment.

Variable	Intervention (n = 1515) %	Control (n = 1778) %	*p value* ^*1*^	ICC^2^ (95% CI)
**Gender (Male)**	36.4	42.9	0.20	0.12 (0.07–0.17)
**Number of family members (Mean ±SD)**	5.45 ±1.60	5.50 ±1.68	0.80	0.16
**Type of Family (single)**	80.6	78.3	0.41	0.04 (0.02–0.07)
**Gender of family head (Male)**	94.32	93.87	0.75	0.03 (0.01–0.04)
**Education of family head**			0.46	0.12 (0.07–0.17)
No formal	24.1	24.6		
Primary	32.8	29.1		
Secondary	20.15	27.5		
HSC	14.86	13.2		
Graduate and above	8.06	5.4		
**Occupation of family head**			0.06	0.06 (0.03–0.08)
Farmer	35.25	24.69		
Day labourer	13.20	17.94		
Service holder	16.90	23.85		
Small and medium business	22.77	21.09		
House wife	4.42	4.56		
Fisherman	2.44	1.97		
Unemployed	1.72	1.74		
others	3.30	4.16		
**Relation with family head**			0.59	0.04 (0.02–0.06)
Father	93.08	93.14		
Mother	5.02	5.79		
sister	0.33	0.11		
Brother	0.26	0.45		
Other	1.39	0.51		
**Age of Family Head (Mean ±SD)**	44.28 ±8.6	44.30 ±7.5	0.97	0.03
**Type of House**			0.31	0.33 (0.23–0.42)
Kachca	59.67	43.42		
Pakka	8.58	13.67		
Semi pakka	17.10	21.71		
Mud house	12.34	20.02		
others	2.31	1.18		
**No. of rooms (Mean ±SD)**	2.69 ±1.54	3.07 ±1.68	0.14	0.28
**Total household monthly income**			0.95	0.22 (0.14–0.29)
Income (cat)				
<5000BDT	16.50	17.83		
5000–8000	31.55	28.07		
8000–12000	22.44	23.40		
>12000	29.50	30.71		
**Density (person/room)**			0.30	0.27 (0.19–0.35)
1 per room	11.75	14.34		
2 per room	37.69	44.66		
3 per room	21.91	25.14		
4 Per room	11.55	8.49		
>4 per room	17.10	7.37		

^1^ For categorical variables P-value was obtained from cluster adjusted chi-square tests; for continuous variables from cluster adjusted independent samples t-test.

^2^ Intra-cluster coefficients

**Table 2:** Comparison of school (cluster) level characteristics.

Variable	Intervention (n = 30) %	Control (n = 30) %	*p-value* ^*1*^
**Type of school Roof**	73.33	66.67	0.57
**Availability of Electricity**	60.00	80.00	0.09
**Computer**	50	60	0.43
**Multimedia**	10	20	0.27
**Internet**	43.33	40	0.79
**Shelter in last 5 years disaster**	46.67	56.67	0.43
**1–20 days use as shelter**	71.43	94.12	0.08
**Disaster damage in last 5 years**	76.67	73.33	0.76
**Nearby community clinic**	80	46.67	0.007
**Any Plantation programme before CCHPU**	16.67	56.67	0.001
**Mosquito control activity around the school**	0.00	6.67	0.15
**Regular health education programme**	40	30	0.41
**Communication between school and community clinic**	16	30	0.22
**Drinking water from tubewell**	86.67	80	0.49
**Number of class seven students per usable latrine (Mean ±SD)**	17 ±10.37	14 ±7.60	0.18

^1^ For categorical variables, P-value was obtained from chi-square test; for continuous variables from independent samples t-test.

A comparison of school (cluster) level characteristics (Table 2) revealed that availability of electricity and use of computer and multimedia were lower in intervention than control schools. Half of the schools were used as *shelters* in disasters during the last 5 years, 47% in the intervention and 57% in the control groups. The proportion of schools damaged by a disaster during the last 5 years was slightly higher in intervention (77%) than control schools (73%). There was no mosquito control activity around the schools in the intervention area, whereas it occurred around 7% of control schools. Regular health education programs and communication between schools and community clinics were rarely provided in both groups (n.s.).

### Post-test total climate change knowledge score was 17% higher in intervention students

The variables occupation of family head, population density at home (person per room) (Table 1), nearby community clinics and plantation programmes for the school students (Table 2) were found significantly (at 25% level) different in the univariate model between the control and intervention groups and they were included in the multivariable random effect models. After adopting the backward elimination approach for model building the final model contained pre-intervention test score, occupation of the family head and population density at home. The final model revealed that students in the intervention school obtained 17.42% (95%CI: 14–20, P<0.001) higher marks in the post test compared to the control school. Students whose family head is a house wife had the lowest score. We found an inverse association between population density and post intervention marks (regression coefficient = − 0.41, P = 0.002) (Table 3).

**Table 3:** Multivariable random effects linear regression analysis of total score.

Variable	Regression Coefficient (95% CI)	*p-value*
**Group (intervention/control)**	17.42 (14.45 to 20.38)	< 0.001
**Pre-intervention score**	0.14 (0.10 to 0.19)	<0.001
**Occupation**		
Farmer		
Day labourer	1.24 (0.06 to 2.41)	0.03
Service holder	1.66 (0.58 to 2.74)	0.003
Small and medium business	1.18 (0.15 to 2.21)	0.02
House wife	-0.47 (-2.23 to 1.29)	0.60
Fisherman	1.34 (-1.21 to 3.89)	0.30
Others	1.79 (0.10 to 3.48)	0.03
**Population density in household (person per room)**	-0.41 (-0.68 to -0.14)	0.002
**Nearby community clinic (within 500 sq m)**	-2.48 (-5.66 to 0.69)	0.12
**Plantation programme before the intervention**	1.65 (-1.62 to 4.93)	0.32

### Health effect of climate change, adaptation and mitigation domain score about 2 times higher in intervention group

Table 4 presents the cluster adjusted Relative Risks for all 30 questions. The intervention group had a higher probability of answering all the questions correctly compared to the control group. Health effect of climate change knowledge score increased almost 2 times from baseline to follow up in the intervention group in comparison to control group, as per multivariable log-binomial regression analysis. The intervention group understood more than two times better than the control group that with the present school curriculum it will be difficult to adapt so the health effects of climate change are minimised (IRR 2.21,95% CI 1.36 to 3.60; p<0.001). Questions on adaptation for health and mitigation of climate change showed a statistically significant increased score in the intervention group than the control group.

**Table 4:** Multivariable random effects log-binomial regression analysis of knowledge level for individual questions.

Variable	Relative risk (95% CI)	*p-value*
**Ozone for global warming-q5**	1.33 (1.06 to 1.67)	0.01
**Ultraviolet ray cause skin cancer-q6**	1.30 (1.07 to 1.58)	0.008
**Ultraviolet ray cause eye cataract-q7**	1.26 (1.02 to 1.56)	0.02
**Increase of CO**_**2**_ **since 1970-q8**	2.16 (1.70 to 2.75)	<0.001
**Decadal Increase of global temperature for next century-q9**	1.98 (1.53 to 2.57)	<0.001
**Increase of vectors due to climate change-q12**	1.34 (1.15 to 1.55)	<0.001
**Vector of Dengue-q14**	1.96 (1.42 to 2.71)	<0.001
**Increase of Diarrhoea by 2020-q16**	1.52 (1.18 to 1.95)	<0.001
**School curricula to combat climate change and health-q19**	2.21 (1.36 to 3.60)	<0.001
**Adaptation for health-q20**	1.47 (1.11 to 1.96)	0.007
**Mitigation for health-q21**	1.43 (1.09 to 1.88)	0.009
**Tree plantation to reduce GHG-q22**	1.42 (1.17 to 1.73)	<0.001
**Solar system as renewable energy-q25**	1.17 (1.01 to 1.36)	0.03
**Sundarbon mangrove forest-q26**	1.50 (1.22 to 1.84)	<0.001
**Global reserve of water 97% saline sea-water-q28**	1.32 (1.06 to 1.64)	0.01
**Carbon sink process-q29**	1.38 (0.99 to 1.93)	0.05

The thirty outcome measuring True-False questions are described in Table 5 with their relevance of climate change in the context of the vulnerability of Bangladesh.

**Table 5:** The outcome measuring questionnaire with climate change domain and vulnerability of Bangladesh.

Q.No	True-False questions for pre and post test	Climate Change Domain	Vulnerability of Bangladesh^1^ ^.^ ^2^
1.	There is difference between climate and weather.	Climate and weather	Tropical Monsoon climate
2.	Global warming is not related with climate change	Global warming	Temperature increased in past century
3.	Greenhouse effect is the main cause of climate change.	Climate Change	Low emitting high risk LDC
4.	Carbon di-oxide is a human-generated (anthropogenic) greenhouse gas.	Greenhouse gas	Bangladesh emit 0.15% CO_2_ of global share
5.	Ozone gas is responsible for global warming.	Greenhouse gas	Phasing out ozone depleting substances
6.	Ultraviolet rays can cause skin cancer.	Climate and health	Skin cancer death 0.3/100000
7.	Ultraviolet rays can cause cataract of eye.	Climate and health	Child cataract 31%, adult 650K
8.	During 1970–2004 annual emission of carbon-di-oxide grew 60% globally.	Greenhouse gas	Long-term stable carbon intensity in emission
9.	Average global temperature is expected to rise by 1 degree Celsius per decade over the next 100 years.	Global warming	Projected ↑ 1.6°C, 2050
10.	Sea level rose, on average, 1 inch per year during1993-2005.	Sea level rise	South central part SLR 3.9mm/year
11.	Climate change is an environmental issue and it has no direct impact on health.	Climate and health	Health is one of the major risk sector (2^nd^ National Communication)
12.	A change in climate will be more favourable for growth of vectors such as mosquitos and rodents.	Climate and health	Temporal and spatial changes occurring for ecology of vectors
13.	Malaria is a vector borne disease.	Climate and health	A total of 14.7 million people are at risk
14.	Dengue is a spread by anopheles mosquito.	Vector borne disease	Outbreaks are associated with seasonal rainfall and relative humidity
15.	Changes in the frequency of extreme weather events such as cyclones, floods, storms, cold spells, and heat waves increase injuries and death.	Climate and health	Tropical cyclones, storm surges and flood displaced millions of people and damaged huge infrastructures in last 25 years; only cyclone SIDR in 2007 loss was 1.65 billion US$.
16.	Climate change can increase death by 2–5% due to diarrhoea by 2020.	Water borne disease	According to WHO estimate a 17% climate attributable diarrhoea will increase.
17.	Decrease in Food production would lead to widespread malnutrition.	Malnutrition	Over 2005–2050 a total cumulative loss of 80 million metric ton of rice may occur (2^nd^ National Communication).
18.	Displacement of population due to disaster can cause mental health problems.	Mental health	National mental disorder prevalence is 16.05%in adult population, (PTSD, Post Traumatic Stress Disorder is also high), and displacement is one of the risk factor.
19.	We can deal with the health problems of climate change with our present school curriculum.	Health adaptation	Present school curriculum doesn’t contain any environmental health topic focusing climate change
20.	Reducing the causes of climate change and its consequences on human health is known as ‘adaptation’.	Mitigation	Although Bangladesh is low emitting but population density is very high, emission could increase
21.	Improving the capacity to cope with the health risks by being better prepared is known as ‘mitigation’.	Health adaptation	As per the Bangladesh Climate Change Strategy and Action Plan (BCCSAP) thematic area one identified need for Health adaptation.
22.	Tree plantation cannot reduce greenhouse gases directly.	Mitigation	Deforestation is increasing due to decrease in arable area.
23.	We can save safe water simply by making a few changes in our daily life.	Health adaptation	Salinity intrusion in coastal area and arsenic in ground water source causes scarcity in safe drinking water.
24.	Reduce; Re-use and Recycle are three ‘R’ principles for calculating carbon footprint.	Mitigation	The 3R policy is not practiced in lifestyles.
25.	Solar power is a renewable energy source.	Renewable energy	Bangladesh is low power consuming country and renewable energy contributes only 1% to actual generation.
26.	Sundorban mangrove forest constitutes 40% of total Bangladesh forest.	Forest Conservation	Conservation of reserve forest, wild life reserves, plant and animal habitat is under threat.
27.	Air, sound, soil, river and water pollution causes diseases.	Environmental Pollution	Environmental pollution has attributed in increasing burden of diseases.
28.	Ninety seven percent of the total global water is ocean water.	Water reserve	There is implication of hydro climatic influences on seasonal and spatial transmission of diarrhoeal diseases.
29.	Carbon footprint is the natural mechanism that removes carbon dioxide from the atmosphere.	Carbon sink	Carbon sink in reserve forest is under threat in Bangladesh.
30.	Carbon sink is the measure of the amount of carbon dioxide emitted through the combustion of fossil fuels.	Carbon footprint	There is lack of awareness among citizen about the calculation of carbon foot print.

^1^Second National Communication of Bangladesh to the United nations Framework Convention on Climate Change, Ministry of Environment and Forests; October 2012.

^2^Bangladesh Environment and Climate Change Outlook 2012, Department of Environment; June 2013.

## Discussion

This intervention trial showed that a novel educational intervention providing manuals for school children achieved a significant increase in the knowledge about climate change and its relation to current and future health risks, as well as the adaptive measures. The improvement in all outcomes was higher in the intervention group who had received formatted training as per the manual, compared to the control group.

Children spend many hours a day in schools, and schools play a central role in teaching life skills [18–20]. Health promotion in schools and preparation of children to be [21] adaptation activists can bring substantial changes in knowledge, attitude and practices to address climate change issues and effect risk reduction of adverse health outcomes [22–26].

The two systematic reviews that were recently published do not report on any climate change-related school-based interventions [27, 28]. We could not find any studies similar to ours, and not much published evaluation [29].

Similar school-based trials on health interventions other than climatic relationship were found to be effective for school children [38–39]. A cluster-randomised controlled trial of a novel educational intervention to increase nutrition knowledge in 38 state primary school of Cambridgeshire, UK, comprising 2519 children in class 5 and 6 (aged 9–11 years) showed that the nutrition knowledge score was higher in intervention than in control schools [40]. In another cluster-randomised trial in New Zealand primary schools, the addition of hand sanitizers in classrooms compared with usual hand hygiene did not prevent disease of a severity sufficient to cause school absence [41]. Given the similar age group of children (grade level 7), we can compare our study with the Guangzhou, China, cluster-randomised trial on a school-based prevention programme on adolescent cigarette smoking, which had improved smoking-related knowledge but did not change students’ attitudes towards smoking [22]. Our study is also consistent with outcomes from a trial on the effect of sex education on high school students in Belize, which observed changes in knowledge only, not in the domains of attitude and behavioural intent [20].

Our study has several strengths. The cluster-randomised intervention design reduces contamination that would arise if children within the same classroom or school were allocated randomly to the intervention or control group. Strength of this study lies in the fact that the randomisation of schools for receiving the intervention has evenly spread unknown and known confounders, so that they do not impact on our results. For settings with limited resources, effective school-based interventions are essential for sustainability [42]. This randomised trial is the first of its kind in to evaluate the effectiveness of a manual generated to improve the climate change knowledge of children attending school. The manual is written in a simple language that is suitable for both school children and their parents or family members. Furthermore, the study recruited participants from an array of geographic locations known for their susceptibility to climate change. This makes the findings from this study relevant to school children living in other climate-vulnerable areas of Bangladesh.

There was adequate concealment of the randomisation (reducing the possibility of selection bias), and assessors of answers to questionnaires were blind to group allocation (reducing the possibility of detection bias). A further strength is the collection of drop-out data in the post-intervention test, which was unlikely to be affected by selection, detection or performance biases.

There were a few limitations of the study, including the short period of time between assessments. After the intervention, children were given a period of only six months before being re-tested on their understanding of climate change. This interval may not have been sufficient to lead to a sustainable improvement in relevant knowledge that will eventually be achieved. Further knowledge improvement may occur, as the students will be able to retain and consult the manual for another three years of their high school life. We acknowledge that we did not assess attitude and behaviour change; the study demonstrates value in ensuring retention of important technical information, but its influence on behaviour and safety has yet to be proven.

In schools in rural Bangladesh the drop-out rate and absenteeism is quite high (>30%), causing the ‘lost to follow up’ (20.58%) in this trial. As a whole the knowledge improvement of the intervention group was higher than of the control group; however, some of the students in the control schools also significantly improved their knowledge, despite being geographically distant from the intervention. That may be attributable to the teachers who received training on the manuals, and to information outsourcing, e.g. to television and newspapers. Our intervention was only tailored at the school and class level rather than to individuals, which may have diluted the observed effect of the intervention.

## Conclusion

As our results suggest, a child-centred adaptation strategy through school-based intervention can enhance the adaptive capacity of the future generation in the vulnerable community of South East Asia, specially in the high-risk context of Bangladesh. The manual could be scaled up to be provided to all schools in the Least Developed Country context, for mainstreaming the risk awareness in the education system and to protect human health from climate change. Future research in this area should include instruments to measure attitude, behavioural intent and practice of adaptation options. Further studies could be done to evaluate the cost-effectiveness of such an intervention and its longer term impact on the population.

## Supporting Information

S1 Questionnaire Full questionnaire in English with checklist. (DOC)

S1 Information Sheet RCT Infosheet in English. (PDF)

S1 Consent RCT Consent Form in English. (PDF)

S1 Manual Chapter content of manual in English. (DOC)

S2 Manual Student manual in Bangla. (PDF)

S1 Leaflet School RCT control-leaflet in English. (DOC)
