# Supplementary material for: Child Centred Approach to Climate Change and Health Adaptation through Schools in Bangladesh: A Cluster Randomised Intervention Trial
Source: PLoS One. 2015 Aug 7;10(8):e0134993. doi: 10.1371/journal.pone.0134993 (PMC4529232; doi:10.1371/journal.pone.0134993)
Supplement: S1 Questionnaire — (DOC) [file pone.0134993.s001.doc]

| Sample ID: |  |  |  |  |  |
| --- | --- | --- | --- | --- | --- |

**Risk Reduction of Climate Change Impact on Health Sector trough finding out adaptive Measures in the Context of Bangladesh.**

**School RCT Questionnaire**

January-2012

**Implemented by**

**Climate Change and Health Promotion Unit (CCHPU)**

Under

**Ministry of Health & Family Welfare**

14/2, Topkhan Road, Ansari Bhaban, Dhaka-1000.

Bangladesh

In Collaboration with

**The University of New Castle, Australia**

**And Siam Health Foundation**

**BASELINE QUESTIONNAIRE**

Risk reduction of climate change impact on health sector

through finding out adaptive measures in the context of Bangladesh.

School Based Clustered Randomized Intervention Trial

Survey Date:.....................

|  |  |  |
| --- | --- | --- |

1. Participant Name : ................................................ Code No.

|  |  |  |
| --- | --- | --- |

2. District Name : ................................................ Code No.

|  |  |  |
| --- | --- | --- |

3. Upozila Name : ................................................ Code No.

|  |  |  |
| --- | --- | --- |

4. Union/Word : ................................................ Code No.

|  |  |  |
| --- | --- | --- |

5. School name: .................................................................. Code No

1. Name of the student: Roll number :

1. Total number of students in class VII :
2. Name of the Class Teacher:

Surveyor Name and Signature :.....................................

Family status

1. What is the size of your family? _____
2. What is the type of your family?
3. Single unit ii. Joined

[only parents=single unit; uncles, aunts, grandparents=Joined]

1. Information of family members

| Sl. | Name of family memebr | Relation with the student | Sex | age | Education | Occupation |
| --- | --- | --- | --- | --- | --- | --- |
| 1 | 2 | 3 | 4 | 5 | 6 | 7 |
|  |  |  |  |  |  |  |
|  |  |  |  |  |  |  |
|  |  |  |  |  |  |  |
|  |  |  |  |  |  |  |
|  |  |  |  |  |  |  |
|  |  |  |  |  |  |  |
|  |  |  |  |  |  |  |
|  |  |  |  |  |  |  |

For column 3,4,6,7 write down this coding

| Code (Relation ) | Code (sex) | Code (Education) | Code (Occupation) |
| --- | --- | --- | --- |
| 1. Father 2. Mother 3. Brother 4. 4sister 5. others | 1. male 2. Female | 1. No formal education 2. Primary 3. Secondary 4. SSC 5. HSC 6. Graduation or more | 1.Farmer  2.Day labour  3.Service holder  4.Small business  5. Housewife  6. Unemployed  7. Student  8. Fisherman  9. Others |

1. How many rooms are there in your house? ………..
2. What is the type of your house?
3. Kancha (Fencing and corrugated sheet) ii. Brick building iii. Semi-building ( brick wall, roof with corrugated sheet) iv. Mud house v. Others
4. What is your average family income ? (include all members income)

……………..

[ Note: If the any student is unable to provide information on this , then interviewer write down the question on a paper and send it to home with the student to bring it back next day from the parents to the class teacher. Next day the data collector will fill up accordingly.]

Name of Data collector:

Signature:

Knowledge about climate change (True/ False questions)

True False

1. There is a difference between Climate and Weather? □ □
2. Global warming is not related with climate change? □ □
3. Green house effect is the main cause of climate change? □ □
4. Carbon di-oxide is a human-generated (anthropogenic)

green house gas. □ □

1. Ozone gas is responsible for global warming?
2. Ultraviolet rays can cause skin cancer ? □ □
3. Ultraviolet rays can cause cataract of eye? □ □
4. Between 1970-2004 annual emission of carbon-di-oxide

grew 60% globally? □ □

1. Average global temperature is expected to rise by 1 degree Celsius

per decade over the next 100 years? □ □

1. Sea level rose,on average, 1 inch per year between 1993-2005? □ □
2. Climate change is an environmental issue and it has no direct

impact on health? □ □

1. A change in climate will be more favourable for growth of vectors such as mosquitos and rodents? □ □
2. Malaria is a vector borne disease? □ □
3. Dengue is a spread by anopheles mosquito?
4. Changes in the frequency of extreme weather events such as cyclones, floods, storms, cold spells, and heat waves increase injuries and death? □ □
5. Climate change can increase death by 2-5% due to diarrhea by 2020? □ □
6. Decrease in Food production would lead to widespread malnutrition? □ □
7. Displacement of population due to disaster can cause mental health problems? □ □
8. We can deal with the health problems of climate change with our present school curriculum? □ □
9. Reducing the causes of climate change and its consequences on human health is known as ‘adaptation’? □ □
10. Improving the capacity to cope with the heath risks by being better prepared is known as ‘mitigation’? □ □
11. Tree plantation can not reduce greenhouse gases directly? □ □
12. We can save safe water simply by making a few changes in our daily life. □ □
13. Reduce, Re-use and Recycle are three ‘R’ principles for

calculating carbon footprint. □ □

1. Solar power is renewable energy source. □ □
2. Sundorbon mangrove forest constitutes 40% of total Bangladesh forest.
3. Air, sound, soil, river and water pollution causes diseases. □ □
4. Ninety seven percent of the total global water is ocean water. □ □
5. Carbon footprint is the natural mechanism that removes carbon dioxide from the atmosphere. □ □
6. Carbon sink is the measure of the amount of carbon dioxide emitted through the combustion of fossil fuels. □ □

**Common Checklist on School survey**

School Name: Code No.

| District Code: |  | Upozilla Code: |  | Union Code: |  | School Code: |  |
| --- | --- | --- | --- | --- | --- | --- | --- |

**Surveyor observe and tick (√) them**

1. Type of Roof of the school?

I. Pucca

II. Tin

III. Other ( specify)

2. Electricity ?

I. Yes

II. No

3. Source of Drinking Water?

I. Shallow

II. Deep Tubewell

III. Supplied water through Pipe

IV. Crude pond water

V. Refined Pond water

VI. Rain water

VII. Other

4. Is there any sanitary latrine in this school?

I. Yes

II. No

(if No skip)

5. How many usable latrines are there?

I. One

II. Two

III. More than two

IV. None

6. Govt. Hospital nearby?

I. Dist. Hospital

II. Upazilla Health Complex

III. Union Health Center

IV. Comm. Clinic

V. Other (Specify)

7. Any Health Education Program in this school?

I. Yes

II. No

8. Community Clinic in the Survey Area?

(If Yes then Name)

I. Yes

II. No

9. Exchange/Communication between School and Community Clinic?

I. Yes

II. No

10. Mosquito Crash Program locally?

I. Yes

II. No
