# Supplementary material for: Child Centred Approach to Climate Change and Health Adaptation through Schools in Bangladesh: A Cluster Randomised Intervention Trial
Source: PLoS One. 2015 Aug 7;10(8):e0134993. doi: 10.1371/journal.pone.0134993 (PMC4529232; doi:10.1371/journal.pone.0134993)
Supplement: S1 Information Sheet — (PDF) [file pone.0134993.s002.pdf]

Participant Information Statement for the Research Project:

Risk Reduction of Climate Change Impact on Health Sector trough finding out adaptive Measures in the Context of Bangladesh: School based clustered randomised intervention trial

Investigators:

Dr. Abul Hasnat Milton, The University of Newcastle, Australia

Dr. Bayzidur Rahman, The University of New South Wales, Australia

Professor Wayne Smith, The University of Newcastle, Australia

Dr. Iqbal Kabir, The University of Newcastle, Australia and BRAC, Bangladesh

Dear---

We would like to invite you to participate in the research on Risk Reduction of Climate Change Impact on Health Sector trough finding out adaptive Measures in the Context of Bangladesh. The research is part of Dr. Iqbal Kabir's PhD studies at the University of Newcastle, supervised by Dr. Abul Hasnat Milton and co-supervised by Professor Wayne Smith, Conjoint Professor l from the School of Medicine and Public Health, Faculty of Health, Centre for Clinical Epidemiology and Biostatistics, University of Newcastle, Australia and Dr. Bayzidur Rahman from the The School of Public Health and Community Medicine, Faculty of Medicine, The University of New South Wales, Australia.

Why is the research being done?

The objective of the study is to assess the perception of the vulnerable population of coastal area of Bangladesh on the effects of climate change and health; and to determine the effectiveness of a child centred approach as a part of adaptive measures to the climate change

and health among the high school students and teachers of vulnerable areas. The findings of this study will provide a solid basis of the significance of climate change impact on health and effectiveness of the adaptive strategies for nationwide scale up of the activities.

Who can participate in the research?

We have selected the school in which your child is studying in class seven. We will then train them on climate change following a module. Before and after the training, we would like to interview your child to collect information regarding this topic.

Who can participate in the research?

Your child's participation in this study completely depends on your willingness. Your child will be included in this study only once you have given the consent .If you want to discuss with other members of your household before participating in this interview, we can do it later. If you wish your child not to take part in this study, we are very sorry to take your valuable time. We would like to assure you that your denial to participate in the study will not affect your child from any aspect. You also have the option of withdrawing any information which identifies your child.

What would you be asked to do?

If you allow your child to participate in this study then we will request your child to participate in a training programme based on climate change related module. We will collect some information through a face to face interview before the training. The child will also need to participate in a pre and post test before and after the training programme. Before your child's participation in this study, you will have to sign or put your thumb impression on a consent form .Then our interviewer will ask your child a few questions using a standard questionnaire which will include your socioeconomic status. Your child will then receive a training on climate

change. The duration of the total study will be of six months. The duration of the training in total will be less than a week in total. Our interviewer will require 20 minutes to complete the initial interview. You or your child will not get any direct benefit through participating in this interview. Confidentiality will be maintained on your child's information provided and it will only be accessed and used by the researchers, not by others except if is there any legal issue.

How will your privacy be protected?

All the information collected will be kept in the office of the Centre for Health and Development (CHAD), Dhaka Office for 5 years and one soft copy will be sent to the University of Newcastle for analysis and will be kept there. The interview sheet will be burnt and all data kept in the computer will be deleted after five years .Your permission will be sought before using this data in future again. All the information collected for this study will be will not disclose any name without prior permission.

The result of the study will be published as report and it will also be disseminated in scientific journals and conferences. Confidentiality will be maintained for every participant .The summery findings will be distributed among the participants through the selected schools.

How will the information collected be used?

You are requested to read this information sheet carefully and please make sure that you understood all the points discussed here. Please communicate with the researchers, if you have any difficulty to understand any section clearly or if you have further query. After considering all this points if you agree for your child to participate in this study please kindly fill up the consent form and give us your permission to proceed for the interview and training programme as per the research plan.

If you have further query ,please communicate with Dr. Dr. Iqbal Kabir, Climate Change and Health promotion unit, Ministry of Health and Family Welfare, Bangladesh, Phone :+ 88 01714 165 204.

Please also note that this project has been approved by the University of Newcastle's Human Research Ethics Committee, Approval No. H- ……… ….If you have concerns about your rights as a parent/carer of the child participant in this research, or have a complaint about the manner in which the research is conducted or if you would like further information please contact Professor Mahmudur Rahman Director, IEDCR(Mobile:01711595139).

Thanking you.

Sincerely,

Dr. Abul Hasnat Milton

Principal Investigator

School of Medicine and Public Health

Faculty of Health, CCEB

Level-3, David Maddison Building,

Cnr of King and watt Street, Newcastle,

NSW 2300, Australia.

Telephone: + 612 49138200

Fax: + 61 2 49138148

Email address: Milton.hasnat@newcastle.edu.au

Dr. Md. Iqbal Kabir

PhD Candidate, Student investigator

School of Medicine and Public Health

University of Newcastle, Australia

Email : mdiqbal.kabir@uon.edu.au

THE UNIVERSITY OF
NEWCASTLE
AUSTRALIA
