# Supplementary material for: Child Centred Approach to Climate Change and Health Adaptation through Schools in Bangladesh: A Cluster Randomised Intervention Trial
Source: PLoS One. 2015 Aug 7;10(8):e0134993. doi: 10.1371/journal.pone.0134993 (PMC4529232; doi:10.1371/journal.pone.0134993)
Supplement: S1 Consent — (PDF) [file pone.0134993.s003.pdf]

Risk Reduction of Climate Change Impact on Health Sector trough finding out adaptive Measures in the Context of Bangladesh: School based clustered randomised intervention trial

Consent Form

Bangladesh :
Bangladesh :
Dr. Md Iqbal Kabir
Climate Change and Health promotion unit,
Ministry of Health and Family Welfare, Bangladesh,
Phone :+ 88 01714 165 204.
Email: mdiqbal.kabir@uon.edu.au

Australia:
Dr. Milton Hasnat ,
Senior lecturer, School of Medicine and Public Health, The University of Newcastle, Newcastle, NSW 2308, Australia. Telephone: + 61 2 49138200 .
Email address: Milton.hasnat@newcastle.edu.au

I have been informed and understood details of the Information sheet and then consented for my child to participate in this study. I hope this study will be conducted as mentioned in the information sheet. Name of the person who has explained the information for me is,

………………………………………………………………………………..
I understood that at any time during this study period my child can withdraw him/herself without stating any reason and it will not influence me anyhow. I can withdraw all data received from my child within one month of the interview if I wish to. I grant permission to fill up questionnaire through Interviewer.

I believe all collected personal information during study period will be preserved with confidentially by the researcher. I got enough opportunity to understand the Information which was mentioned in the Information sheet.

Participant’s Name:

Parent/Carer's Name:

Address: ______________________________________

Parent/ Carer's Signature/Thumb Impression: Date:

I have read the Information sheet which has been shared with the study participant's parent/carer and I am sure that the participant's parent/carer understood these Information.

Explainer's Name:

Explainer's Signature: Date:
