# Supplementary material for: Child Centred Approach to Climate Change and Health Adaptation through Schools in Bangladesh: A Cluster Randomised Intervention Trial
Source: PLoS One. 2015 Aug 7;10(8):e0134993. doi: 10.1371/journal.pone.0134993 (PMC4529232; doi:10.1371/journal.pone.0134993)
Supplement: S1 Manual — (DOC) [file pone.0134993.s004.doc]

**Climate Change and Health Protection**

**Manual for the Students and their Family Members:**

The manual has seven chapters. Chapter one deals with issues of climate and how the climate is changing. Climate change and its effect on health, climate sensitive diseases and other environmental health issues have been discussed in chapter two. In chapter three, the risk management of health hazards due to Climate Change is addressed. Conservation of environment and natural resources of Bangladesh is the content of chapter four. Chapter five comprises climate change and health protection issues with three case studies. Chapter six describes how to reduce environmental pollution (air, soil, water, river, sound) and keep healthy life style. Chapter seven deals with 3 days practical lesson plans for climate change and health adaptation.

**Contents**

**Chapter 1 : Climate Change**

What is Climate Change? Cyclone

Flood, Precipitation

Drought, Global Warming, Natural Disaster, Effects of Green House

How Human-being Produce Green House Gas?

International Initiatives about Climate Change

**Chapter 2 : Climate Change and Health Problem**

Heat Stroke, Respiratory Disease

Water logging and Skin Disease, Hit or wound, Water Borne Disease

Vector Borne Disease, Malaria, Dengue

Japanese Encephalitis

Food Problem, Malnutrition

Psycho-social Stress

Climate Change and Correlation with Human Health

**Chapter 3 : Risk management of health hazards due to Climate Change**

Decreasing hazards due to Climate Change or Way of Mitigation

The Work We can easily do in Our Daily Life.

Prevention of Water Born Disease, Protection from Insect Born Disease

Stay Green, Save Paper, Use Renewable Energy

Re-cycle, Reduce, Re-use

Adaptation and resilience

**Chapter 4 : Conservation of Natural Resources**

Geographical Structure, Bio diversity

Sunderban ,Cox's Bazar

**Chapter 5 : Climate Change and Health Protection**

Case Study-1, Case Study-2, Case Study -3

Recap

Climate Change and Health Promotion Unit

**Chapter 6 : Reduce Pollution, Keep Healthy**

Air Pollution, Soil Pollution, River Pollution, Water Pollution

Sound Pollution, Water in Earth

**Chapter 7 : 3 Days Lesson Plan for Students**

1st Day Activities

2nd Day Activities

3rd Day Activities

Word Bank

Climate Change due to Human Involvement, Atmosphere, Bio-fuel

Carbon-di-oxide, Carbon Footprint, Climate Change, Carbon Sink

Greenhouse Gases, NAPA, UNFCC
