# Supplementary material for: Child Centred Approach to Climate Change and Health Adaptation through Schools in Bangladesh: A Cluster Randomised Intervention Trial
Source: PLoS One. 2015 Aug 7;10(8):e0134993. doi: 10.1371/journal.pone.0134993 (PMC4529232; doi:10.1371/journal.pone.0134993)
Supplement: S2 Manual — (PDF) [file pone.0134993.s005.pdf]

জলবায়ু পরিবর্তন ও স্বাস্থ্য সুরক্ষা

শিক্ষার্থী ও পরিবারের সদস্যদের জন্য সহায়িকা

CCHPU
Climate Change & Health Promotion Unit

ক্লাইমেট চেঞ্জ অ্যাণ্ড হেলথ প্রমোশন ইউনিট
স্বাস্থ্য ও পরিবার কল্যাণ মন্ত্রণালয়

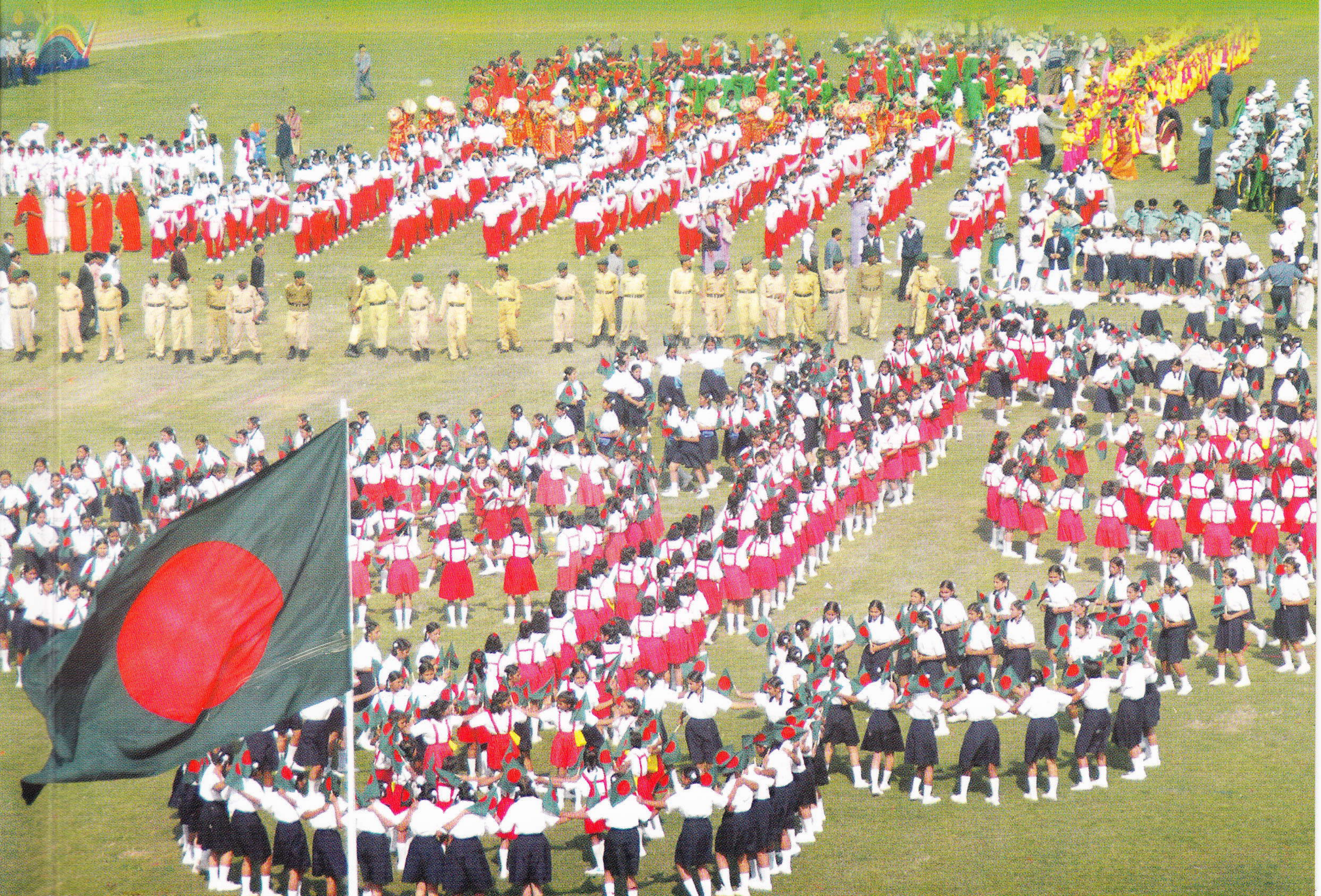

জলবায়ু পরিবর্তনজনিত দুর্যোগ মোকাবেলা

বন্যায় করণীয়

বন্যার পূর্বেই উঁচু স্থান যেমন- উঁচু বাড়ি, স্কুল ঘর, টিলা, আশ্রয়কেন্দ্র ইত্যাদি চিহ্নিত করে রাখো এবং নিচু স্থান যেমন- পুকুর, ডোবা, কুঁয়া ইত্যাদি ঘিরে রাখো।

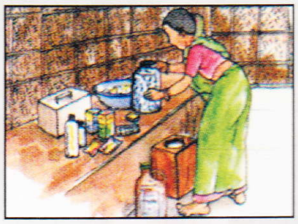

বন্যার পূর্বেই শুকনো খাবার, খাবার স্যালাইন, প্রাথমিক চিকিৎসার ঔষধপত্র এবং নিরাপদ পানি পর্যাপ্ত পরিমাণে সংগ্রহ করে রাখো।

বন্যাকালীন সময়ে অসুস্থ, আঘাতপ্রাপ্ত, সাপে কাটা ও পানিতে ডুবা মানুষদের উদ্ধার করে প্রাথমিক চিকিৎসা প্রদান করো এবং যত দ্রুত সম্ভব নিকটস্থ স্বাস্থ্যকেন্দ্রে বা হাসপাতালে প্রেরণ করো।

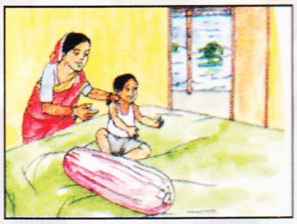

বন্যাকালীন সময়ে ছোটো শিশুদের চোখে চোখে রাখো যাতে পানিতে পড়ে ডুবে না যায় এবং সকল শিশুদের সাঁতার শিখতে উৎসাহিত করো।

ঝড়, সাইক্লোন ও বজ্রপাতে করণীয়

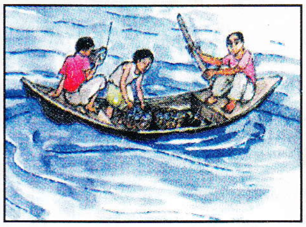

ঝড়ের বার্তা মেনে চলো, রেডিও শোনো এবং নৌযানে রেডিও, টর্চ লাইট, লাইফ জ্যাকেট, লাইফ বয়া ইত্যাদি রাখতে বলো।

ঝড়ের পূর্বাভাষ পাওয়া মাত্রই দ্রুত কোন নিরাপদ স্থান যেমন- উঁচু পাকা বাড়ী, বা আশ্রয়কেন্দ্রে চলে যাবে।

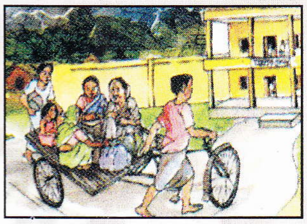

আশ্রয়কেন্দ্রে ঝুঁকিপূর্ণ জনগোষ্ঠী যেমন: বৃদ্ধ, নারী ও শিশুদের পরিবহনের জন্য যানবাহনের ব্যবস্থা এবং শুকনো খাবার ও জরুরী ঔষধপত্র সঙ্গে রাখো।

বজ্রপাতের সময় খোলা জায়গায় থাকা নিরাপদ নয়। এজন্য নিরাপদ স্থানে যেমন-বড় গাছের আড়ালে বা বড় দালান কোঠায় অবস্থান গ্রহণ করো।

রক্তক্ষরণ বন্ধ, ভাঙ্গা জায়গায় ব্যান্ডেজ ও শ্বাসকষ্টের সময় করণীয়

কেটে যাওয়া স্থান থেকে অতিরিক্ত রক্তক্ষরণে মানুষের মৃত্যু হতে পারে, তাই অবিলম্বে রক্তক্ষরণ বন্ধ করতে হবে।

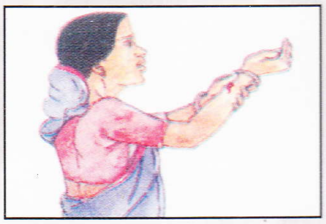

রক্তক্ষরণ বন্ধে কেটে যাওয়া অংশে সরাসরি চাপ দাও। ব্যান্ডেজ বাঁধো এবং আহত স্থান উচু করে ধরো। যত দ্রুত সম্ভব নিকটস্থ চিকিৎসক বা স্বাস্থ্যকেন্দ্রে প্রেরণ করো।

শরীরের কোন অংশ ভেঙ্গে গেলে ভাঙ্গা অংশটি যেন নড়ে না যায় সেজন্য হাতের কাছে যা পাওয়া যাবে যেমন বাঁশ, কাঠ এমনকি শক্ত কাগজ দিয়ে বেঁধে দ্রুত নিকটস্থ ডাক্তার বা স্বাস্থ্যকেন্দ্রে প্রেরণ করো।

দুর্ঘটনায় পতিত ব্যক্তির শ্বাস-প্রশ্বাস ঠিক রাখার জন্য তাকে খোলা জায়গায় বাম কাত করে শুইয়ে মাথা কাত করে মুখ খোলা রাখতে হবে। অথবা সোজা করে শুইয়ে সাবধানে কপাল ও চিবুক ধরে চিবুক উঁচু ও মাথা পেছনে ঠেলে মুখ হা করিয়ে দিতে হবে।

পোড়া, বিদ্যুৎস্পৃষ্ট ও পানিতে ডুবে গেলে করণীয়

রান্নার পর চুলা ভালভাবে নিভিয়ে রাখতে হবে। দাহ্য পদার্থ থেকে দূরে থাকো। জলন্ত কোনকিছু নিভিয়ে নির্দিষ্ট স্থানে ফেলো।

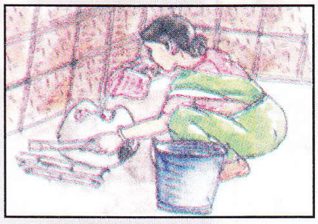

শিশুকে আগুন বা দাহ্য পদার্থ নিয়ে খেলতে দিবে না। পুড়ে গেলে আক্রান্তস্থানে প্রচুর পানি ঢালো এবং আক্রান্ত ব্যক্তিকে প্রচুর পরিমাণ পানি ও তরল পদার্থ খাওয়াও।

বৈদ্যুতিক সংযোগ সঠিকভাবে স্থাপন করা হয়েছে কিনা সে সম্পর্কে নিশ্চিত হও। ভেজা কাপড় বৈদ্যুতিক তারে কখনো শুকাতে দিবে না। বেআইনি ও ঝুঁকিপূর্ণ বৈদ্যুতিক সংযোগ নিবে না বা নিতে দিবে না।

কেউ পানিতে ডুবে গেলে তাকে উদ্ধারের জন্য নিজের নিরাপত্তা নিশ্চিত করে লাঠি, বাঁশ, গাছের ডাল, দড়ি এমনকি প্যাচানো চাদরের এক প্রান্ত শক্ত করে ধরে অপর প্রান্ত ডুবন্ত ব্যক্তির কাছে ছুড়ে ফেলো এবং ডুবন্ত ব্যক্তি সেটি ধরলে তাকে টেনে তুলো।

পরিবেশ ও বন মন্ত্রণালয়-এর জলবায়ু পরিবর্তন ট্রাস্ট ফাণ্ডের আওতায়
বাস্তবায়নাধীন প্রকল্পের অধীনে সহায়িকাটি প্রকাশিত

জলবায়ু পরিবর্তন ও স্বাস্থ্য সুরক্ষা

শিক্ষার্থী ও পরিবারের সদস্যদের জন্য সহায়িকা

ক্লাইমেট চেঞ্জ অ্যাণ্ড হেলথ প্রমোশন ইউনিট
স্বাস্থ্য ও পরিবার কল্যাণ মন্ত্রণালয়

জলবায়ু পরিবর্তন ও স্বাস্থ্য সুরক্ষা
শিক্ষার্থী ও পরিবারের সদস্যদের জন্য সহায়িকা

ক্লাইমেট চেঞ্জ অ্যাণ্ড হেলথ প্রমোশন ইউনিট
স্বাস্থ্য ও পরিবার কল্যাণ মন্ত্রণালয় কর্তৃক বাস্তবায়নাধীন প্রকল্পের আওতায়
সহায়িকা-টি ছাত্রছাত্রী ও শিক্ষকদের বিনামূল্যে বিতরণের জন্য

সংকলন ও প্রকাশনা তত্ত্বাবধান

জাতীয় শিক্ষাক্রম ও পাঠ্যপুস্তক বোর্ড (এনসিটিবি), শিক্ষা মন্ত্রণালয়
ক্লাইমেট চেঞ্জ ইউনিট (সিসিইউ), পরিবেশ ও বন মন্ত্রণালয়

এনসিটিবি মূল্যায়ন কমিটি

প্রফেসর তাহেরা আখতার জাহান, সদস্য (শিক্ষাক্রম), আহ্বায়ক
জারিয়া তুল হাফছা, গবেষণা কর্মকর্তা, সদস্য
ফাতেমা নাসিমা আখতার, গবেষণা কর্মকর্তা, সদস্য
শাহীনারা বেগম, বিশেষজ্ঞ, সদস্য-সচিব

সার্বিক তত্ত্বাবধান

মোঃ শফিকুল ইসলাম লস্কর, যুগ্ম-সচিব (জনস্বাস্থ্য ও বিশ্ব স্বাস্থ্য), স্বাস্থ্য ও পরিবার কল্যাণ মন্ত্রণালয়
এম মনজুরুল হান্নান খান, উপ-সচিব ও প্রকল্প পরিচালক, সিসিইউ, পরিবেশ ও বন মন্ত্রণালয়
মোঃ রাশেদুল ইসলাম, উপ-সচিব ও পরিচালক, সিসিইউ, পরিবেশ ও বন মন্ত্রণালয়
রাশেদা আকতার, উপ-সচিব ও প্রকল্প পরিচালক, সিসিএইচপিইউ, স্বাস্থ্য ও পরিবার কল্যাণ মন্ত্রণালয়
এস জি মাহমুদ, ন্যাশনাল প্রফেশনাল অফিসার, বিশ্ব স্বাস্থ্য সংস্থা

সম্পাদনা

ডা. ইকবাল কবীর, সমন্বয়কারী, সিসিএইচপিইউ

সহযোগিতা

জাহাঙ্গীর সেলিম, আলমগীর হোসেন, সাদিয়া আফরোজ, মার্জিয়া হক তানিয়া, সৈয়দ ইসতিয়াক আহমেদ, মোশাররফ হোসাইন, মির্জা ফায়সাল হোসেন

আলোকচিত্র

জিয়া ইসলাম, জাহাঙ্গীর সেলিম

কৃতজ্ঞতা স্বীকার

স্বাস্থ্য অধিদপ্তর, পরিবেশ অধিদপ্তর, বিশ্ব স্বাস্থ্য সংস্থা, আইইডিসিআর, নিপসম, বিসিএএস, কমিউনিকেশন কালচার

প্রকাশকাল : মার্চ ২০১১

প্রকাশক

ক্লাইমেট চেঞ্জ অ্যাণ্ড হেলথ প্রমোশন ইউনিট (সিসিএইচপিইউ)
স্বাস্থ্য ও পরিবার কল্যাণ মন্ত্রণালয়
আনসারি ভবন (পঞ্চম তলা)
১৪/২, তোপখানা রোড, ঢাকা-১০০০, বাংলাদেশ।
প্রকল্প কার্যালয় : ১৯৫/২/১, শান্তিবাগ, ঢাকা-১২১৭।

মুদ্রণ

ইন্টার প্রেস লিমিটেড, ৮৫/১ ফকিরাপুল, ঢাকা-১০০০।

শুভেচ্ছা বার্তা

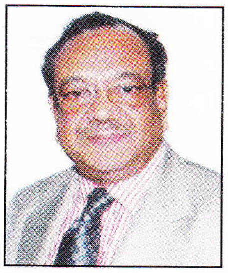

ডা. আ. ফ. ম. রুহুল হক এম.পি
মন্ত্রী
স্বাস্থ্য ও পরিবার কল্যাণ মন্ত্রণালয়
ঢাকা

জলবায়ু পরিবর্তন একটি বৈশ্বিক সমস্যা এবং এর প্রভাবে সারা পৃথিবীর মানুষ আজ বিপর্যস্ত। স্বাস্থ্যখাতে এর ঝুঁকির পরিমান ক্রমেই আরো প্রকট আকার ধারণ করছে। দক্ষিণ-পূর্ব এশিয়ায় পৃথিবীর এক-চতুর্থাংশ জনগোষ্ঠীর বসবাস, যাদের একটি বিরাট অংশ দরিদ্র। তাই জলবায়ু পরিবর্তনের প্রভাব এ অঞ্চলে বয়ে আনবে ব্যাপক ক্ষতি। ইতোমধ্যেই সংক্রামক ব্যাধি ব্যাপকভাবে বৃদ্ধি পেয়েছে, যা ভবিষ্যতে আরও বাড়বে বলে আশংকা করা হচ্ছে। এ অঞ্চলের বেশীরভাগ দেশেই জরুরী চিকিৎসাসেবা প্রদান, কীটপতঙ্গবাহিত রোগ প্রতিরোধ ইত্যাদি ক্ষেত্রে উন্নত অবকাঠামো নেই।

জলবায়ু পরিবর্তনের ফলে সবচেয়ে ক্ষতিগ্রস্ত দেশ হচ্ছে বাংলাদেশ। তাই স্বাস্থ্যের উপর জলবায়ু পরিবর্তনের ক্ষতিকর প্রভাব মোকাবেলায় আমাদের সকলকে সচেতন থাকতে হবে। ইতোমধ্যে বিভিন্ন আন্তর্জাতিক সম্মেলনে আমরা বিষয়টি তুলে ধরেছি এবং প্রাথমিক স্বাস্থ্য পরিচর্যা কেন্দ্রগুলোর মাধ্যমে জলবায়ু পরিবর্তনে স্বাস্থ্য সুরক্ষার ব্যবস্থা নিতে বেশ কিছু পদক্ষেপ গ্রহণ করেছি। আমার দৃঢ় বিশ্বাস, ভালোভাবে প্রস্তুত থেকে এবং সকলের সচেতন সচেষ্ট ভুমিকার মাধ্যমে জলবায়ু পরিবর্তনজনিত স্বাস্থ্য ঝুঁকি কমানো সম্ভব।

স্বাস্থ্য ও পরিবার কল্যাণ মন্ত্রণালয়ের অধীনে ক্লাইমেট চেঞ্জ অ্যাণ্ড হেলথ প্রমোশন ইউনিট (সিসিএইচপিইউ) নামে একটি স্বতন্ত্র ইউনিট এ লক্ষ্যে কাজ করে যাচ্ছে। পরিবেশ ও বন মন্ত্রণালয়ের ট্রাস্ট ফান্ডের আওতায় Risk reduction and adaptive measures in the context of climate change impact on health sector in Bangladesh নামে একটি প্রকল্প গ্রহণ করা হয়েছে।

স্বাস্থ্য ও পরিবার কল্যাণ মন্ত্রণালয়ের প্রকল্পের অধীনে জাতীয় শিক্ষাক্রম ও পাঠ্যপুস্তক বোর্ডের সহযোগিতায় স্কুল শিক্ষার্থী ও শিক্ষকদের জন্য দু'টো আলাদা সহায়িকা তৈরি হওয়ায় আমি আনন্দিত। আমি আশা করছি এই সহায়িকা দু'টো বিদ্যালয় ও কমিউনিটি ক্লিনিকের মধ্যে জলবায়ু পরিবর্তন ও স্বাস্থ্য সুরক্ষার সেতুবন্ধন হিসাবে কার্যকর ভুমিকা রাখবে।

সহায়িকাটি প্রণয়নে সংশ্লিষ্ট সকলকে আন্তরিক ধন্যবাদ জানাই।

জয় বাংলা

জয় বঙ্গবন্ধু

প্রফেসর আ. ফ. ম. রুহুল হক এম.পি
মন্ত্রী
স্বাস্থ্য ও পরিবার কল্যাণ মন্ত্রণালয়

CCHPU
Climate Change & Health Promotion Unit

শুভেচ্ছা বার্তা

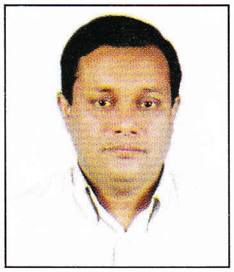

ড. হাছান মাহমুদ এম.পি
প্রতি মন্ত্রী
পরিবেশ ও বন মন্ত্রণালয়

জলবায়ু পরিবর্তন বর্তমান পৃথিবীতে একটি আলোচিত বিষয়। মানুষের বিভিন্ন কর্মকাণ্ডে বায়ুমণ্ডলের গ্রিনহাউস গ্যাসের পরিমান ক্রমশঃ বেড়ে যাচ্ছে। এর প্রভাবে পৃথিবীর তাপমাত্রা ধীরে ধীরে বাড়ছে। উন্নত বিশ্বের অতি উচ্চ মাত্রায় কার্বন ডাইঅক্সাইড নির্গমন বাড়িয়ে দিচ্ছে বৈশ্বিক উষ্ণতার হার। বাংলাদেশে নির্গত কার্বন-ডাই-অক্সাইডের পরিমান পৃথিবীর বার্ষিক কার্বন-ডাই-অক্সাইডের নিঃসরনের মাত্র ০.১৫ ভাগ। অথচ বাংলাদেশ সবচেয়ে বেশী ভুক্তভোগী। পৃথিবীব্যাপি তাপমাত্রা বেড়ে যাওয়ার ফলে পর্বতচূড়া, হিমবাহ ইত্যাদিতে জমানো বরফ গলে সমুদ্র পৃষ্ঠের উচ্চতা বৃদ্ধি পাচ্ছে, বৃদ্ধি পাচ্ছে প্রাকৃতিক দুর্যোগ ঘটার সম্ভাবনা ও এর ফলে ক্ষতির মাত্রা।

বাংলাদেশ জলবায়ু পরিবর্তনের ফলে সবচেয়ে বেশি ক্ষতিগ্রস্ত হচ্ছে। অনিয়মিত বৃষ্টিপাত, অতিবৃষ্টি, অনাবৃষ্টি ও অসময়ে বৃষ্টি, খরা, লবণাক্ততা সমস্যা, নদী ভাঙ্গন, নদীর গতিপথ পরিবর্তন– এ সবগুলো দিকই আমাদের উদ্বেগের বিষয় হয়ে দাঁড়িয়েছে। মরুকরণ, ভূগর্ভস্থ পানির স্তর হ্রাস, সুপেয় পানির অভাব, প্রাকৃতিক সম্পদ হ্রাস, কৃষিভিত্তিক উৎপাদন হ্রাস ও ধ্বংস, খাদ্য সংকট, জীবনোপকরণ হ্রাস, জীবিকার উৎস ধ্বংস ইত্যাদি এদেশের দরিদ্র জনগোষ্ঠিকে আরো বেশি অপুষ্টি ও স্বাস্থ্য ঝুঁকির দিকে ঠেলে দিচ্ছে।

জলবায়ু পরিবর্তনের ঝুঁকি মোকাবেলায় বাংলাদেশ সরকার ইতোমধ্যে বেশ কিছু পদক্ষেপ গ্রহন করেছে। সরকারের নিজস্ব অর্থে 'জলবায়ু ট্রাস্ট ফাণ্ড' গঠন এর অন্যতম। জলবায়ু পরিবর্তনে অন্যান্য বিষয়গুলোর সাথে স্বাস্থ্য ঝুঁকি ওতপ্রোতভাবে জড়িত। স্বাস্থ্য ঝুঁকি মোকাবিলার জন্য জলবায়ু ট্রাস্ট ফাণ্ডের আওতায় স্বাস্থ্য ও পরিবার কল্যাণ মন্ত্রণালয় কর্তৃক একটি প্রকল্প বাস্তবায়িত হচ্ছে পরিবেশ ও বন মন্ত্রণালয়ের সহযোগিতায়।

জলবায়ু পরিবর্তনের ফলে স্বাস্থ্যখাতে উদ্ভূত ঝুঁকিগুলো মোকাবেলায় আমাদের সবাইকে সম্মিলিতভাবে এখন থেকেই প্রচেষ্টা চালিয়ে যেতে হবে। আমার দৃঢ় বিশ্বাস, পরিবেশ ও বন মন্ত্রণালয়ের "জলবায়ু পরিবর্তন ট্রাস্ট" ফাণ্ডের আওতায় স্বাস্থ্য ও পরিবার কল্যাণ মন্ত্রণালয়ের প্রকল্পের অধীনে জাতীয় শিক্ষাক্রম ও পাঠ্যপুস্তক বোর্ডের সহযোগিতায় স্কুল শিক্ষার্থী ও শিক্ষকদের জন্য 'জলবায়ু পরিবর্তন ও স্বাস্থ্য সুরক্ষা' নামক সহায়িকা দু'টো জলবায়ু পরিবর্তনের ক্ষতিকর দিকগুলো মোকাবেলা এবং স্বাস্থ্য সুরক্ষায় কার্যকর ভূমিকা গ্রহণে আমাদের সকলের সচেতনতা বৃদ্ধিতে সহায়তা করবে।

সহায়িকা তৈরিতে যারা কার্যকর ভূমিকা রেখেছেন তাঁদের সকলকে অভিনন্দন।

জয় বাংলা

জয় বঙ্গবন্ধু

ড. হাছান মাহমুদ এম.পি
প্রতি মন্ত্রী
পরিবেশ ও বন মন্ত্রণালয়

CCHPU
Climate Change & Health Promotion Unit

মুখবন্ধ

পৃথিবীতে জলবায়ু পরিবর্তিত হচ্ছে– বিষয়টি এখন সর্বজন স্বীকৃত। জলবায়ু পরিবর্তনের প্রভাবে বিশ্বের সবচেয়ে ক্ষতিগ্রস্ত দেশগুলোর মধ্যে বাংলাদেশ অন্যতম। জলবায়ু পরিবর্তনজনিত কারণে বাংলাদেশের দক্ষিণাঞ্চল, উত্তরাঞ্চল, মধ্যাঞ্চল, দক্ষিণ-পশ্চিমাঞ্চলসহ দেশের সমগ্র উপকুলীয় অঞ্চলের মানুষ আজ চরম বিপদাপন্ন। বাংলাদেশের ভৌগলিক অবস্থান, জনসংখ্যার ঘনত্ব, আর্থসামাজিক অবকাঠামো এবং প্রাকৃতিক সম্পদের উপর নির্ভরশীলতা এদেশের মানুষকে আরও ঝুঁকির সম্মুক্ষীন করে তুলছে। তাই প্রাকৃতিক দুর্যোগে বাংলাদেশকে দিতে হচ্ছে চরম মূল্য। জলবায়ু পরিবর্তনের প্রভাবে প্রতি বছরই বাংলাদেশে প্রাকৃতিক দুর্যোগের প্রকোপ বৃদ্ধি পাচ্ছে। জলবায়ু পরিবর্তনের সঙ্গে বাস্তুতন্ত্রের (ইকোসিস্টেম) বিনাশ এবং বিভিন্ন প্রজ্যাতির বিলুপ্তি ঘটছে- এসবই সত্য, কিন্তু স্বাস্থ্যের উপর এর প্রভাব আরও প্রত্যক্ষ। মানুষের দ্বারা জলবায়ুতে যে পরিবর্তন হচ্ছে তার ফলে আমাদের স্বাস্থ্য অনেকভাবে ক্ষতিগ্রস্ত হচ্ছে । জলবায়ু পরিবর্তনজনিত স্বাস্থ্য ঝুঁকি ও করণীয় সম্পর্কে জনগণের বিশেষ করে শিক্ষার্থীদের জানা প্রয়োজন, কারণ ভবিষ্যতে দেশ গড়ার দায়িত্ব তাদেরকেই পালন করতে হবে।

মানুষের স্বাস্থ্যের সাথে জলবায়ু পরিবর্তনের যে যোগসূত্র আছে সে সম্বন্ধে স্কুলের ছাত্রছাত্রীদের সচেতন করার লক্ষ্য নিয়ে এই সহায়িকা প্রণয়ন করা হলো। যে পরিবর্তনগুলো ঘটছে তার কারণ কী, কে কীভাবে আমাদের প্রভাবিত করছে, বর্তমান এবং ভবিষ্যৎ প্রভাব কী এবং জলবায়ু পরিবর্তনে স্বাস্থ্য সুরক্ষার জন্য করণীয়, সে সব বিষয় শিক্ষার্থীদের জানা প্রয়োজন। এই সহায়িকাটি শিক্ষার্থী ও শিক্ষকদের জন্য আলাদাভাবে তৈরি। এই সহায়িকাটি বিনামূল্যে সহপাঠ হিসাবে বিতরণ করা হবে। সহপাঠটি শিক্ষার্থীদের জলবায়ু পরিবর্তন ও স্বাস্থ্য ঝুঁকি মোকাবেলায় বিশেষভাবে সহায়তা করবে বলে আমি মনে করি।

প্রফেসর মোঃ মোস্তফা কামালউদ্দিন
চেয়ারম্যান
জাতীয় শিক্ষাক্রম ও পাঠ্যপুস্তক বোর্ড
ঢাকা

সূচিপত্র

বিষয়		পৃষ্ঠা নং
অধ্যায় - ১		
	**জলবায়ু পরিবর্তন**	১-৭
	জলবায়ু পরিবর্তন কী, সাইক্লোন	১
	বন্যা, বৃষ্টিপাত	২
	খরা, বিশ্বব্যাপী উষ্ণতা বৃদ্ধি, প্রাকৃতিক বিপর্যয়, গ্রিনহাউস প্রভাব	৩
	মানুষ কীভাবে গ্রিনহাউস গ্যাস তৈরি করে	৫
	জয়বায়ু পরিবর্তন বিষয়ক আন্তর্জাতিক উদ্যোগ	৭
অধ্যায় - ২		
	**জলবায়ু পরিবর্তন ও স্বাস্থ্যসমস্যা**	৯-১৪
	হিট স্ট্রোক, শ্বাসপ্রশ্বাস সংক্রান্ত রোগ	৯
	জলাবদ্ধতা ও চর্মরোগ, আঘাত বা ক্ষত, পানিবাহিত রোগ	১০
	কীটপতঙ্গ (ভেক্টর) বাহিত রোগ, ম্যালেরিয়া, ডেঙ্গু জ্বর	
	জাপানিজ এনকেফলাইটিস	১১
	খাদ্য সমস্যা, পুষ্টির অভাব	১২
	মনোসামাজিক পরিচর্যা	১৩
	জলবায়ুর পরিবর্তন ও মনুষ্য স্বাস্থ্যের যোগসূত্র	১৪
অধ্যায় - ৩		
	**জলবায়ু পরিবর্তনজনিত স্বাস্থ্য ঝুঁকি মোকাবেলা**	১৫-২০
	জলবায়ু পরিবর্তনজনিত ঝুকি হ্রাস বা মেটিকেশনের উপায়	১৫
	দৈনন্দিন জীবনে যে কাজগুলো আমরা সহজেই করতে পারি	
	পানিবাহিত রোগ প্রতিরোধ, কীটপতঙ্গ বাহিত রোগ হতে রক্ষা	
	সবুজ থাকো, কাগজ বাচাঁও, নবায়নের শক্তি ব্যবহার	১৬
	রিসাইকেল, রিডিউস, রিইউজ	১৭
	অ্যাডাপ্টেশন বা অভিযোজন	১৮
অধ্যায় - ৪		
	**প্রাকৃতিক সম্পদ সংরক্ষণ**	২১-২২
	সুন্দরবন	২১
	ভৌগলিক গঠন, জীববৈচিত্র্য	
	কক্সবাজার	২২
অধ্যায় - ৫		
	**জলবায়ু পরিবর্তন ও স্বাস্থ্য সুরক্ষা**	২৩-২৪
	কেস স্টাডি-১, কেস স্টাডি-২, কেস স্টাডি-৩	২৩
	পুনরালোচনা	
	ক্লাইমেট চেঞ্জ এণ্ড হেল্‌থ প্রমোশন ইউনিট	২৪
অধ্যায় - ৬		
	**দূষণ কমাই সুস্থ থাকি**	২৫-২৬
	বায়ু দূষণ, মাটি দূষণ, নদী দূষণ, পানি দূষণ	২৫
	শব্দ দূষণ, পৃথিবীতে পানি	২৬
অধ্যায় - ৭		
	**শিক্ষার্থীদের জন্য ৩ দিনের পাঠ পরিকল্পনা**	২৭-৩৫
	ক্রিয়াকলাপ প্রথম দিন	২৭
	ক্রিয়াকলাপ দ্বিতীয় দিন	৩৩
	ক্রিয়াকলাপ তৃতীয় দিন	৩৪
	শব্দকোষ	৩৬
	মানুষ দ্বারা জলবায়ু পরিবর্তন, বায়ুপরিমণ্ডল, বায়োফুয়েল	
	কার্বন ডাইঅক্সাইড, কার্বন ফুটপ্রিন্ট, জলবায়ু পরিবর্তন, কার্বন সিঙ্ক	
	গ্রিনহাউস গ্যাস, নাপা, ইউএনএফসিসি	

অধ্যায় – ১

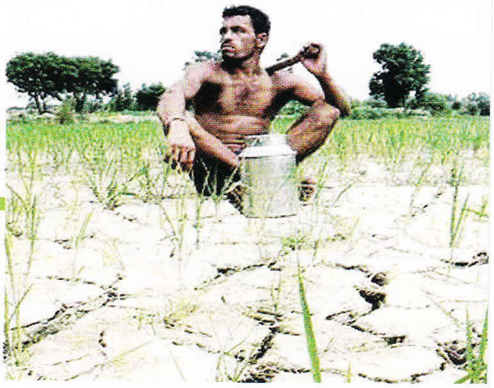

জলবায়ু পরিবর্তন

জলবায়ু পরিবর্তন কী ?

পৃথিবী সৌরজগতের একটি গ্রহ। পৃথিবীতে মানুষ বেড়ে চলেছে। পরিবেশ প্রতিনিয়তই পরিবর্তনশীল। আমাদের বেঁচে থাকার জন্য পরিবেশের ভারসাম্য বজায় রাখা খুবই জরুরী। আমাদের পৃথিবীটাকে ঘিরে রয়েছে বায়ুমন্ডল। আমরা যখন বায়ুমন্ডলের কথা বলি তখন আবহাওয়া আর জলবায়ুর বিষয়টি সামনে আসে। সূর্যের তাপমাত্রা, আকাশ মেঘলা না কি রোদ ঝলমলে, বাতাসে জলীয় বাষ্পের পরিমাণ কেমন অর্থাৎ বাতাস ভেজা না শুকনা, বাতাসের গতি এই সব কিছু মিলে পরিবেশের প্রতিদিনের যে অবস্থা তাকে বলে আবহাওয়া। যেমন খুব ঠান্ডায় আমরা বলি আজকের আবহাওয়াটা খুব ঠান্ডা। ঠিক একইভাবে জলবায়ু হচ্ছে কোনো এলাকার কমপক্ষে ৩০ বছরের গড় আবহাওয়া। জলবায়ুর পরিবর্তন একটি নিয়মিত প্রাকৃতিক ঘটনা। জলবায়ু পরিবর্তনের ফলে পৃথিবীর তাপমাত্রা বৃদ্ধি পাচ্ছে। ইতোমধ্যে অনেক দেশ এবং জনগণ জলবায়ু পরিবর্তনজনিত প্রাকৃতিক দুর্যোগের প্রভাবে দুর্ভোগ পোহাচ্ছে। বৈশ্বিক উষ্ণায়ন বাংলাদেশসহ সারা বিশ্বের আবহাওয়ার ধরন এবং ঋতু বৈচিত্র্য পাল্টে দিচ্ছে। এর ফলে প্রাকৃতিক দূর্যোগ– যেমন, অনাবৃষ্টি, অতিবৃষ্টি, বন্যা, ঘূর্ণিঝড়, সামুদ্রিক জলোচ্ছ্বাস ইত্যাদি ঘন ঘন দেখা দিচ্ছে, সে সঙ্গে ক্ষতির পরিমাণ বেড়ে যাচ্ছে। এইসব দুর্যোগে শত শত মানুষের মৃত্যু ঘটছে এবং কোটি কোটি টাকার সম্পদহানি হচ্ছে যার প্রভাব পড়ছে লাখ লাখ মানুষের জীবিকার উপর। এর মধ্যে নতুন করে ভূমিধ্বস ও ভূমিকম্পের প্রবণতা যোগ হয়েছে। আজকাল 'জলবায়ু পরিবর্তন' কথাটি জলবায়ুর নানা পরিবর্তিত পরিস্থিতি বোঝাতে ব্যবহার করা হয়, যার শুরু হয়েছে উনবিংশ শতাব্দীর মাঝামাঝি সময় থেকে। জলবায়ু পরিবর্তনে বাংলাদেশ সবচেয়ে বেশি ক্ষতিগ্রস্ত হচ্ছে।

সাইক্লোন

সাইক্লোন সবচেয়ে প্রলয়ঙ্করী দুর্যোগ। ১৯৭০ সাল থেকে ১৯৯৮ সালের মধ্যে বাংলাদেশে মোট ১৩ টি বড় আকারের সাইক্লোন আঘাত হানে, তার মধ্যে ১৯৭০ সালে ঘটে যাওয়া সাইক্লোনে প্রায় ৩ লক্ষ মানুষের প্রাণহানি হয়, হাজার হাজার লোক গৃহহারা হয়, ফসলি অনেক জমি নষ্ট হয়; কোটি কোটি টাকার সম্পদহানি হয়।

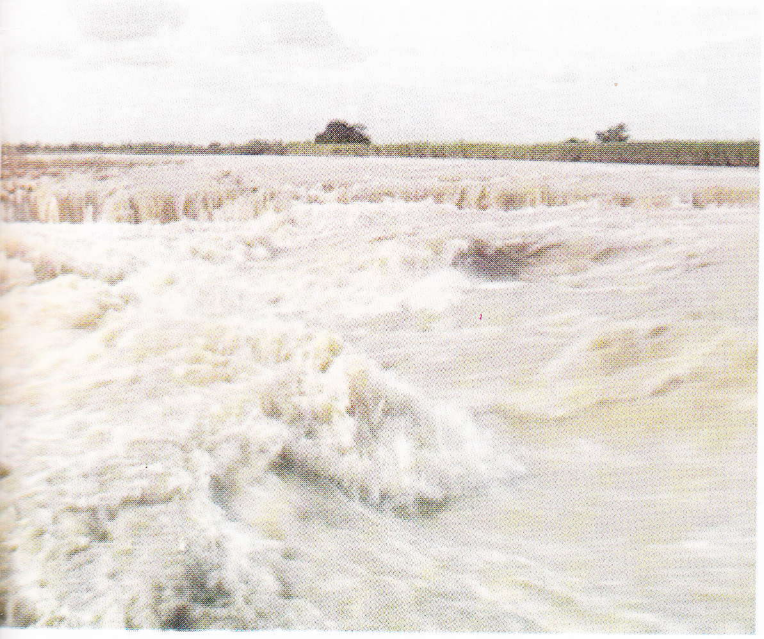

উপকূল অঞ্চলে জলস্তর বৃদ্ধি

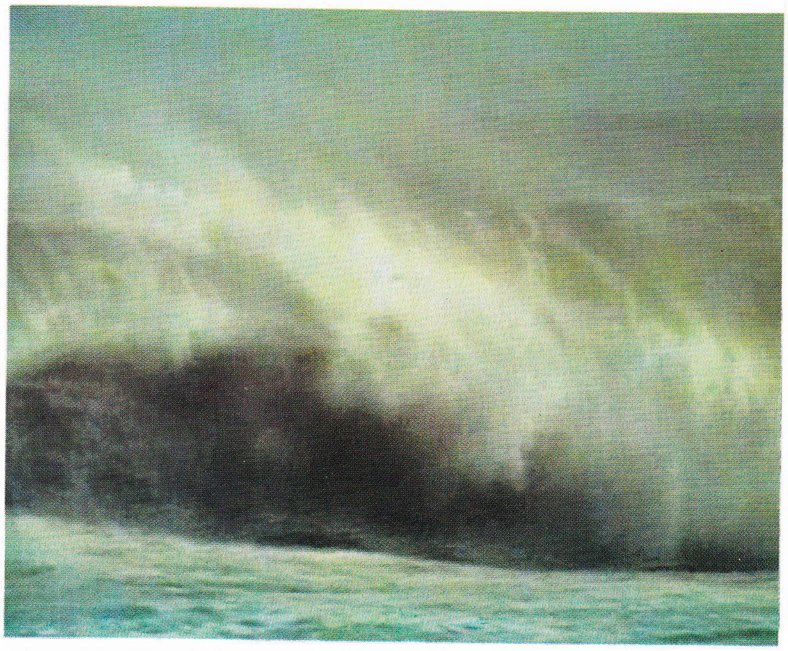

সাইক্লোন

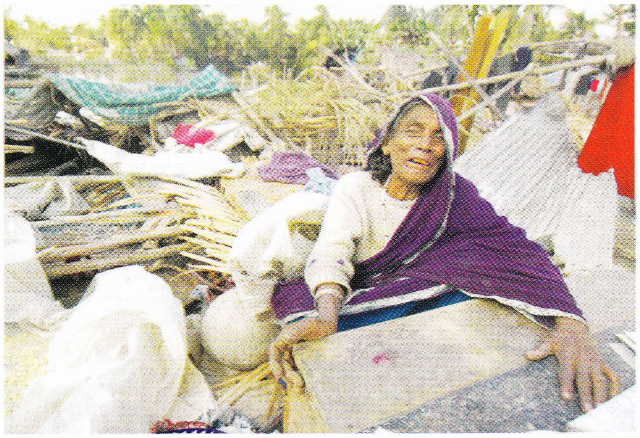

ঘূর্ণিঝড় 'সিডরে' বাড়ি ঘর সহায় সম্পদ হারিয়ে এক বৃদ্ধার আহাজারি
ছবি : জিয়া ইসলাম

- ১৯৯১ সালের ২৯ শে এপ্রিলের প্রলয়ঙ্করী ঘূর্ণিঝড়ে প্রায় ১৪ লক্ষ মানুষ প্রাণ হারায় এবং কয়েক হাজার একর ফসলি জমি নষ্ট হয়।
- ২০০৭ সালের ১৫ই নভেম্বর ঘূর্ণিঝড় 'সিডর'-এ ৩০টি জেলার প্রায় ৩৫০০ জনের প্রাণহানি হয়, ১৫ লক্ষের বেশি ঘরবাড়ি নষ্ট হয় এবং ফসলি জমির ব্যাপক ক্ষতিসাধন হয়।
- সিডরে দেশের দক্ষিণাঞ্চলের ৯টি জেলা বিশেষভাবে ক্ষতিগ্রস্ত হয়। বিশুদ্ধ পানীয়জল, স্যানিটেশন ব্যবস্থার অভাব মানুষের স্বাস্থ্যের বিষয়টি ঝুঁকির দিকে ঠেলে দেয়।
- ২০০৯ সালে ঘূর্ণিঝড় 'আইলা' আঘাত হানে, যা ১১ টি জেলার উপর দিয়ে প্রবাহিত হয়। প্রায় ৪০ লক্ষ লোক ক্ষতিগ্রস্ত হয়। উপকূলীয় অঞ্চলে লবণাক্ততা বৃদ্ধি পেয়েছে।

বন্যা

জলবায়ু পরিবর্তনের কারণে বন্যার মাত্রা বাড়ছে। জলবায়ু পরিবর্তনের প্রভাবে তাপমাত্রা ও বৃষ্টিপাতের পরিমাণ ও ধরন পরিবর্তিত হচ্ছে। এর ফলে মানুষ নানা ধরনের রোগ ব্যাধিতে আক্রান্ত হচ্ছে, কর্মশক্তি কমছে এবং উৎপাদন ব্যাহত হচ্ছে।

- ১৯৮৮ সালের বন্যায় ৫২টি জেলার প্রায় ৮৯,০০০ বর্গকিলোমিটার এলাকা প্লাবিত হয়। ১৯৯৮ সালের বন্যার স্থায়ীত্ব ছিল প্রায় ২ মাস। এতে ৫৩ টি জেলার ১০,০০০ বর্গকিলোমিটার এলাকা ক্ষতিগ্রস্ত হয়।
- ২০০৪ সালের বন্যায় ৪০ টি জেলা ক্ষতিগ্রস্ত হয়, এতে মৃতের সংখ্যা ছিল ৭৪১ জন।
- ২০০৭ সালের বন্যায় ৯৭০ জনের মৃত্যু হয়। এ সময় শুধুমাত্র ডায়রিয়ায় আক্রান্ত হয় ২ লক্ষেরও বেশি মানুষ। বন্যা পরবর্তী সময়ে সাপের কামড়ে মারা যায় ১১৬ জন।

বৃষ্টিপাত

বিগত কয়েক দশক ধরে দেখা যাচ্ছে বৃষ্টিপাতের ধারায় ব্যাপক পরিবর্তন লক্ষ্য করা যাচ্ছে। বর্ষাকালেও সময়মত বৃষ্টিপাত হচ্ছে না। সামগ্রিক বৃষ্টিপাতের পরিমাণ পূর্বের তুলনায় কমে গেছে। যদিও হঠাৎ করে অতিবৃষ্টি জন দুর্ভোগ বাড়িয়ে তুলছে। যেমন ২০০৯ সালের ২৭ শে জুলাই ঢাকায় মাত্র ৬ ঘন্টার ব্যবধানে রাতের বেলা ২৯০ মিমি এবং ২৪ ঘন্টায় ৩৬০ মিমি বৃষ্টিপাত হয়। এত অল্প সময়ে এই বৃষ্টিপাত আগের সমস্ত রেকর্ড অতিক্রম করে।

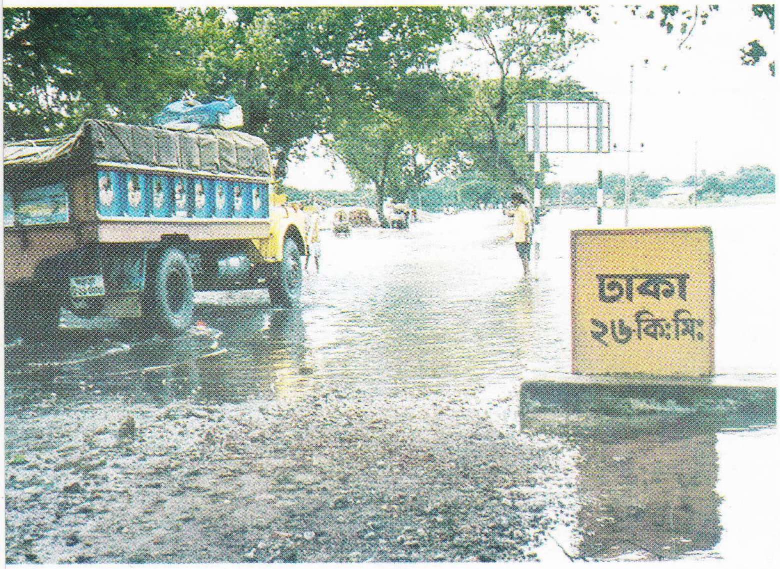

১৯৯৮-এর বন্যায় অচল মহাসড়ক *ছবি : জাহাঙ্গীর সেলিম*

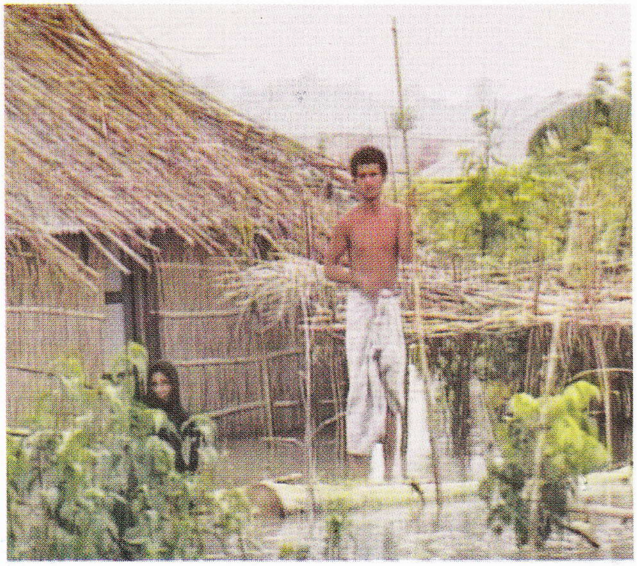

বন্যা ২০০৪

অনিয়মিত, অপর্যাপ্ত ও অল্প সময়ে অতি বৃষ্টি কৃষি সহায়ক না হবার কারণে খাদ্য নিরাপত্তা হুমকীর মুখে।

খরা

সময়মত বৃষ্টিপাতের অভাবে দেশে খরা দীর্ঘমেয়াদী হচ্ছে। দেশের উত্তরাঞ্চলসহ কয়েকটি এলাকা খরা প্রবণ এলাকা হিসেবে চিহ্নিত করা হয়েছে। নদী, খাল, বিল, পুকুর, নালা, ডোবা প্রকৃতিক জলাশয় ইত্যাদি শুকিয়ে যাচ্ছে। কৃষি কাজে ভূগর্ভস্থ পানির অতিরিক্ত ব্যবহারের কারণে দেশের অনেক স্থানে পানির স্তর ক্রমশই নীচে নেমে যাচ্ছে। এ কারণে ভবিষ্যতে চাষাবাদও হুমকীর সম্মুখীন হবার সম্ভাবনা রয়েছে।

বিশ্বব্যাপী উষ্ণতা বৃদ্ধির প্রভাব আমাদের সকলের উপর পড়ছে

আমাদের প্রথমেই বুঝতে হবে যে আবহাওয়া এবং জলবায়ু দুইটি আলাদা জিনিস।

ভূমণ্ডলে জলবায়ু সবসময়ই পরিবর্তনশীল। অতীতে প্রাকৃতিক কারণে জলবায়ুর পরিবর্তন ঘটলেও বর্তমানে মানুষের অদূরদর্শিতার ফলে এই পরিবর্তনের মাত্রা অনেক বৃদ্ধি পেয়েছে। জলবায়ু পরিবর্তনের মাত্রা বৃদ্ধির কারণে দুর্যোগ প্রবণতাও বাড়ছে।

প্রাকৃতিক বিপর্যয়ের প্রবণতা

সাম্প্রতিক সময়ের তথ্য উপাত্ত বিশ্লেষণে দেখা যায় প্রাকৃতিক বিপর্যয়গুলোর মধ্যে ভূমিকম্পের তুলনায় ঝড়, ঘুর্ণিঝড়, বন্যা, জলোচ্ছ্বাস, প্লাবন ইত্যাদি খবর বেড়েই চলেছে। তাই বিশ্বব্যাপী উষ্ণতা বৃদ্ধি কীভাবে প্রাকৃতিক বিপর্যয়কে প্রভাবিত করছে বিষয়টি আমাদের সকলেরই জানা প্রয়োজন।

জলবায়ু পরিবর্তনের প্রধান কারণ হলো 'গ্রিনহাউস প্রভাব' যার দ্বারা পৃথিবীর বায়ুমণ্ডল সূর্যের শক্তিকে ধরে রাখে ঠিক গ্রিনহাউসের মতই। প্রাকৃতিক 'গ্রিনহাউস প্রভাব' গ্রহকে উষ্ণ রেখে প্রাণবন্ত রাখতে সাহায্য করে, কিন্তু বর্তমানে এ সামঞ্জস্য অতিমাত্রায় ব্যাহত হচ্ছে।

- আবহাওয়া হচ্ছে প্রাকৃতিক অবস্থা যেখানে রোদ,বৃষ্টি,ঝড়,পানি প্রতিনিয়ত অথবা ঘণ্টায় ঘণ্টায় বদলে যাচ্ছে।
- জলবায়ু হচ্ছে আবহাওয়ার সমষ্টিগত গড়, যার স্থিতি সময় সাপেক্ষ যা এক বছর অথবা বেশ কয়েক বছর ধরে চলে।

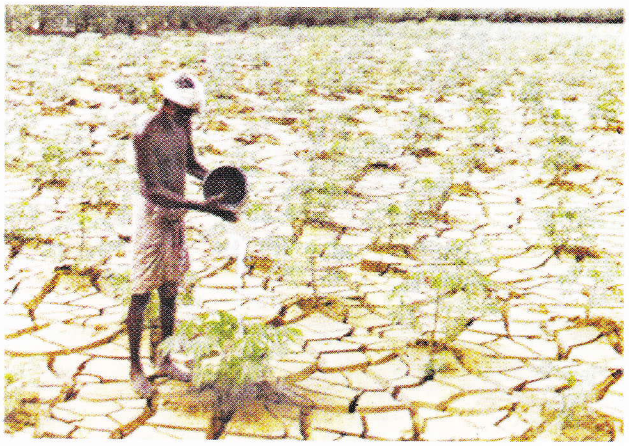

খরায় কৃষকের ফসল বাঁচানোর প্রাণান্তকর প্রচেষ্টা

গ্রিনহাউস প্রভাব কী?

গ্রিনহাউস কাকে বলে একথা সবার জানা। শীতপ্রধান দেশে ঠাণ্ডা থেকে বাঁচানোর জন্য ঘরের ভিতরে গাছপালা লাগানো হয়। এ ঘরগুলো সাধারণত কাঁচের তৈরি। এর ফলে সূর্যের আলো ঘরের ভিতরে ঢুকতে পারে। ঘরের ভিতরে গাছপালা সূর্যের আলোতে সালোকসংশ্লেষণের মাধ্যমে শর্করাজাতীয় খাদ্য তৈরি করে বেঁচে থাকতে পারে। সূর্যের আলোতে ঘরের পরিবেশ গরম থাকে। কাচ তাপ কুপরিবাহী বলে বাইরের ঠাণ্ডা ভিতরে ঢুকতে পারে না, ভিতরের গরমও বাইরে বের হতে পারে না। পৃথিবীর বায়ুমণ্ডলের কার্বন ডাইঅক্সাইড অনেকটা গ্রিনহাউস কাচের মতো কাজ করে। সূর্যের আলো পৃথিবীকে উত্তপ্ত করে। উত্তাপের অনেকটা বিকিরিত হয়ে বায়ুমণ্ডলের মধ্যে দিয়ে হারিয়ে যায় মহাশূন্যে। এর ফলে পৃথিবীর বায়ুমণ্ডলের উত্তাপ মোটামুটি একরকম থাকে। কিন্তু বায়ুমণ্ডলে কার্বন ডাইঅক্সাইডের পরিমাণ বেড়ে গেলে অবস্থা কিন্তু এ রকম থাকে না।

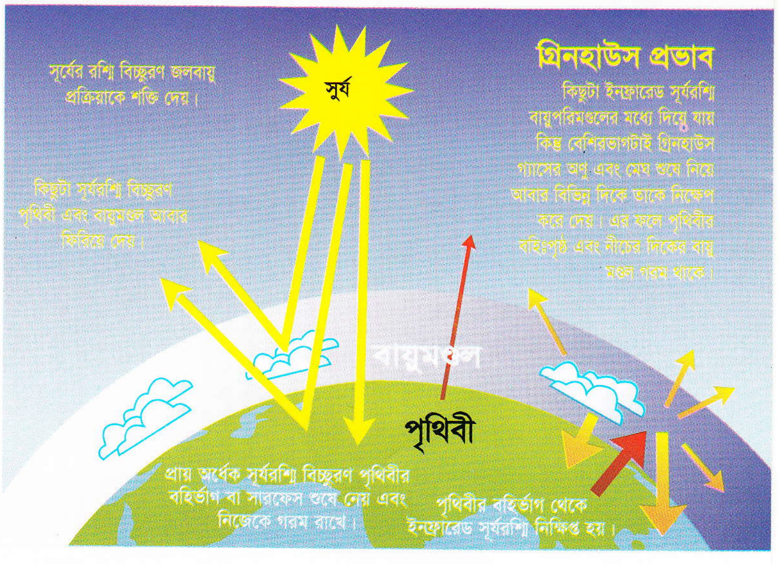

১৮৭০ থেকে ১৯৯০ সালের মধ্যে বায়ুমণ্ডলে CO_2-র বৃদ্ধি

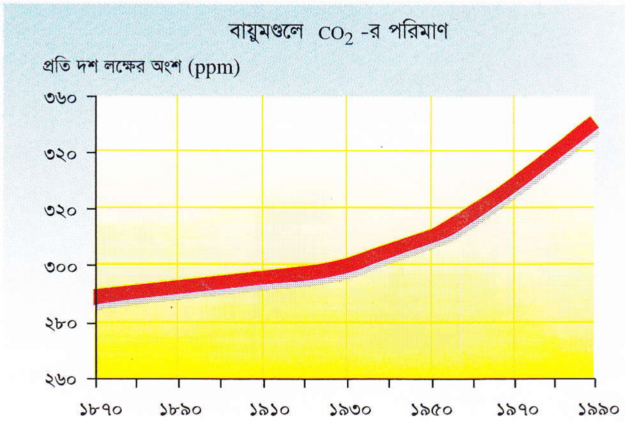

কার্বন ডাইঅক্সাইড পৃথিবী থেকে বিকিরিত তাপের খানিকটা ধরে রাখে। এতে বায়ুমণ্ডলের তাপমাত্রা বেড়ে যায়। বিজ্ঞানীরা হিসাব করে দেখেছেন যে বায়ুমণ্ডলে কার্বন ডাইঅক্সাইডের পরিমাণ দিন দিন বেড়েই চলেছে। কার্বন ডাইঅক্সাইডের পরিমাণ কেন বাড়ছে তোমরা নিশ্চয়ই জান।

ব্যাপকহারে গাছপালা কমে যাওয়া এর একটি প্রধান কারণ। জীবাশ্ম জ্বালানির ব্যবহার আর একটি প্রধান কারণ। পেট্রোল, ডিজেল, কেরোসিন, কয়লা এগুলো জীবাশ্ম জ্বালানি। কলকারখানা, যানবাহন এবং গৃহস্থালির কাজে এসবের ব্যবহার দিন দিন বাড়ছে। এর ফলে বায়ুমণ্ডলে কার্বন ডাইঅক্সাইডের পরিমাণও দিন দিন বাড়ছে।

মাথাপিছু জাতীয় কার্বন ডাইঅক্সাইড (CO_2) নির্গমন :

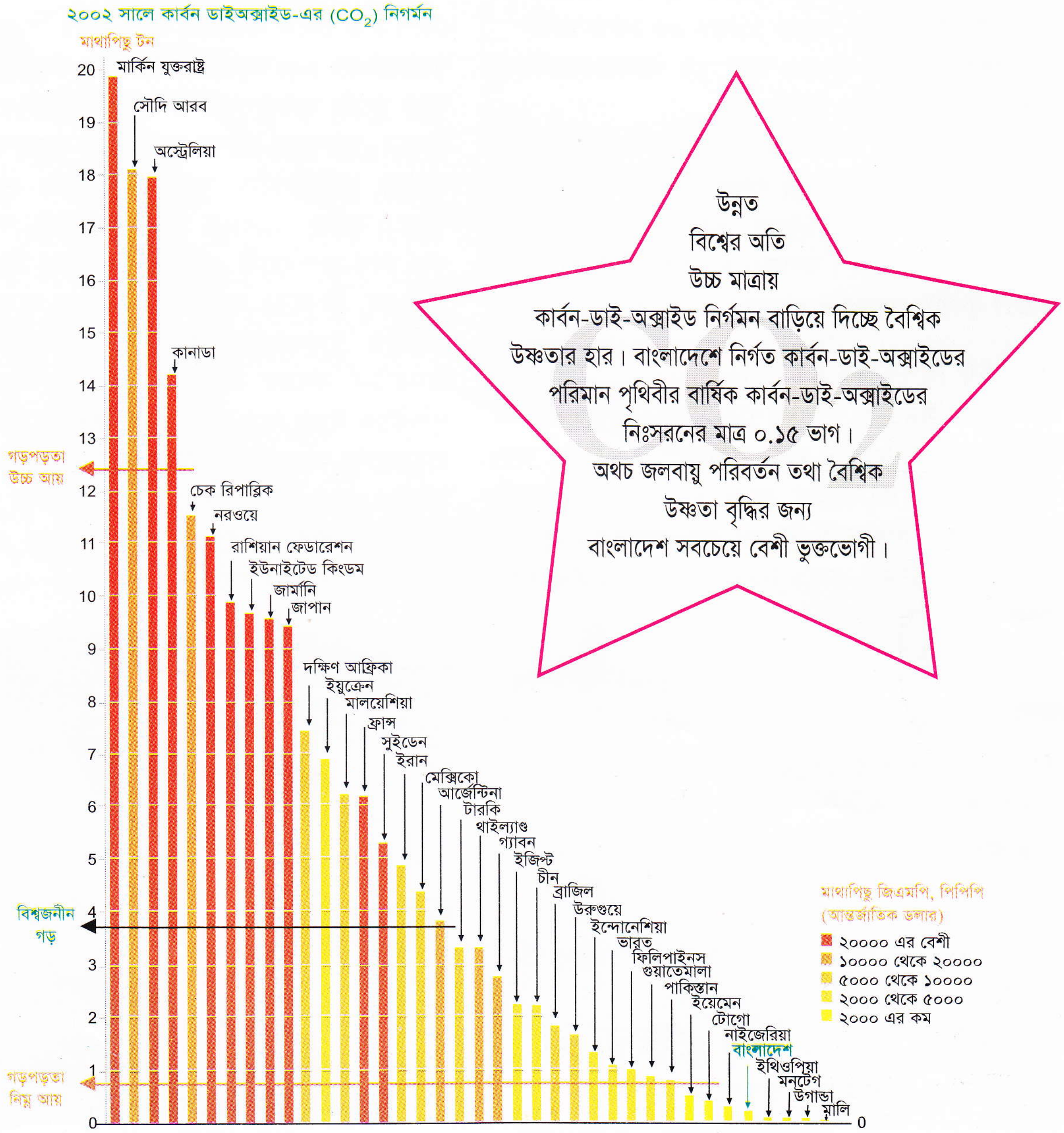

উন্নত
বিশ্বের অতি
উচ্চ মাত্রায়
কার্বন-ডাই-অক্সাইড নির্গমন বাড়িয়ে দিচ্ছে বৈশ্বিক
উষ্ণতার হার। বাংলাদেশে নির্গত কার্বন-ডাই-অক্সাইডের
পরিমান পৃথিবীর বার্ষিক কার্বন-ডাই-অক্সাইডের
নিঃসরনের মাত্র ০.১৫ ভাগ।
অথচ জলবায়ু পরিবর্তন তথা বৈশ্বিক
উষ্ণতা বৃদ্ধির জন্য
বাংলাদেশ সবচেয়ে বেশী ভুক্তভোগী।

গ্রিনহাউস প্রতিক্রিয়ার কারণে বায়ুমণ্ডলের উত্তাপ বেড়ে গেলে পরিবেশের উপর এর প্রতিক্রিয়া কী হবে বলতে পার ? তোমরা জান পৃথিবীর দুই মেরু অঞ্চলে বিপুল পরিমাণ বরফ জমে আছে। গ্রিনহাউস প্রতিক্রিয়ার কারণে বায়ুমণ্ডলের উত্তাপ বাড়তে থাকলে এসব বরফ বেশি করে গলতে শুরু করবে। এর ফলে সমুদ্রের পানির উচ্চতা বাড়তে থাকবে। সমুদ্র তীরের অনেক শহর, দ্বীপ ও দেশ ধীরে ধীরে পানিতে ডুবে যাবে। বাংলাদেশের অনেক অঞ্চল বঙ্গোপসাগরের পানিতে তলিয়ে যাবে।

দশ হাজার বছরেরও বেশি সময় ধরে পৃথিবীর তাপমাত্রা আনুপাতিকভাবে স্থির ছিল। কিন্তু বৈজ্ঞানিকরা লক্ষ্য করেছেন যে আমাদের পৃথিবী খুব দ্রুত উষ্ণ হয়ে যাচ্ছে। এই অবস্থাকে বিশ্ব উষ্ণায়ন (Global Warming) বলা হয়। বায়ুমণ্ডলে গ্রিনহাউস গ্যাসের পরিমাণ বেড়ে যাওয়ায় গ্রীণহাউস প্রভাব আরও জোরদার হয়েছে এই পরিবর্তন মানুষের দৈনন্দিন সামাজিক কার্যকলাপের ফলে হয়েছে যা জি এইচ জি বা গ্রিনহাউস গ্যাস-এর পরিমাণ বাড়িয়ে দিয়েছে।

উষ্ণতা বৃদ্ধির পরীক্ষা

এই সহজ পরীক্ষাটি শিক্ষার্থীরা বাড়িতে করতে পারে। দুইটি বোতলে এক চামচ করে পানি নিতে হবে। একটি বোতল ঢেকে দিতে হবে, অন্যটি ঢাকনা না লাগিয়ে বোতল দুইটি রোদে রাখো। কয়েক ঘন্টা পরে বোতলগুলো পরীক্ষা করে দেখো, খোলা বোতলটিতে কোন পরিবর্তন হয়নি, কিন্তু ঢাকনা দেওয়া বোতলটি বাষ্পীভূত এবং ভিতরটা গরম। এর কারণ কী? সূর্যের তাপ বন্ধ বোতল থেকে বের হতে পারেনি। সূর্যের উত্তাপে পানি বাষ্পীভূত হয়ে বোতলের মাঝেই আটকা পড়ে আছে, ঠিক যেমন করে গ্রীণহাউস উত্তাপ ফাঁদ তৈরি করে।

মানুষ কীভাবে গ্রিনহাউস গ্যাস তৈরি করে

কার্বন ডাইঅক্সাইড (CO_2) প্রধানত তৈরি হয়–জীবাশ্ম জ্বালানি ব্যবহার থেকে (যেমন কয়লা, তেল ও গ্যাস) অথবা বিদ্যুৎ উৎপাদন ও যানবাহন থেকে। তাছাড়া আবর্জনা যখন ভস্মীভূত করা হয় তখন প্রচুর পরিমাণে কার্বন ডাইঅক্সাইড বায়ুমণ্ডলে ছড়িয়ে পড়ে।

মিথেন (CH_4) প্রধানত গবাদি পশু (পরিপাক প্রক্রিয়া ও জমির সার), আবর্জনা শোধন প্রণালি (আবর্জনা পচিয়ে সার তৈরি), প্রাকৃতিক গ্যাস বিতরণ পাইপের মধ্যেকার ছিদ্র দিয়ে এবং অসম্পূর্ণ দহন প্রক্রিয়ার মাধ্যমে বায়ুমণ্ডলে ছড়ায়।

নাইট্রাস অক্সাইড (N_2O) অথবা লাফিং গ্যাস প্রধানত প্রস্তরীভূত কয়লা জ্বালানোর ফলে (বিশেষ করে পরিবহনের জন্য), রাসায়নিক কারখানায় এবং কৃষিকাজের বিভিন্ন প্রক্রিয়ার ফলে ছড়ায়।

ক্লোরোফ্লুরোকার্বনস (CFCs) ক্লোরোফ্লুরোকার্বনস (CFCs) এবং এসবের প্রতিকল্প বস্তু (HCFCs) মূলত হিমায়িত করার জন্য শীতাতপ নিয়ন্ত্রণযন্ত্র, রেফ্রিজারেটর, এ্যারোসোল এবং ফোম, প্লাস্টিক তৈরি করার জন্য ব্যবহৃত হয়।

ওজোন (O_3) ওজোন এক ধরনের গ্যাস। তিনটি অক্সিজেন পরমাণু মিলে ওজোনের একটি অণু তৈরি করে। ওজোনের রাসায়নিক সংকেত O_3। ট্রপোস্ফিয়ার, স্ট্রাটোস্ফিয়ার, আয়নোস্ফিয়ার-বায়ুমণ্ডলের এ স্তরগুলোর পরিচয় সবাই জানে। ভূপৃষ্ঠের ২৫ থেকে ৩০ কিলোমিটার উপরে বায়ুমণ্ডলের,স্ট্রাটোস্ফিয়ার অঞ্চলে ওজোন গ্যাসের একটি ঘন স্তর রয়েছে। ট্রপোস্ফিয়ার অঞ্চলেও ওজোন গ্যাস রয়েছে। ওজোন গ্যাসের এই স্তর পৃথিবীকে চার দিক থেকে ঢেকে রেখেছে। এই স্তরকে ওজোন ঢাল (Ozone Barrier) হিসেবে সবাই জানে। হাজার হাজার বছর ধরে ওজোনের এই আবরণ পৃথিবীর জীবজগতকে সূর্যের বিকিরিত মারাত্মক আলট্রাভায়োলেট রশ্মি থেকে রক্ষা করে আসছে।

আলট্রাভায়োলেট রশ্মি তিন ধরনের । দীর্ঘতরঙ্গ দৈর্ঘ্যের UV-A, মাঝারি তরঙ্গ দৈর্ঘ্যের UV-B এবং ক্ষুদ্র তরঙ্গ দৈর্ঘ্যের UV-C। স্ট্রাটোস্ফিয়ার অঞ্চলের ওজোন গ্যাসের আবরণ সবচেয়ে মারাত্মক আলট্রাভায়োলেট রশ্মি UV-C এর পুরো অংশ এবং স্বল্প ক্ষতিকর UV-B এর বেশির ভাগ অংশই আটকে রাখে। ট্রপোস্ফিয়ার অঞ্চলে ওজোন গ্যাসের আবরন এবং মেঘ UV-B রশ্মির অবশিষ্ট অংশকে ভূপৃষ্ঠে আসা থেকে বিরত রাখে। কিন্তু গ্রীণহাউস গ্যাস বৃদ্ধির ফলে এই ওজোন স্তরে ছিদ্র সৃষ্টি হচ্ছে। দৈনন্দিন জীবনে ব্যবহৃত রাসায়নিক যৌগ ক্লোরোফ্লোরো কার্বন CFC ওজোন স্তরের ক্ষতির জন্য দায়ী।

এ্যারোসলের টিনে এবং নানা রকমের ফোম তৈরিতে CFC যৌগ ব্যবহার করা হয়। বায়ুমণ্ডলে ছাড়া পেয়ে এগুলো উপরের স্তরে উঠে যায়। CFC যৌগগুলো বায়ুমণ্ডলের নিচের স্তরে সহজে ভাঙে না। স্ট্রাটোস্ফিয়ার অঞ্চলে আলট্রাভায়োলেট রশ্মি সংস্পর্শে এসে CFC ভেঙে ক্লোরিন ছেড়ে দেয়। মুক্ত ক্লোরিন পরমাণু তখন অনুঘটক হিসেবে কাজ করে ওজোন অণুকে ভেঙে একটি অক্সিজেন অণু এবং অক্সিজেন পরামাণু সৃষ্টি করে। এ প্রক্রিয়া যুগ যুগ ধরে চলতে থাকে। কার্বন টেট্রাক্লোরাইড, ট্রাইক্লোরোমিথেন এবং হেলোন নামে আরও কয়েকটি রাসায়নিক যৌগ ওজোন স্তরের ক্ষতি করে।

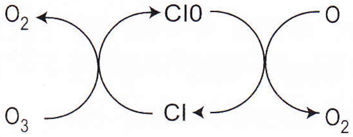

আলট্রাভায়োলেট রশ্মি জীবদেহের জন্য ক্ষতিকর। পরীক্ষায় প্রমাণিত হয়েছে যে, UV-A ও UV-B এর পরিমাণ বৃদ্ধি পেলে ত্বকের ক্যানসার হওয়ার ঘটনা বেড়ে যায়। এ ছাড়া UV-B এর কারণে নানা ধরনের চোখের অসুখ যেমন চোখে ছানি পড়া, চোখের লেন্সের বিকৃতি এবং বৃদ্ধদের মধ্যে চোখের দৃষ্টিহীনতা বেড়ে যায়। শরীরের রোগ প্রতিরোধ ক্ষমতা কমে যাওয়ার ফলে বিভিন্ন ধরনের সংক্রামক রোগের সংক্রমণের ঘটনা বেড়ে যায়।

কিছু কিছু গ্রিনহাউস গ্যাস স্বাভাবিকভাবে পৃথিবীর বায়ুমণ্ডলে তৈরি হয়। কিন্তু বৈজ্ঞানিকরা এই গ্যাস পরিমাপ করে দেখেছেন যে কয়েক যুগ ধরে এই গ্যাসের মাত্রা অনেকটা বেড়ে গেছে।

বিজ্ঞানীদের ধারণা বৈশ্বিক উষ্ণতা বৃদ্ধি হচ্ছে জলবায়ু পরিবর্তনের মূল নিয়ামক। বৈশ্বিক উষ্ণতা বৃদ্ধির ফলে পানি সম্পদ সংশ্লিষ্ট যে পরিবর্তনগুলো ঘটতে পারে (যার বেশ কিছু বর্তমানে পরিলক্ষিত হচ্ছে)ঃ

- অনিয়মিত বৃষ্টিপাত, অতিবৃষ্টি, অনাবৃষ্টি ও অসময়ে বৃষ্টি
- উত্তর মেরু ও দক্ষিণ মেরু অঞ্চলের জমাট বরফ গলে যাওয়া
- হিমালয় অঞ্চলের জমাট বরফ গলে যাওয়া, বন্যা
- বাষ্পীভবনের মাত্রাবৃদ্ধি
- খরা
- সমুদ্রপৃষ্ঠের উচ্চতা বৃদ্ধি
- পানির তাপমাত্রা বৃদ্ধি

- কার্বন ডাইঅক্সাইড (CO_2) মানুষের তৈরি সবচেয়ে বেশি (অ্যানথ্রোপোজেনিক) গ্রিনহাউস গ্যাস (জিএইচজি)। ১৯৭০ থেকে ২০০৪ সালের মধ্যে এর বার্ষিক নির্গমন প্রায় ৮০ শতাংশ বেড়েছে।
- বায়ুমণ্ডলে কার্বন ডাইঅক্সাইডের (CO_2) মাত্রা ২০০৬ সালে প্রতি দশ লাখে ৩৮৫ অংশ (অথবা পিপিএম) বেড়েছে, পৃথিবী সৃষ্টির পর গত ৬৫০,০০০ বছরে যার কোনো নজির নেই।
- বায়ুমণ্ডলে গ্রিনহাউস গ্যাস (জিএইচজি) বাড়ার প্রধান কারণ অতিমাত্রায় জীবাশ্ম জ্বালানি ব্যবহারের সঙ্গে সম্পর্কযুক্ত যেমন– কয়লা, গ্যাস ও তেল। এর সঙ্গে দাবানলও কিছুটা দায়ী।
- বৈজ্ঞানিকরা মনে করেন আগামী ৫০ বছরে CO_2-র মাত্রা আরও ৩০ শতাংশ বাড়বে।

১৯৫৭-২০০৭ সাল পর্যন্ত সারা বিশ্বের বায়ুমণ্ডলে CO_2-র বৃদ্ধি (পিপিএম)

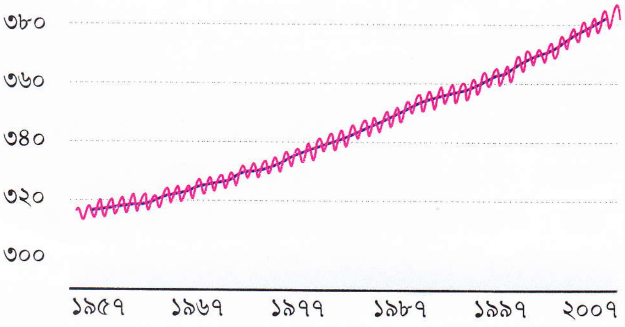

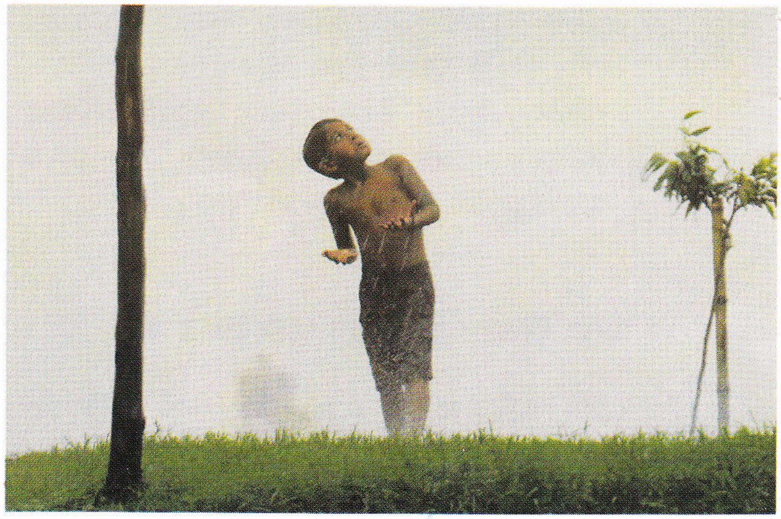

অনিয়মিত বৃষ্টি কিংবা অসময়ে অতিবৃষ্টি জলবায়ু পরিবর্তনের ইংগিত দেয়

ছবি : জিয়া ইসলাম

জলবায়ু পরিবর্তন বিষয়ক আন্তর্জাতিক উদ্যোগ

জলবায়ু পরিবর্তন সংক্রান্ত বৈজ্ঞানিক, কারিগরি ও আর্থ-সামাজিক পর্যবেক্ষণের জন্য জাতিসংঘের অধীনে ১৯৮৮ সালে ইন্টারগভার্নমেন্টাল প্যানেল অন ক্লাইমেট চেঞ্জ (আইপিসিসি) গঠিত হয়। জলবায়ু পরিবর্তনের ক্ষতিকর প্রভাবগুলো মোকাবেলায় ঝুঁকিহ্রাস (মিটিগেশন) ও অভিযোজন (অ্যাডাপটেশন) এর সম্ভাব্যতা ও বিকল্প পথগুলো যাচাই এর কিছু মূল্যায়ন প্রতিবেদন প্রকাশিত হয়। এরই ধারাবাহিকতায় ৫ম আইপিসিসি মূল্যায়ন প্রতিবেদন প্রকাশের লক্ষ্যে ২০১০ সালে জলবায়ু পরিবর্তনের–

বিশ্বব্যাপী উষ্ণায়নের তথ্য

১৮৫০-২০০৭ এর মধ্যে সমতলের কাছাকাছি তাপমাত্রার বিশ্বজনীন গড়

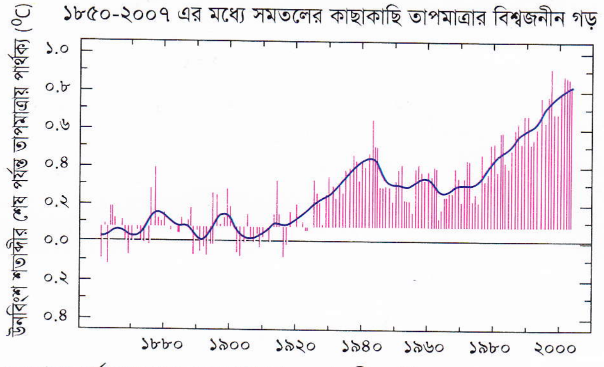

জলবায়ুর পূর্বাভাস এবং গবেষণার কাজে হ্যাডলী সেন্টার

- ভৌত বিজ্ঞান ভিত্তিক পর্যালোচনায় ২৫৮ জন বিশেষজ্ঞের সমন্বয়ে একটি দল গঠিত হয়;
- এর ক্ষতিকর প্রভাবগুলোর অভিযোজন কৌশল নির্ধারণে ৩০২ জন সদস্য নিয়ে অপর একটি বিশেষজ্ঞ দল গঠিত হয়।
- জলবায়ু পরিবর্তনের ঝুঁকিগুলো বৃহৎ আকারে পর্যালোচনা ও এটি হ্রাস করার কৌশল নির্ধারণ ও অবকাঠামো তৈরির বিষয়গুলো তুলে ধরার জন্য ২৭১ জনের তৃতীয় একটি বিশেষজ্ঞ দল গঠিত হয়।

- যেহেতু গ্রিনহাউস গ্যাস উষ্ণতাকে আটকে রাখে সেহেতু এই গ্যাসের বৃদ্ধির ফলে পৃথিবীর গড় তাপমাত্রা পরবর্তী ১০০ বছরে প্রতি দশকে ০.২ ডিগ্রি সেলসিয়াস করে বাড়িয়ে দেবে।
- গ্রিনহাউস গ্যাস (জিএইচজি) যেহেতু সব সময় বিদ্যমান, সে কারণে ২০০০ সাল পর্যন্ত যে হারে বৃদ্ধি পেয়েছে সে হারে যদি বৃদ্ধি পেতে থাকে তাহলেও প্রতি দশকে ০.১ ডিগ্রি সেলসিয়াস করে পৃথিবীর তাপমাত্রা এবং উষ্ণতা বাড়ত।

এই পঞ্চম মূল্যায়ন প্রতিবেদনটি ২০১৪ সালের মধ্যে প্রকাশিত হবে। এর আগে ২০০৭ সালে ৩৬০০ জন বিশেষজ্ঞ বৈজ্ঞানিক তিন বছরের সমবেত প্রচেষ্টায় আইপিসিসি-র চতুর্থ মূল্যায়ন প্রতিবেদনটি প্রকাশ করেন। এ থেকে জানা যায় যে, ১৭৫০ সাল থেকে মানুষের ক্রিয়াকর্মের ফলে বিশ্বের বায়ুমণ্ডলে কার্বন ডাইঅক্সাইড (CO_2), মিথেন ও নাইট্রাস অক্সাইডের পরিমাণ বিশেষভাবে বেড়ে যাচ্ছে। হাজার হাজার বছর ধরে জমে থাকা বরফ নিয়ে গবেষণা করে জানা যায় যে শিল্পোন্নয়নের পূর্বে যে অবস্থা ছিল তার থেকে বর্তমান অবস্থা অনেকটাই খারাপ। ২০৫০ সালের মধ্যে মধ্য এবং দক্ষিণ-পূর্ব এশিয়াতে বিশেষ করে বৃহৎ নদী অববাহিকায় বিশুদ্ধ পানির পরিমাণ কমে যাওয়ার আশঙ্কা রয়েছে।

বায়ুমণ্ডলের উষ্ণ হাওয়া এবং বৃষ্টির গতি প্রকৃতি ব্যাহত হওয়ার ফলে ভয়াবহ বন্যা অথবা দীর্ঘস্থায়ী খরা হয়। যখন উষ্ণতার ফলে সমুদ্রের জলস্তর বৃদ্ধি পায়, তখন উপকূলীয় এলাকায় ও দ্বীপে বসবাসকারীদের জীবনযাত্রা বিপন্ন হয়।

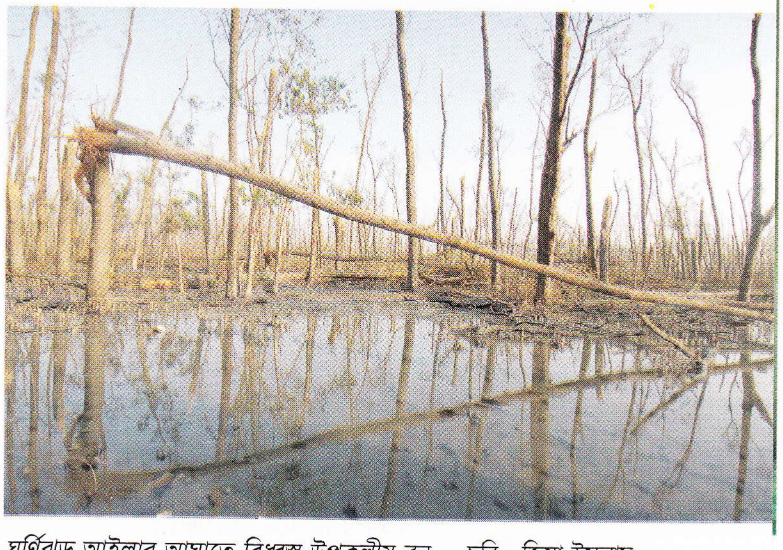

ঘূর্ণিঝড় আইলার আঘাতে বিধ্বস্ত উপকূলীয় বন ছবি : জিয়া ইসলাম

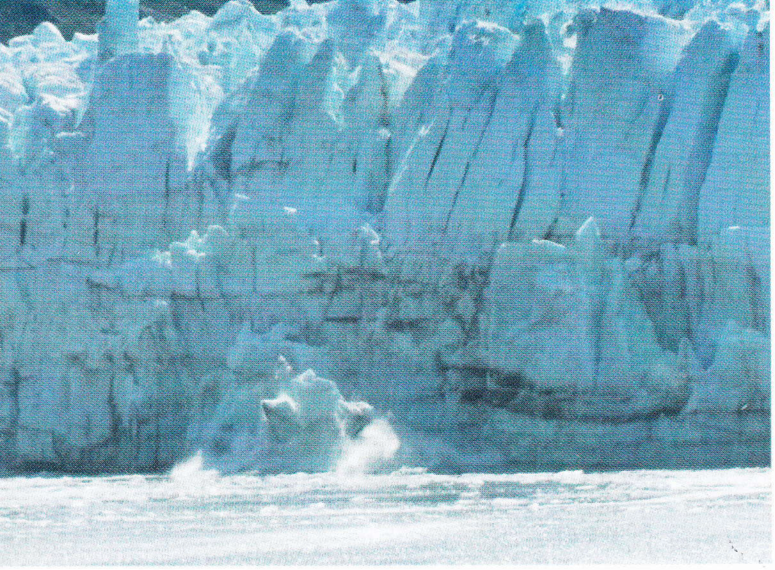

উভয় মেরুতে হাজার হাজার বছর ধরে জমে থাকা বরফ (গ্ল্যাসিয়ার) জলবায়ু পরিবর্তনের ফলে গলে যেতে শুরু করেছে

সমুদ্রের জলরাশি কতোগুলো বিশেষ কারণে বাড়তে পারে। তাপবৃদ্ধির ফলে উচ্চতা বাড়তে পারে, কারণ গরম পানি ঠাণ্ডা পানির থেকে বেশি জায়গা নেয়। হিমবাহ গলে যাবার ফলে সমুদ্রে পানির পরিমাণ বাড়তে পারে। লবণাক্ততার পরিবর্তনেও উচ্চতা বাড়তে পারে। বিশুদ্ধ পানি লবণাক্ত পানির চেয়ে কম ঘন ফলে একই পরিমাণ লবণাক্ত পানি বিশুদ্ধ পানির চেয়ে বেশি জায়গা নেয়।

উপগ্রহ এবং ফ্লোট (যান্ত্রিক বস্তু মহাসমুদ্রে ভাসমান)- থেকে পাওয়া তথ্যের উপর নির্ভর করে একদল সমুদ্র বিশেষজ্ঞ ২০০৬ সালের জুন মাসে ঘোষণা করেন যে, ১৯৯৩ থেকে ২০০৫ সালের মধ্যে সমুদ্রের পানির স্তর প্রতি বছরে গড়ে (প্রায়) ৩ মিলিমিটার হারে (০.১ ইঞ্চি) বৃদ্ধি পেয়েছে। ভবিষ্যতে উচ্চতর তাপমাত্রার জন্য হিমবাহ গলে যাবার ফলে প্রথমে অতর্কিত বন্যা এবং তার পরে পানির আকাল দেখা দেবে।

লক্ষ্য করো

- উপকূলীয় অঞ্চল বিশেষ করে ঘনবসতি অধ্যুষিত দক্ষিণ, পূর্ব এবং দক্ষিণ পূর্ব এশিয়ার বৃহৎ বদ্বীপগুলো সমুদ্রের বেড়ে যাওয়া পানি প্লাবনের ফলে বড় ধরনের সমস্যার সম্মুখীন হবে, বৃহৎ বদ্বীপে নদী থেকেও বন্যা হতে পারে।
- ধারণা করা হচ্ছে জলবায়ু পরিবর্তনের ফলে আমাদের প্রাকৃতিক সম্পদ এবং পরিবেশের উপর যৌগিক চাপের সৃষ্টি হবে, যা নগরায়ন, শিল্পায়ন ও অর্থনৈতিক উন্নয়নের উপর প্রভাব ফেলবে। বাড়বে স্বাস্থ্য ঝুঁকি। কারণ নগর সম্প্রসারণ ও শিল্পায়ন যেমন অর্থনৈতিক উন্নতির সাথে সম্পর্কযুক্ত, তেমনি মানুষের স্বাস্থ্যও এসবের সঙ্গে ওতোপ্রোতভাবে জড়িত ।
- উদরাময় রোগসহ অনান্য সংক্রামক রোগব্যাধি অঞ্চল বিশেষে প্রধানত বন্যা এবং খরার কারণে হয়। ধারণা করা হচ্ছে এই সকল রোগ পূর্ব, দক্ষিণ এবং দক্ষিণ-পূর্ব এশিয়ায় (বিশেষত বাংলাদেশে) পৃথিবীর জলীয়চক্রে পরিবর্তনের জন্য বেড়ে যাবে।

আইপিসিসি-র চতুর্থ মূল্যায়ন প্রতিবেদনে মানুষের স্বাস্থ্যে বড় রকমের প্রভাবের যে সব কথা বলা হয়েছে ঃ

- জলবায়ু পরিবর্তনের ফলে প্রাকৃতিক বিপর্যয় যেমন– উষ্ণপ্রবাহ, বন্যা এবং খরার মাধ্যমে প্রাণহানি এবং রোগব্যাধি হতে পারে।
- উপরন্তু অনেক রোগব্যাধি পরিবর্তনশীল তাপমাত্রা এবং বৃষ্টিপাতের পরিমাণের উপর ক্রিয়াশীল। এগুলোর মধ্যে আছে পতঙ্গবাহী রোগব্যাধি, যেমন– ম্যালেরিয়া, ডেঙ্গুজ্বর। এছাড়াও প্রধান সমস্যাগুলোর মধ্যে রয়েছে- পুষ্টির অভাব, শ্বাসকষ্ট, উদরাময়।
- জলবায়ু পরিবর্তনের ফলে এমনিতেই বিশ্বে রোগব্যাধির বোঝা বাড়ছে এবং এই বোঝা ভবিষ্যতে আরও বাড়বে বলে বিজ্ঞানীরা মনে করছেন।

অধ্যায় – ২

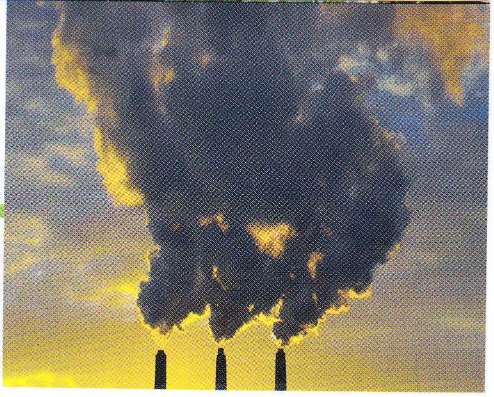

জলবায়ু পরিবর্তন ও স্বাস্থ্যসমস্যা

জলবায়ু পরিবর্তনের ফলে স্বাস্থ্য সম্পর্কে মূল্যায়ন

জলবায়ু পরিবর্তনের ফলে পৃথিবী আরও বেশি উষ্ণ ও অস্বাস্থ্যকর হয়ে উঠবে। উষ্ণতা বৃদ্ধিজনিত রোগবালাই বৃদ্ধি পাবে এবং মানুষের স্বাস্থ্যের উপর বিভিন্ন ক্ষতিকর প্রভাব ফেলবে।

হিট স্ট্রোক

উচ্চতর তাপমাত্রার ফলে উত্তাপজনিত অসুস্থতা ঘনঘন দেখা দেবে, যেমন–গরমের ফলে নিঃশ্বেষণ ও সর্দিগর্মি, হিট স্ট্রোক এবং বিভিন্ন সঞ্চালন প্রক্রিয়ায় সমস্যা, শ্বাসপ্রশ্বাস ও শিরা উপশিরা সংক্রান্ত বিরাজমান সমস্যাগুলো আরও বৃদ্ধি পাবে।

উষ্ণতা বৃদ্ধির ফলে বিশেষ করে শহর এলাকায় মৃত্যুর হার বেড়ে যাবে। উষ্ণপ্রবাহের সময় রাতের অধিক তাপমাত্রা মানুষের স্বাস্থ্যকে প্রভাবিত করবে, কারণ রাতের তুলনামূলক ঠাণ্ডা আবহাওয়া দিনের গরমকে প্রশমিত করে স্বস্তি আনে।

শ্বাসপ্রশ্বাস সংক্রান্ত রোগ

শ্বাসপ্রশ্বাস সংক্রান্ত রোগে নিঃশ্বাসের কষ্ট হয় এবং রক্তে অক্সিজেনের মাত্রা স্বাভাবিকের চেয়ে কমে যায়। উন্নয়নশীল দেশগুলোতে শ্বাসপ্রশ্বাসজনিত রোগে অনেক মানুষ মারা যায় এবং উন্নত দেশগুলোতে শিশুদের অসুস্থতার প্রধান কারণ এই রোগ। ১৯৯০ সালে বিশ্ব জুড়ে শ্বাসজনিত রোগে বহু লোক অসুস্থ এবং বিকলাঙ্গ হয়। ২০২০ সালের মধ্যে সারা পৃথিবীতে স্বাস্থ্য অবনতির যে কারণগুলো দেখা যাচ্ছে তার মধ্যে শ্বাসজনিত রোগ প্রথম দশটি কারণের মধ্যে একটি। প্রকৃত অর্থে ১৯৮০ সাল থেকে বহু দেশে হাঁপানির প্রকোপ চারগুণ বৃদ্ধি পেয়েছে। শ্বাসজনিত রোগ যেমন, হাঁপানি ও অ্যালার্জি বিভিন্ন কারণে হতে পারে। এই কারণগুলো মানুষের বংশানুগতিক ইতিহাস, জীবনধারা এবং যে পরিবেশে বাস করে তার সাথে সম্পর্কযুক্ত। উত্তাপ রাসায়নিক প্রক্রিয়াকে ত্বরান্বিত করে; ফলশ্রুতিতে ওজোন থেকে আসা দূষণকে বাড়িয়ে দিতে পারে।

ছ'টি প্রধান গ্রিনহাউস গ্যাস

নাম	বর্ণনা
জলীয় বাষ্প	বায়ুমণ্ডলে এই গ্যাসটি প্রচুর পরিমাণে পাওয়া যায় এবং পৃথিবীর সমস্ত জলাধার থেকে পানি বাষ্পীভূত হয়ে এই গ্যাস তৈরি হয়।
কার্বন ডাইঅক্সাইড (CO_2)	ভূগর্ভস্থ জ্বালানি এবং দাবানলের থেকে উৎপন্ন হয়।
মিথেন (CH_4)	পশুপালন, জলসিঞ্চন দ্বারা চাষবাস এবং তেল নিষ্পেষণ এই গ্রিনহাউস গ্যাসটি যথেষ্ট পরিমাণে নিঃসৃত করে।
নাইট্রাস অক্সাইড (N_2O)	ভূগর্ভস্থ বা প্রস্তরীভূত কয়লাকে জ্বালালে তার থেকে উদ্ভূত হয় এবং কৃষিভূমি কর্ষণের ফলেও নিঃসৃত হয়।
ওজোন (O_3)	উপরিস্থিত বায়ুমণ্ডলের যে রক্ষাত্মক স্তর আছে তার সর্ব প্রধান উপাদান যা পৃথিবীকে সূর্যের ক্ষতিকর অতিবেগুনী বা আল্ট্রাভায়োলেট রশ্মির বিকিরণ থেকে রক্ষা করে। 'ওজোন' একটি প্রাকৃতিক এবং মনুষ্যসৃষ্ট গ্যাস। ধোঁয়াশা এবং অতিরিক্ত বায়ুদূষণের ফলে তৈরি হওয়া এই গ্যাস মানুষের স্বাস্থ্যের জন্য ক্ষতিকারক।
ক্লোরোফ্লুরোকার্বনস (CFC_S)	ক্লোরিনযুক্ত গ্যাস যা রেফ্রিজারেটর, শীতাতপ নিয়ন্ত্রণ যন্ত্র, এ্যারোসল ছিটানোর যন্ত্র এবং পরিষ্কারক পদার্থের জন্য ব্যবহৃত হয়। ক্লোরোফ্লুরোকার্বন বায়ুমন্ডলে অবস্থিত ওজোন স্তরকে কমিয়ে দেয়।

উষ্ণতা বৃদ্ধি তথা জলবায়ু পরিবর্তনের কারণে কোনো কোনো গাছপালার রেণু উৎপাদন বেড়ে যাবে, সে জন্যে কিছু লোকের হাঁপানি এবং অ্যালার্জি বেড়ে যেতে পারে।

গৃহে এবং বাইরে বহুক্ষণ ধরে বায়ুদুষণের মধ্যে কাটানোর পর শিশুদের মধ্যে শ্বাসপ্রশ্বাসজনিত অসুস্থতা বেড়ে যেতে পারে।

মানুষের স্বাস্থ্যের ক্ষতি করতে পারে এমন কিছু মুখ্য বায়ু দূষণকারী গ্যাস হলো কার্বন ডাইঅক্সাইড, নাইট্রোজেন ডাইঅক্সাইড এবং সালফার ডাইঅক্সাইড। এই দূষণ রাস্তার যানবাহন ও শিল্পপ্রক্রিয়ার সাথে যুক্ত। জীবাশ্ম জ্বালানি থেকে যে বায়ু দূষকগুলোর উৎপত্তি হয় তার প্রতিক্রিয়া শুধুমাত্র সে অঞ্চলে আবদ্ধ থাকে না, হাজার হাজার মাইলব্যাপী ছড়িয়ে পড়ে। জিএইচজি-র ক্রমাগত নিঃসরণ বায়ুমণ্ডলের ওজোন স্তরকে আরও খারাপ অবস্থার দিকে নিয়ে যাবে।

জলাবদ্ধতা ও চর্মরোগ

বন্যা-জলোচ্ছ্বাস ইত্যাদি দুর্যোগের পর বহু স্থানে পানি আটকা পড়ে জলাবদ্ধতা সৃষ্টি করে এবং নিষ্কাশনের অভাবে দীর্ঘদিন পানি আবদ্ধ থাকে, কৃষিকাজকে ব্যাহত করে। আবদ্ধপানি নানা কারণে দূষিত হয়ে জনস্বাস্থ্যের প্রতি হুমকী হয়ে দেখা দেয়। অনেক সময় মানুষ ও জীবজন্তু আবদ্ধপানি ব্যবহার করতে বাধ্য হয়। ফলে পেটের পীড়া ও পানিবাহিত রোগ ছাড়াও বিভিন্ন চর্মরোগ দেখা দেয়।

আঘাত বা ক্ষত

আবহাওয়ার ঘন ঘন পরিবর্তনের ফলে উষ্ণপ্রবাহ, শৈত্যপ্রবাহ, সামুদ্রিকঝড়, ঘূর্ণিঝড়, তুফান এবং বন্যার ফলে মানুষের মৃত্যুর হার বেড়ে যাবে। নানারকম আঘাত যেমন– ক্ষয়ক্ষতি, শরীরের অঙ্গহানি, ছোটখাট আঘাত (যেমন হাড় ভাঙ্গা এবং কাটা-ছেড়া) এবং পানিতে ডুবে মানুষের মৃত্যু হতে পারে। বন্যা হচ্ছে সবচেয়ে সাধারণ আবহাওয়া বিপর্যয়। এর ফলে ১৯৯২ থেকে ২০০১ সালের মধ্যে সারা বিশ্বে ১,০০,০০০ লোকের মৃত্যু হয়েছে এবং একশ বিশ কোটি লোক উদ্বাস্তু হয়েছে।

২০০৭ সালে ভারত, বাংলাদেশ এবং নেপালে বিধ্বংসী বন্যা হয়। বাংলাদেশে সিডরের (প্রচণ্ড ঘূর্ণিঝড়) ফলে ৩৫০০ মানুষ মারা যায় এবং লক্ষ লক্ষ মানুষ গৃহহীন হয়। এসব দুর্যোগে সবচেয়ে বেশি বিপদের সম্মুখীন হয় শিশু, মহিলা (বিশেষ করে গর্ভবতী মহিলা) এবং বয়স্ক মানুষ। মহিলাদের উপর রোজগারের অতিরিক্ত বোঝা চাপে, কেননা প্রাকৃতিক বিপর্যয়ের পর তাদের স্বামীরা জীবিকা নির্বাহের জন্য শহরে চলে যায়। এ কারণে মহিলাদের পুরো পরিবারের দায়িত্বভার গ্রহণ করতে হয়।

পানিবাহিত রোগ

যে রোগ দূষিত পানির সাহায্যে বিস্তার লাভ করে তাকে পানিবাহিত রোগ বলা হয়। পানি যখন মানুষের ও জীবজন্তুর মলমূত্র দ্বারা দূষিত হয়, তখন সেখানে রোগ-জীবাণুবাহী অণুজীব থাকে। জমিতে ব্যবহৃত কীটনাশক, আবর্জনা, পয়ঃনিষ্কাশন ব্যবস্থা, বাসস্থান অথবা শিল্পকারখানায় ব্যবহৃত রাসায়নিক পদার্থ থেকে নিঃসৃত বর্জ্য সমতলের পানি দূষিত করে। উন্নয়নশীল দেশগুলোতে বেশির ভাগ রোগ পানি থেকে সংক্রমণ হয়– তার মধ্যে ডায়রিয়া ও আমাশয় অন্যতম এবং এতে শিশুদের মৃত্যু হার সব চেয়ে বেশি।

পানি এবং জনস্বাস্থ্য বিষয়ে বিশ্বব্যাপী একটি করুণ চিত্র রয়েছে। সারা পৃথিবীতে ১১০ কোটি লোকের এখনো বিশুদ্ধ পানীয় জলের সংস্থান নেই এবং প্রায় ২৪০ কোটি লোকের পর্যাপ্ত স্বাস্থ্যসম্মত পয়ঃনিষ্কাশন ব্যবস্থা নেই। বাংলাদেশের ক্ষেত্রেও সবার জন্য স্বাস্থ্যকর স্যানিটেশন ব্যবস্থা এখনো নিশ্চিত করা যায় নি।

উন্নয়নশীল দেশগুলোতে সাধারণত দূষিত পানি এবং অস্বাস্থ্যকর খাদ্যের কারণে রোগ বিস্তার লাভ করে। উষ্ণতর তাপমাত্রা বন্যার আশঙ্কা বাড়িয়ে দেবে ও অসুস্থতা বাড়াবে এবং পানি দূষণে কলেরা, উদরাময় এবং টাইফয়েড জাতীয় রোগ বেড়ে যাবে। পানি নিষ্কাশন ব্যবস্থার অভাবে এই সমস্যাটি আরও জটিল আকার ধারণ করবে। সামগ্রিকভাবে জলবায়ু পরিবর্তন উন্নয়নশীল দেশগুলোতে উদরাময়ের মতো অসুখ ২০২০ সালের মধ্যে প্রায় ২ থেকে ৫ শতাংশ বাড়িয়ে দেবে।

তাছাড়া তাপমাত্রা উষ্ণ থাকলে ঘনঘন সমুদ্রে শৈবাল জন্মাবে, বিশেষ করে যেখানে দূষিত পানি রয়েছে। সমুদ্রের শৈবালের সাথে কলেরা জীবাণুর সংশ্লিষ্টতা থাকার কারণে কলেরার প্রাদুর্ভাব বেড়ে যাবে।

কীটপতঙ্গবাহিত (ভেক্টর) রোগ

সংক্রামক রোগ পৃথিবীর অন্যতম প্রধান ঘাতক। জলবায়ু পরিবর্তনের কারণে কিছু সংক্রামক রোগের প্রকোপ বেড়ে যাবে– বিশেষ করে উষ্ণ অঞ্চলে মশা ও অন্যান্য কীটপতঙ্গের দ্বারা যে সব রোগগুলো ছড়ায়। জলবায়ু পরিবর্তনে 'ভেক্টর অণুজীব' যেমন, মশা এবং ইঁদুর অনুকূল পরিবেশ পেয়ে তাদের বংশ বৃদ্ধি করতে পারবে। এসব রোগের মধ্যে রয়েছে ম্যালেরিয়া, ডেঙ্গুজ্বর, পীতজ্বর ও এনকেফালাইটিস বা মস্তিষ্ক প্রদাহ ইত্যাদি। অন্যান্য ব্যাধি যেমন– চিকুনগুনিয়া এবং পীতজ্বর (দুটোই মশাবাহিত), সিস্টোসোমিয়াসিস (বাহক: স্থলশামুক), কালাজ্বর (বাহক: বেলে মাছি) এবং লাইম রোগ (বাহক: এঁটেল পোকা বা টিক) বাড়ারও শঙ্কা আছে। জলবায়ু পরিবর্তনের ফলে অনেক এলাকায় আঞ্চলিক রোগের (এনডেমিক) প্রাদুর্ভাব বেড়ে যাবে। উষ্ণতর তাপমাত্রা, তার সাথে বৃষ্টিপাতের ধারা এসব অঞ্চলে রোগ সংক্রমণের ঋতুকে কিছু কিছু অঞ্চলে বাড়িয়ে দিতে পারে, যেখানে আগে থেকেই সেই রোগটি বিদ্যমান ছিল। রোগমুক্ত অঞ্চলগুলোতে জলবায়ু পরিবর্তনের ফলে কতগুলো বিশেষ ভেক্টরবাহি রোগের উদ্ভব হতে পারে। যেমন পার্বত্য অঞ্চলে ম্যালেরিয়ার প্রকোপ এবং জলবায়ু পরিবর্তনের ফলে সমতল কিংবা উপকূলীয় অঞ্চলেও এর প্রকোপ বেড়ে যেতে পারে।

ম্যালেরিয়া

ম্যালেরিয়া একটি মশাবাহিত রোগ। ম্যালেরিয়া একটি পরজীবী সংক্রমণ যা আক্রান্ত স্ত্রী অ্যানোফিলিস মশার কামড় থেকে মানুষের মাঝে ছড়ায়। ম্যালেরিয়ায় আক্রান্ত মানুষের মধ্যে যে সমস্যা হয় তা মারাত্মক।

ম্যালেরিয়ার জীবাণু (প্লাজমোডিয়াম) দ্রুত রক্তের মধ্যে দিয়ে লিভারে ছড়িয়ে আবার রক্তকণিকায় ফিরে আসে এবং শেষ পর্যন্ত লোহিত রক্তকণিকায় এসে স্থায়ী হয় ও বংশবৃদ্ধি করে এবং নতুন পরজীবী হিসেবে প্রকাশ পায়। এই পরজীবীগুলো পরিমাণে অনেক বেশি থাকে এবং স্নায়ুতন্ত্র, লিভার ও কিডনির ক্ষতি করে।

বিশ্বে প্রতি বছর ম্যালেরিয়ায় প্রায় দশ লক্ষ লোকের প্রাণহানি ঘটে। এর এক বিরাট অংশের শিকার অনূর্ধ পাঁচ বছরের শিশুরা। ম্যালেরিয়ার জীবাণুবাহী মশা উপযুক্ত পানি নিষ্কাশন ব্যবস্থা দ্বারা আয়ত্ত্বে আনা যেতে পারে, কারণ মশা নিজের বংশ বিস্তারের জন্য পানির উপর নির্ভরশীল।

ডেঙ্গু জ্বর

ম্যালেরিয়ার মতো ডেঙ্গুও মশাবাহিত রোগ। ডেঙ্গুর জীবাণু আক্রান্ত স্ত্রী এডিস মশার কামড় থেকে মানুষের মধ্যে এটি সংক্রমিত হয়। মশাগুলো সাধারণত আক্রান্ত ব্যক্তির রক্ত শোষণ করার সময় জীবাণু গ্রহণ করে। আক্রান্ত মশা অন্য ব্যক্তিকে কামড়ানোর সময় রোগ সংক্রমিত হয়। ডেঙ্গু পৃথিবীর ট্রপিক্যাল অথবা উষ্ণ আবহাওয়া অঞ্চলে বিরাজমান, বিশেষ করে শহর এবং শহরতলি এলাকায়। ডেঙ্গু হেমোরেজিক ফিভার (ডিএইচএফ) একটি মারাত্মক ব্যাধি, প্রথম এটি সনাক্ত হয় ১৯৫০ সালে, সেই সময় ফিলিপাইনস এবং থাইল্যাণ্ডে ডেঙ্গু মহামারী হয়। ডিএইচএফ বেশিরভাগ এশিয়ার দেশগুলোতে দেখা যায় এবং বেশিরভাগ শিশুর মৃত্যুর কারণ। সাম্প্রতিক সময়ে বাংলাদেশেও এর প্রকোপ বেড়েছে। ধারণা করা হচ্ছে জলবায়ু পরিবর্তনের ফলে এর প্রকোপ আরও বেড়ে যাবে।

জাপানিজ এনকেফালাইটিস (জেই)

জাপানিজ এনকেফালাইটিস হচ্ছে ভাইরাস জনিত মস্তিষ্কের প্রদাহ। এই রোগ ফ্ল্যাভোভাইরাস থেকে হয় এবং কিউলেক্স মশার কামড় থেকে সংক্রমিত হয়। শুকর জাপানিজ এনকেফালাইটিস জীবাণু বহন করতে পারে। এই রোগটি এশিয়ার অনেক দেশে বিদ্যমান। বর্ষার সময় বড় আকারে এই রোগের খবর পাওয়া যায়। যে সমস্ত অঞ্চলে এই রোগ হয় সেখানে শিশুদের রোগ প্রতিরোধক টিকা দেওয়া উচিত।

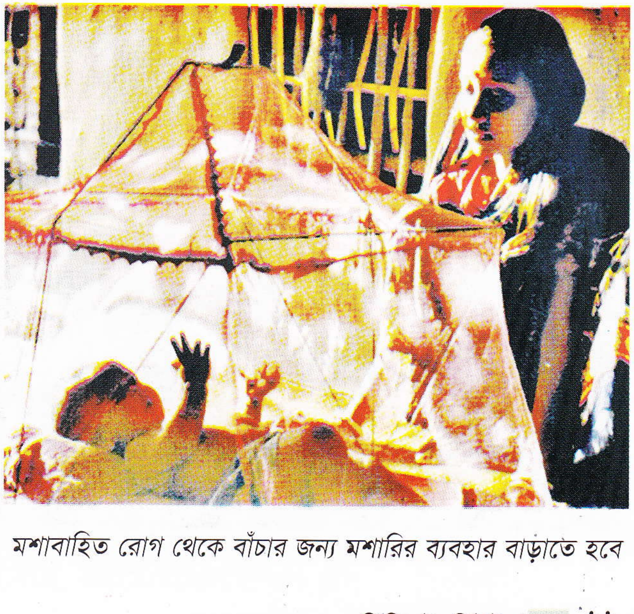

মশাবাহিত রোগ থেকে বাঁচার জন্য মশারির ব্যবহার বাড়াতে হবে

খাদ্য সমস্যা

খাদ্য উৎপাদন জলবায়ু পরিবর্তনের কারণে ভীষণভাবে ক্ষতিগ্রস্ত হবে। জলবায়ু পরিবর্তনের ফলে, তাপমাত্রা ও বৃষ্টিপাতের তারতম্যের কারণে, জমির আর্দ্রতা ও উর্বরতায় পরিবর্তন দেখা দেবে। শস্য ধ্বংসকারী কীটপতঙ্গগুলো অনুকূল অবস্থা পাওয়ার ফলে তাদের সংখ্যা বৃদ্ধি পাবে। খাদ্য সুরক্ষা ব্যবস্থাপনায় পুষ্টির অভাব দেখা দেবে। এ কারণে শিশুদের দৈহিক বৃদ্ধি ও বিকাশ কমে যাবে। পুষ্টির অভাব ও খাবারের অভাব প্রাপ্তবয়স্কদেরও স্বাস্থ্যের ক্ষতি করবে। সামগ্রিকভাবে খাদ্য নিরাপত্তা বিঘ্নিত এবং ফসলহানির কারণে অপুষ্টি বেড়ে যাবে।

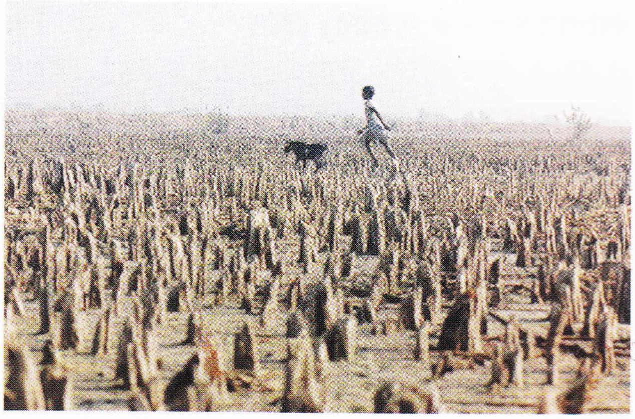

সামুদ্রিক ঝড়ের পর পশু খাদ্যের সন্ধানে ছুটে চলা   ছবি : জিয়া ইসলাম

পুষ্টির অভাব

চিকিৎসাশাস্ত্র অনুযায়ী পুষ্টি সমস্যা, অপর্যাপ্ত ও সুষম খাবারের অভাবে হয়ে থাকে। বেশিরভাগ ক্ষেত্রেই অপর্যাপ্ত খাবারে পুষ্টির পরিমাণ কমে যায়। পৃথিবীর দরিদ্রতম দেশগুলোতে রোগব্যাধি এবং পুষ্টির অভাবে মৃত্যুর হার নাটকীয়ভাবে বৃদ্ধি পাচ্ছে। কারণ শিল্পোন্নত দেশগুলো জলবায়ু পরিবর্তনের প্রধান হোতা। ব্রিটিশ এবং আমেরিকান বৈজ্ঞানিকদের প্রতিবেদন অনুযায়ী ১৯৯০ সালে পৃথিবী জুড়ে ৫২ কোটি লোক অভুক্ত ছিল (২০০৫ সালে প্রকাশিত)। বৈশ্বিক উষ্ণতা বৃদ্ধি না পেলে ২০৮০ সালে সেই সংখ্যা কমে ৩০ কোটিতে দাঁড়াবে বলে ধারণা করা হয়েছিল। কিন্তু বিশ্বব্যাপী উষ্ণতা বৃদ্ধির ফলে এর পরিমাণ ৩৮ কোটিতে বেড়ে যাবে বলে ধারণা করা হচ্ছে। প্রকৃতিতে বেশিমাত্রায় বৈরি আবহাওয়ার বিভিন্নতার প্রেক্ষিতে (উষ্ণপ্রবাহ, শৈত্যপ্রবাহ, ঘূর্ণিঝড়, জলোচ্ছ্বাস, বন্যা ইত্যাদি কারণে) দুর্ভোগ ও জীবনহানি হতে পারে। পৃথিবীতে ১৭০ কোটি লোক (পৃথিবীর জনসংখ্যার প্রায় এক তৃতীয়াংশ) এমন জায়গায় বাস করে যেখানে মাঝে মধ্যেই পানির অভাব হয়। ২০২৫ সাল নাগাদ পানির অভাবে ভুগবে প্রায় পাঁচশ কোটি লোক।

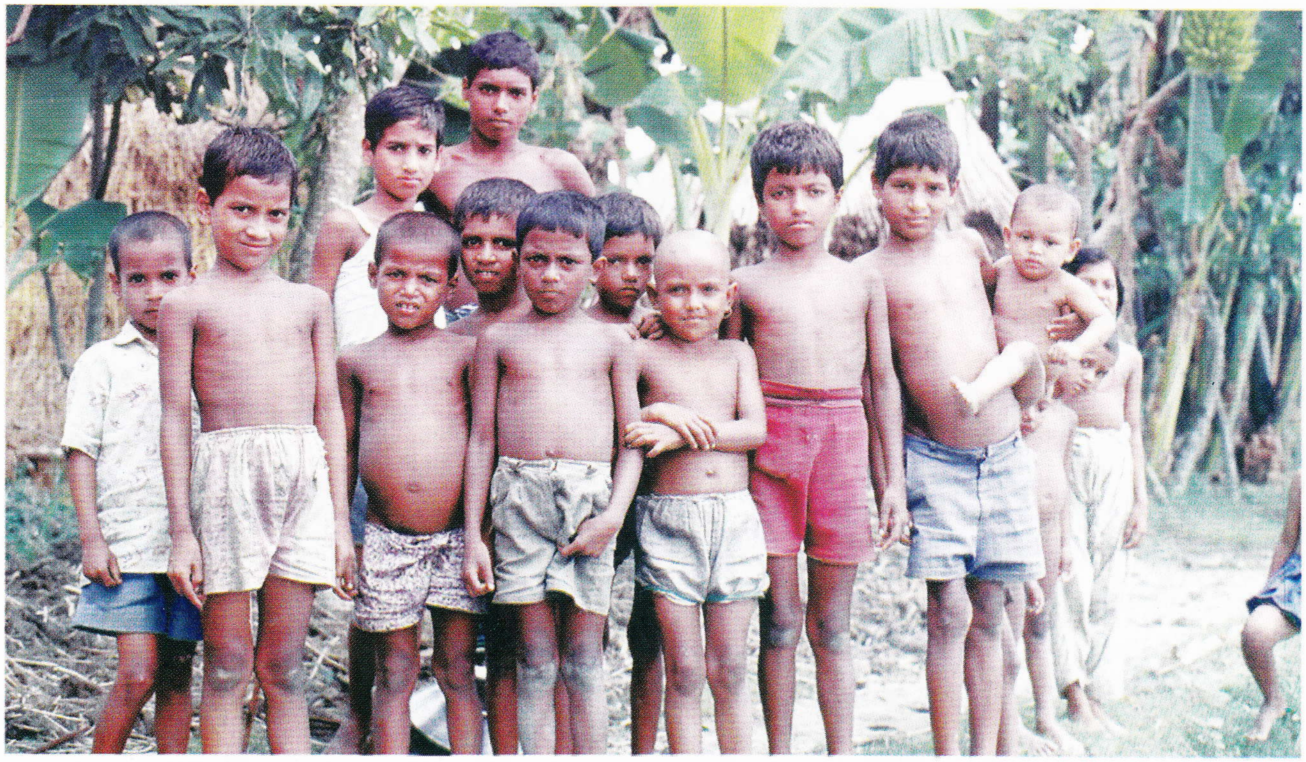

খাদ্য নিরাপত্তার অভাবে জলবায়ু পরিবর্তনে শিশুদের অপুষ্টি বেড়ে যেতে পারে।   ছবি : জাহাঙ্গীর সেলিম

বৃষ্টিপাতের ধারা পরিবর্তনের কারণে বিশুদ্ধ পানির যোগান কমে যাবে এবং মানুষ দুষিত পানির উপর নির্ভরশীল হবে, ফলে স্বাস্থ্য সংক্রান্ত সমস্যাগুলো যেমন– উদরাময়, শ্বাসকষ্ট ইত্যাদি অসুখ-বিসুখ ব্যাপকভাবে দেখা দেবে। পানি এবং খাবারের অভাবে কৃষকদের জীবন ধারণে বড় আঘাত আসবে এবং তারা দলে দলে জলবায়ু উদ্বাস্তু হিসাবে শহরে গিয়ে বসবাস শুরু করবে।

নদীর অববাহিকা কমে যাবে, উপকূলে লবণাক্ততা বেড়ে যাবে, মাছ ও জলীয় উদ্ভিদজগতের ক্ষতি হবে, উপকূল এবং এর আশেপাশে পলিমাটি কমে যাবার ফলে মাছ চাষের ক্ষতি হবে। কিন্তু উপকূল এবং নদীর ধারে বসবাসকারি মানুষের প্রোটিনের প্রধান উৎস হচ্ছে মাছ। এই সব পরিবর্তন ২০২০ সালের মধ্যেই শুরু হয়ে যেতে পারে।

মনোসামাজিক পরিচর্যা

প্রাকৃতিক বিপর্যয়, মানসিক চাপ সৃষ্টিকারী ঘটনা, অতিরিক্ত বিড়ম্বনা অথবা নিঃসঙ্গতা যে কোনো কারণেই পীড়াদায়ক অবস্থার সৃষ্টি হতে পারে। সাইকোসোশাল চাপের ফলে সাম্প্রতিক অথবা পুরানো ঘটনা ব্যক্তির দৈনন্দিন জীবনে নানাভাবে বিঘ্ন ঘটায় এবং মানসিক ও সামাজিক স্বাচ্ছন্দ্য বিনষ্ট করে। প্রাকৃতিক বিপর্যয়ের ফলে অনেক মানুষ গৃহহারা হচ্ছে এবং কর্মসংস্থানও হারাচ্ছে। দুর্যোগে বিপর্যস্ত পরিবারের লোকজনের বাসস্থান ও জীবিকা হারানোর মানসিক যন্ত্রণা সহ্য করতে হয়। কখনো কখনো এ ধরনের পীড়াদায়ক ঘটনার অভিজ্ঞতা মানসিক স্বাস্থ্যের উপর চিরদিনের জন্য ভয়ঙ্কর প্রভাব ফেলে।

যারা বিপর্যয়ের সম্মুখীন হয়েছেন তারা অভিঘাত পরবর্তী অসুস্থ্যতা (পোস্ট ট্রমাটিক স্ট্রেস ডিসর্ডার-পিটিএসডি) নামক রোগের শিকার হতে পারেন। এই চাপ বা পীড়াদায়ক পরিস্থিতি বিপর্যস্ত ব্যক্তির জীবন সংশয় অথবা গুরুতরভাবে আহত হবার সঙ্গে জড়িত। এই পীড়নে ক্ষতিগ্রস্তরা শুরুতে ক্ষিপ্ত অথবা বিচলিত ব্যবহার প্রদর্শন করে। তারা খুবই ভয়ার্ত, অসহায়, ক্রুদ্ধ, বিষাদগস্ত, আতঙ্কিত কিংবা নেতিবাচক ব্যবহার করতে পারেন। শিশুরা বারবার মানসিক পীড়নের সম্মুখীন হলে অনেক সময়েই ব্যথা ও দুঃখ ভোলার জন্য আবেগকে চাপা দিয়ে রাখে। একে বলা হয় দুঃখজনক ঘটনা থেকে নিজেকে আলাদা করে নেওয়া।

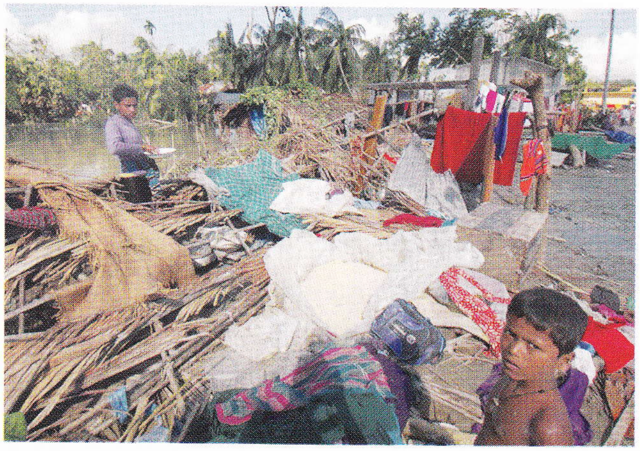

আইলায় বিধ্বস্ত ঘরে বিষাদগ্রস্ত শিশু *ছবি : জিয়া ইসলাম*

আক্রান্ত শিশুরা সেই সব পরিস্থিতি বা জায়গাকে এড়িয়ে চলে যেন তাদের ওই ঘটনা আবার মনে না পড়ে। এমনও হতে পারে এরা পরবর্তীকালে আবেগের আহ্বানে কম সাড়া দেবে, বিষাদগ্রস্ত হয়ে থাকবে, নিজেদের গুটিয়ে রাখবে এবং সকল প্রকার অনুভূতি থেকে নিজেকে সরিয়ে রাখবে, শিশুদের সৃষ্টিশীলতা কমে যাবে।

জলবায়ু পরিবর্তন সামাজিক ধারাকে ব্যাহত করতে পারে, অর্থনৈতিক অবনতি ঘটাতে পারে। কোনো কোনো অঞ্চলে কৃষি উৎপাদন ব্যাহত, অপ্রতুল পানি সম্পদ এবং বৈরি আবহাওয়ায় জলবায়ুর পরিবর্তনজনিত কারণে জনবসতির স্থানান্তরও ঘটতে পারে। এই অসুবিধাগুলো বাংলাদেশের মতো উন্নয়নশীল দেশগুলোতে অনেক বেশি ভয়ঙ্কর হয়ে উঠতে পারে, যার ফলে ক্ষতিগ্রস্ত অঞ্চলে জনস্বাস্থ্য ও জনকল্যাণের অবনতি ঘটতে পারে। জলবায়ু পরিবর্তন এবং জনস্বাস্থ্যের মধ্যে যে সম্পর্ক আছে তা সম্যকরূপে অনুধাবন করা প্রয়োজন। বাস্তবক্ষেত্রে বিশ্বব্যাপী উষ্ণতা বৃদ্ধি এবং জলবায়ু পরিবর্তনকে সহনীয় এবং গ্রহণযোগ্য করার ব্যাপারে সিদ্ধান্ত নিতে হবে। স্বাস্থ্য সংক্রান্ত ভাবনা চিন্তাগুলোকে অগ্রাধিকার দিতে হবে।

বাংলাদেশে ইতোমধ্যে জলবায়ুর প্রভাব প্রত্যক্ষভাবে পরিলক্ষিত হচ্ছে, যেমন– খরার প্রকোপ বৃদ্ধি, বৃষ্টিপাতের ধারায় পরিবর্তন (সময়ে সময়ে অনাবৃষ্টি, অতিবৃষ্টি, সামগ্রিক বৃষ্টিপাতের পরিমাণ হ্রাস), খাদ্য উৎপাদন ব্যাহত, ভূপৃষ্ঠের পানি অতিরিক্ত ব্যবহারের বিরুপ প্রভাব, ভূপৃষ্ঠে পানির স্তর নেমে যাওয়া, নদ-নদীতে স্বাভাবিক পানি প্রবাহ বাধাগ্রস্ত ও নাব্যতার অভাব, সামুদ্রিক জলোচ্ছ্বাস, ঘূর্ণিঝড়, বন্যা, প্লাবন ইত্যাদি ঘন ঘন আঘাত হানছে, বাড়ছে মনোসামাজিক চাপ।

জলবায়ু পরিবর্তন এবং মনুষ্য স্বাস্থ্যের মধ্যে যোগসূত্র

জলবায়ু পরিবর্তনের ফলে স্বাস্থ্য সম্বন্ধে শঙ্কা ও মূল্যায়ণ	
**জলবায়ু সংক্রান্ত পরিবর্তন**	**মানুষের স্বাস্থ্যে প্রভাব**
গরম হাওয়া, উষ্ণপ্রবাহ ও নিশ্চল বায়ুপুঞ্জ	• সর্দিগর্মি প্রধানত; বাচ্চা এবং বয়স্ককে আক্রান্ত করে।<br>• শ্বাস-প্রশ্বাস জনিত অসুখ বেড়ে যায়।<br>হৃদ এবং সংবহননালিকা সংক্রান্ত অসুখ (কার্ডিও-ভাসকুলার)।
উষ্ণতর তাপমাত্রা এবং বাধাপ্রাপ্ত বৃষ্টির প্রবাহ	• ম্যালেরিয়া, ডেঙ্গুজ্বর, জাপানীস এনকেফেলাইটিস এবং অন্যান্য অসুখ যা ভেক্টর বহন করে যেমন মশা, ইঁদুর এবং এঁটেল পোকা। এই সমস্ত অসুখগুলোর প্রকোপ বেড়ে যাবে।
অধিক বৃষ্টিপাতের ঘটনা, বন্যা	• দূষিত জল ও অস্বাস্থ্যকর খাবার খাওয়ার ফলে যে সমস্ত অসুখ হয়, তার প্রকোপ বেড়ে যাবে। বিশুদ্ধ জলের জোগান কমে যাবে এবং আবর্জনাময় ও অস্বাস্থ্যকর পরিবেশে বসবাসের ফলে কলেরা জাতীয় উদরসংক্রান্ত অসুখ বেড়ে যাবে।
খরা	• পুষ্টির অভাব এবং খাদ্যাভাব বিশেষভাবে শিশুদের বৃদ্ধি এবং বিকাশে ক্ষতি করবে।<br>• ফসল কমে যাওয়ায় কৃষক এবং তাদের পরিবার উদ্বিগ্ন হয়ে পড়বে (যাকে সাইকোসোশাল স্ট্রেস বলা হয়), বিশেষ করে যারা সম্ভবত ব্যাপক ও ক্রমাগত খরার ফলে ধার শোধ করতে অক্ষম।
তীব্র আবহাওয়ার ঘটনা (ঘূর্ণিঝড় এবং ঝড়)	• জীবনহানি, ক্ষত, জীবনব্যাপী প্রতিবন্ধী।<br>• ক্ষতিগ্রস্থ হবে জনস্বাস্থ্যের পরিকাঠামো, যেমন- স্বাস্থ্য কেন্দ্র, হাসপাতাল এবং চিকিৎসালয়। জীবনহানি, জমি ও সম্পদ ক্ষয়, প্রাকৃতিক বিপর্যয়ের ফলে বাস্তুহারা এবং বাসস্থান বদলাতে বাধ্য, এই সমস্ত মানসিক ও সামাজিক চাপের ফলে মানসিক স্বাস্থ্যের ক্ষতি।
সমুদ্রের জলস্তর বেড়ে যাওয়া এবং তটবর্তী ঘূর্ণিঝড়	• জীবিকাহানি এবং জমি নিশ্চিহ্ন হওয়ার ফলে দলে দলে লোকজনের স্থানান্তর থেকে সামাজিক ঘাত-প্রতিঘাত হতে পারে এবং মানসিক স্বাস্থ্যে ব্যাপক খারাপ প্রভাব পড়বে।

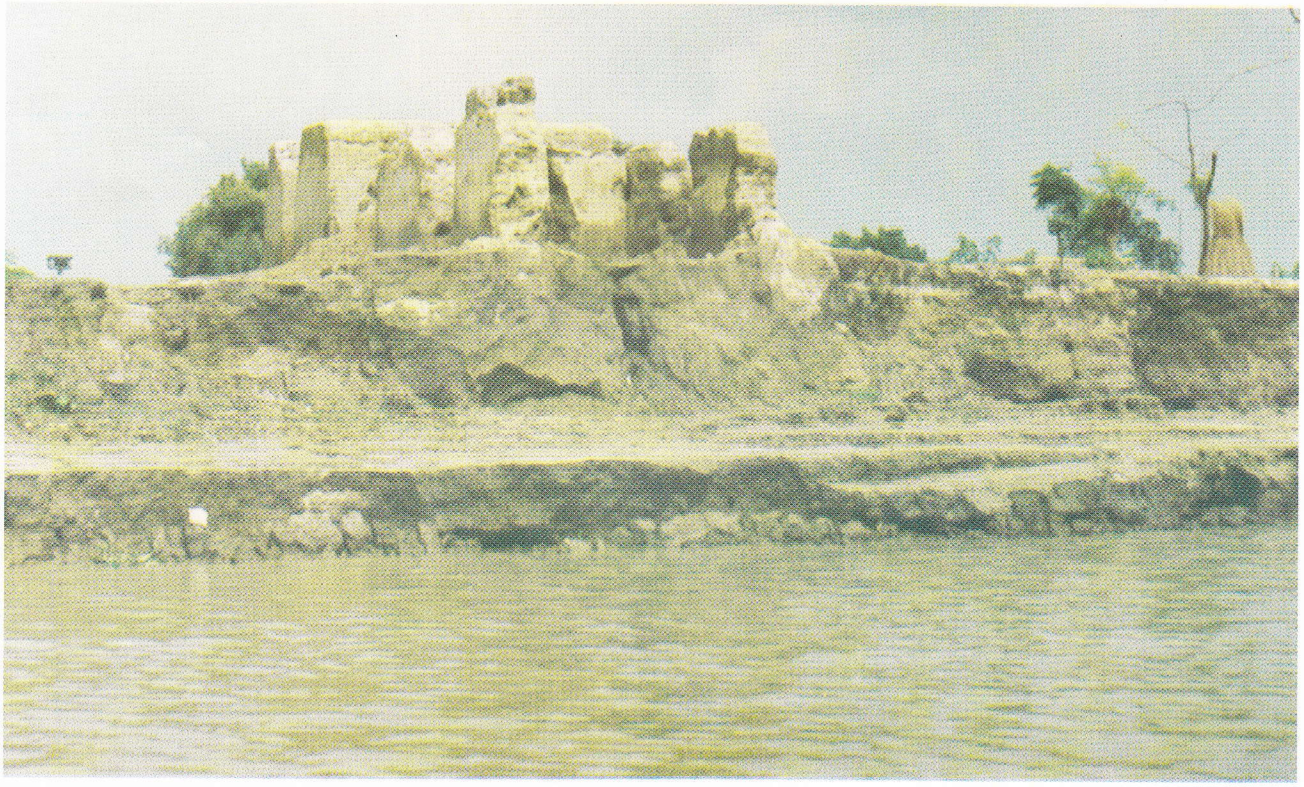

নদীভাঙ্গনে বিধ্বস্ত জনপদ- পরিত্যক্ত ঘরবাড়ি, দলে দলে লোকজনের স্থানান্তর *ছবি : জাহাঙ্গীর সেলিম*

অধ্যায় – ৩

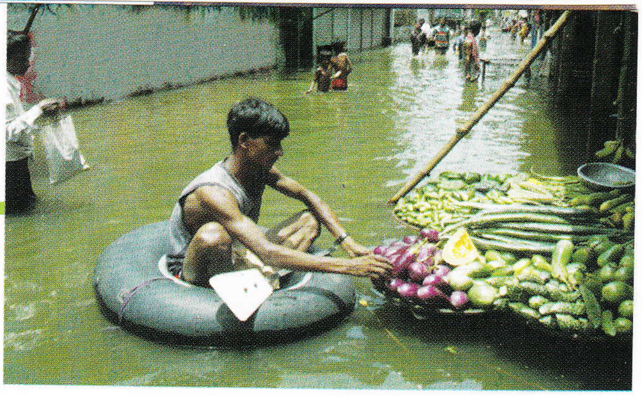

জলবায়ু পরিবর্তনজনিত স্বাস্থ্য ঝুঁকি মোকাবেলা

জলবায়ু পরিবর্তনের ফলে কীভাবে আমাদের কী কী ক্ষতির সম্মুখীন হতে হচ্ছে তা আমরা পূর্ববর্তী অধ্যায়গুলোতে জানতে পারলাম। অনেক পদক্ষেপ রয়েছে যার মাধ্যমে আমরা সহজেই উদ্ভূত পরিস্থিতি মোকাবেলায় সচেষ্ট ভূমিকা রাখতে পারি, যেমন- আমরা নিজেদের উদ্যোগ ও ব্যবস্থাপনার মাধ্যমে 'গ্রিনহাউস গ্যাস' কমাতে পারি এবং জলবায়ু পরিবর্তনের ঘড়িকে পিছিয়ে দিতে পারি।

জলবায়ু পরিবর্তনজনিত স্বাস্থ্য সমস্যা মূলত দুই উপায়ে প্রতিকার করা সম্ভব

- জলবায়ু পরিবর্তনের কারণগুলো এবং জনস্বাস্থ্যের উপর প্রতিক্রিয়া হ্রাস করা – যাকে **'মিটিগেশন'** বলা হয়।
- ভালোভাবে প্রস্তুত থেকে জলবায়ু পরিবর্তনজনিত স্বাস্থ্যহানি প্রতিহত করার ক্ষমতা বৃদ্ধি – যাকে **'অ্যাডাপটেশন'** বা অভিযোজন বলা হয়।

জলবায়ু পরিবর্তনজনিত ঝুঁকি হ্রাস বা মিটিগেশনের উপায়

জলবায়ু পরিবর্তনের ফলে জনস্বাস্থ্যের জন্য ক্ষতিকর দিক কমানোর জন্য ব্যক্তিগতভাবে আমরা এখনই কিছু করতে পারি। জলবায়ু পরিবর্তনের প্রভাব রোধ এবং হ্রাসের জন্য গুরুত্বপূর্ণ ভূমিকা পালন করে দৈনন্দিন জীবনে গ্রীণহাউস গ্যাস নিগর্মন কমিয়ে ফেলা সম্ভব। কার্বন নির্গমন ও প্রশমনের বিজ্ঞান ভিত্তিক হিসাব বিবেচনা করে ব্যক্তিগত গ্রিণহাউস গ্যাস নির্গমন সীমিত করতে পারি।

শক্তি অল্প খরচ করো এবং বেশিটাই বাঁচাও

পানির অপচয় করবে না। দাঁত মাজার সময়, কাপড় ধোঁয়া, গায়ে সাবান দেবার সময় পানির নল বন্ধ করে রাখো। প্রয়োজনীয় পরিমাণ পানি আলাদা পাত্রে নিয়ে ব্যবহার কর। পানির নলে ক্রুটি থাকলে সারিয়ে নিতে হবে। পানি পরিশোধণ এবং পাম্প করার কাজে যন্ত্রশক্তি ব্যবহার হয়। শক্তি বাঁচাও – পানি বাঁচাও।

দৈনন্দিন জীবনে যে কাজগুলো আমরা সহজেই করতে পারি ঃ

১. দাঁত মাজা ও মুখ ধোয়ার সময় পানি অপচয় না করা
২. গৃহস্থালি কাজে পরিশোধিত পানি ব্যবহার করে পানিবাহিত রোগ প্রতিরোধ করা
৩. কম শক্তির আলোর বাল্ব জ্বালানো এবং অপ্রয়োজনে নিভিয়ে রাখা
৪. কম্পিউটার, টেলিভিশন বা অন্যান্য বৈদ্যুতিক সরঞ্জাম-এর ন্যূনতম ব্যবহার করা
৫. সংক্রামক রোগ থেকে নিজেকে রক্ষা করা
৬. ব্যক্তিগত গাড়ি কম ব্যবহার করা, হাঁটা অথবা সাইকেলের ব্যবহার বাড়ানো
৭. গণপরিবহন আরও বেশি ব্যবহার করা
৮. বন্ধু বান্ধব প্রতিবেশীদের সঙ্গে একই বাহনে একসাথে স্কুল বা অফিসে যাওয়া, যা আনন্দদায়কও বটে
৯. শীতাতপনিয়ন্ত্রণ যন্ত্র বা পানি শীতল/গরম করার যন্ত্র কম ব্যবহার করা
১০. উন্নত নকশার মাধ্যমে বাড়িতে তাপরোধক ব্যবস্থাকে আরও বৃদ্ধি করা
১১. কাগজ ব্যবহারে সচেতন হওয়া
১২. মোবাইল ফোনে অপ্রয়োজনীয় কথা না বলা
১৩. সূর্যের শক্তিকে ব্যবহার করা, সৌরবিদ্যুৎ ব্যবহার
১৪. বেশি করে গাছ লাগানো
১৫. প্লাস্টিক ব্যাগের ব্যবহার বন্ধ করা
১৬. টক্সিক জাতীয় রাসায়নিক পদার্থের ব্যবহার কমিয়ে দেওয়া
১৭. কার্বন ব্যবহার কমিয়ে দেওয়া, বায়োগ্যাস ব্যবহার
১৮. তিনটি নীতিকে মেনে চলা–রিডিউস (ব্যবহার কমানো), রিসাইকেল (পুনরাবৃত্তি/তৈরি করা), রিইউজ (পুনরায় ব্যবহার করা)

পানিবাহিত রোগ প্রতিরোধ

বিশুদ্ধ খাবার পানির পাশাপাশি গৃহস্থালি যেমন রান্না, থালাবাসন ধোয়া, শাকসবজি ধোয়া ইত্যাদি কাজে পরিশোধিত পানি ব্যবহার করতে হবে। এর মাধ্যমে ডায়রিয়া, আমাশয়সহ বিভিন্ন পানিবাহিত রোগ থেকে আমরা সহজেই রক্ষা পেতে পারি।

বাল্ব

কম বিদ্যুৎ খরচ হয় এ রকম বাল্ব যেমন কম্প্যাক্ট ফ্লুরোসেন্ট ল্যাম্প (সিএফএল) বা লাইট এমিটিং ডায়োড (এলইডি) বাতি ব্যবহার কর।

কম বিদ্যুৎ ব্যবহৃত হয় এমন যন্ত্রপাতি কিনতে হবে

খোঁজ নিয়ে জিনিস কিনতে হবে, যেমন– কাপড় ধোয়ার মেশিন, রেফ্রিজারেটর, ডিশ ওয়াশার অথবা গ্যাসের চুলা ইত্যাদি কেনার সময় যে মডেলটিতে সবচেয়ে কম বিদ্যুৎ খরচ হবে এবং কেনার সাধ্যের মধ্যে আছে সেটি কিনতে হবে। এগুলোর দাম হয়তো একটু বেশি হতে পারে কিন্তু বিদ্যুতের খরচ কমাবে। ঠিক একই নিয়ম অফিসের জিনিস কেনার বেলায়ও প্রযোজ্য, যেমন– কম্পিউটার, ফটোকপিয়ার এবং প্রিন্টার ইত্যাদি। ব্যবহার শেষে টিভি, ভিডিও, স্টেরিও এবং কম্পিউটার বন্ধ রাখতে হবে, কারণ এগুলো 'স্ট্যাণ্ড বাই' মোডে থাকা অবস্থায় ১০ থেকে ৬০ শতাংশ শক্তি টানতে পারে। অপ্রয়োজনে আলো জ্বালাবে না, ইলেকট্রনিকস যন্ত্রপাতি ব্যবহার না করলে প্লাগ খুলে রাখো।

ফ্রিজ

প্রয়োজনের চেয়ে বেশি সময় ফ্রিজের দরজা খোলা রাখবে না, খাবার ঠাণ্ডা হলে ফ্রিজে ঢোকাবে, নিয়মিত বরফ ঝরিয়ে যন্ত্রটিকে সঠিক তাপমাত্রায় রাখবে। উনুন এবং ফ্রিজ পাশাপাশি না রাখাই উচিত।

কীটপতঙ্গ বাহিত রোগ হতে রক্ষা পাওয়ার উপায়

জানালায় জাল লাগিয়ে, মশা তাড়ানোর ক্রিম ব্যবহার করে এবং লম্বা হাতার জামা ও ফুলপ্যান্ট ব্যবহার করলে মশা থেকে রক্ষা পাওয়া যায়। কীটনাশক রাসায়নিক সম্বলিত বিশেষ মশারি ব্যবহার করো।

সবুজ থাকো

গাড়ি কিনতে হলে জ্বালানি ও পরিবেশ বান্ধব গাড়ি কিনবে। এতে তোমার পরিবারের অর্থের সাশ্রয় হবে এবং বায়ুমণ্ডলে কম পরিমাণে CO_2 ছড়াবে।

সব সময়ই লক্ষ্য রাখবে যে গাড়ির চাকায় ঠিকমতো হাওয়া আছে কিনা, এর ফলে ৫ শতাংশ জ্বালানি খরচ বাঁচবে। আত্মীয়-স্বজন বা বন্ধুদের সাথে ভাগ করে গাড়ি ব্যবহার করবে। গণপরিবহন ব্যবস্থার ব্যবহার বেশি করবে, যেমন লম্বা সফরের জন্য বাস বা ট্রেনে ভ্রমণ। বাজার হাটের জন্য হাঁটার চেষ্টা করবে অথবা সাইকেল ব্যবহার করবে। এতে স্বাস্থ্য ভালো থাকবে এবং আনন্দও পাবে।

এয়ারকণ্ডিশনারের ব্যবহার

এয়ারকণ্ডিশনারের থার্মোমিটারকে ৫ ডিগ্রি বাড়িয়ে বায়ুতে গ্যাস নিক্ষেপ কমিয়ে অর্ধেক করে দাও। বাড়ি ঘরে যে শক্তি ব্যবহার করা হয় তার প্রায় অর্ধেক খরচ হয় ঘর ঠাণ্ডা করতে। নিয়মিত এয়ারকণ্ডিশনারের ফিল্টার পরিষ্কার করবে। একটি পরিষ্কার হাওয়া পরিশোধক বছরে অনেক পরিমাণে কার্বন ডাইঅক্সাইড বাঁচাতে পারে। একই সাথে ডেঙ্গুর প্রকোপ থেকেও বাঁচাবে।

কাগজ বাঁচাও

কাগজের উভয় পৃষ্ঠা ব্যবহার করবে। প্রিন্টারে ছাপার আগে তথ্যগুলো স্ক্রিনে দেখে নাও। প্রত্যেক ব্যক্তির জন্য আলাদা করে ফটোকপি করার বদলে একটি কপি সকলকে দেখাও। এক দিকে ছাপযুক্ত কাগজগুলো ফেলে দেবে না। অন্য পিঠ খসড়ার কাজে ব্যবহার করবে।

নবায়নযোগ্য শক্তি ব্যবহার

নিজের বাড়ির ছাদে সূর্যশক্তিকে ব্যবহার করার জন্য সোলার প্যানেল স্থাপন কর।

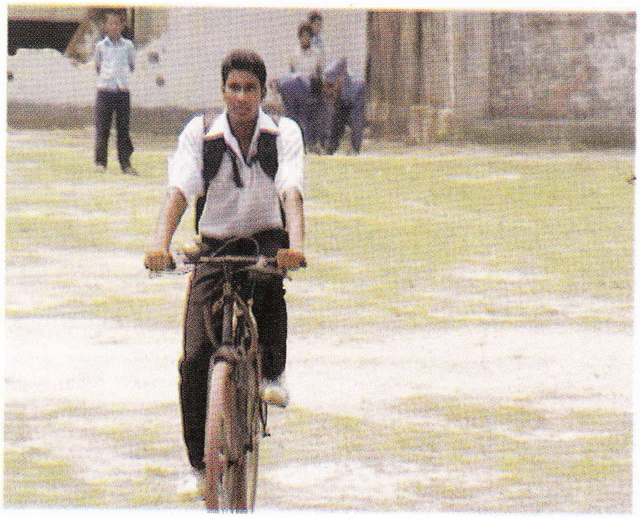

সাইকেল-এর ব্যবহার বাড়াতে হবে

নিজের বাড়ি অথবা অফিস একটি সূর্য শক্তির আড়ৎ-এ পরিণত কর। সূর্যের শক্তি প্রচুর এবং এ শক্তিকে অনেক ভাবে ব্যবহার করা সম্ভব।

গাছ লাগাও

আমাদের সরকার গাছ লাগানো এবং সবুজ বেষ্টনি গড়ে তোলার ব্যাপারে যথেষ্ট গুরুত্ব দিচ্ছে। প্রতি বছর বৃক্ষরোপণ সপ্তাহ পালন করা হয় এবং হাজার হাজার গাছ রোপণ করা হয়। সরকার ও অনেক বেসরকারি সংস্থা বিনা মূল্যে গাছের চারা বিতরন করে থাকে। এসবের সুযোগ তোমরাও গ্রহন করতে পার। স্কুল, কলেজ, রাস্তা ঘাট ও পতিত জায়গায় গাছ লাগাতে তোমাদের উদ্বুদ্ধ হতে হবে। প্রত্যেকে একটি করে ফলজ, বনজ ও ভেষজ গাছ লাগিয়ে দেশকে সবুজে শ্যামলে ভরে তুলতে হবে। গাছ লাগিয়ে এর যত্ন নিতে হবে যাতে গাছগুলো বেঁচে থাকে এবং বড় হয়। এতে পরিবেশের ভারসাম্য রক্ষা হবে। মানুষসহ সমস্ত প্রাণিকূল উপকৃত হবে।

প্লাস্টিকের ব্যাগ পরিহার

বাজারে যাবার সময় পরিবেশ বান্ধব পাট, কাপড় অথবা কাগজের তৈরি ব্যাগ ব্যবহার করবে।

রাসায়নিক জাতীয় পদার্থের ব্যবহার কমাও

ক্ষতিকারক রাসায়নিক পদার্থ, টক্সিক বা বিষাক্ত পদার্থ জমিতে না মেশানোই ভাল। প্রাকৃতিক উপাদান থেকে রং তৈরি কর এবং পোকামাকড় তাড়ানোর জন্য সমন্বিত বালাই দমন পদ্ধতি কাজে লাগাতে হবে।

কার্বন ব্যবহার কমাও, বায়োগ্যাস ব্যবহার করো

অনেক কম খরচে শক্তি বাঁচানো এবং কার্বন ব্যবহার কম করার ব্যবস্থা যে কেউই নিতে পারে। গৃহস্থালির কাজে বায়োগ্যাস ব্যবহার করো।

রিসাইকেল (পুনরোৎপাদন)

সমস্ত জিনিস রিসাইকেল করার চেষ্টা কর, বিকল হলে সারিয়ে নাও এবং পুনরায় ব্যবহার কর।

জঞ্জালের মূল্য আছে

বাড়ির আবর্জনা যেখানে সেখানে ফেলবে না। খোলা জায়গায় আবর্জনা পড়ে থাকলে সেখান থেকে মিথেন গ্যাস নির্গত হয় এবং বিশ্বব্যাপী উষ্ণতা বৃদ্ধিতে সাহায্য করে। আবর্জনা বেছে তা জৈবসারে রূপান্তর কর এবং রিসাইকেল করে পুনরায় ব্যবহার করা যায়। যেখানে সম্ভব জৈবজঞ্জালকে সার হিসাবে ব্যবহার কর। অপচয় কমাতে কম জঞ্জাল তৈরি কর। মোটরগাড়ি, রেডিয়েটর ইত্যাদিতে ব্যবহৃত রাসায়নিক তরল, গাড়ির পুরানো চাকা জঞ্জালে পরিণত না করে স্থানীয় গ্যাস বা পেট্রল পাম্পে অথবা গাড়ি মেরামতকারীর কাছে নিয়ে যাও।

রিডিউস বা ব্যবহার কমানো

দ্রব্যের সচেতন ব্যবহার সহজেই জঞ্জালের পরিমাণ কমিয়ে আনতে পারে। পানি, বিদ্যুৎ, জ্বালানিসহ শক্তির বিভিন্ন উৎসগুলোকে আমাদের সংরক্ষণ করতে হবে। যেকোনো প্রকারের অপচয় পরিহার করতে হবে। বাজার থেকে কোন পণ্য কিনতে হলে প্রত্যেকটির জন্য আলাদা প্যাকেট না কিনে একটি বড় প্যাকেট কিনলে জঞ্জাল কমে যাবে। পণ্যটি বাজারজাত করতে ব্যবহৃত শক্তির পরিমাণ কমে যাবে এবং মোড়কজাত করতেও বিভিন্ন দ্রব্যের ব্যবহার কমে যাবে।

রিইউজ (পুনরায় ব্যবহার করা)

পুনরায় ব্যবহার করা মানে শক্তি সঞ্চয়। কারণ এর ফলে কম পরিমাণ গাছ কাটতে হয়, নতুন করে কাঁচামাল কম ব্যবহার করতে হয়। কাগজ, প্লাস্টিক, ধাতব পদার্থ, ইলেকট্রনিক বর্জ্যসহ আরও অনেক কিছুই পুনরায় ব্যবহার করার জন্য আমাদের নতুন নতুন পদ্ধতি উদ্ভাবন করতে হবে।

> রিইউজ, রিসাইকেল ও রিডিউস-এর মাধ্যমে ২০০৮ সালে কার্বন ডাইঅক্সাইড নির্গমনের পরিমাণ ১৮২০ লক্ষ মেট্রিক টন কমানো সম্ভব হয়েছে। যা পৃথিবী থেকে প্রায় ৩৩০ লক্ষ গাড়ি সরিয়ে ফেলার সমান প্রভাব ফেলবে।

গণসচেতনতা গড়ে তোলা

জলবায়ু পরিবর্তনের ফলে স্বাস্থ্যে যে প্রতিক্রিয়া হচ্ছে সে ব্যাপারে সংবাদপত্রে লেখালেখি জনগণের গণসচেতনতা বাড়িয়ে তোলার একটি সহজ মাধ্যম এবং এটিই জনগণকে সচেতন করার উপযুক্ত কৌশল। এটি সকলকে আসল ঘটনা বুঝতে সাহায্য করে। জলবায়ু পরিবর্তন এবং পরিবেশিক স্বাস্থ্য সম্বন্ধে বিতর্ক, আলোচনা, পত্রিকা বিতরণ, ক্ষুদ্র পুস্তিকা এবং দেয়াল পত্রিকার মাধ্যমে প্রচার চালিয়ে যেতে হবে। নিজের পরিবার, বন্ধু, শিশু ও প্রতিবেশীদের এই প্রচেষ্টায় যুক্ত কর।

একটি পরিবেশ ও জনস্বাস্থ্য সচেতন সংগঠনে যোগ দাও। খোঁজ কর তোমার চারপাশে সংগঠনগুলো কী কাজ করছে, যদি না থাকে তাহলে জনস্বাস্থ্য সুরক্ষায় নতুন সংগঠন গড়ে তোলো। তোমার এলাকায় পরিবেশ সচেতনতা বিষয়ক অভিযান চালাও। কমিনিউটি ক্লিনিকে যোগাযোগ করো।

পরিবেশগত স্বাস্থ্য এবং সমাধান সম্পর্কে আশঙ্কা প্রকাশ ও সচেতন থাকো

পরিবেশগত ও স্বাস্থ্য বিষয়ক প্রচুর পড়াশুনা কর, এই বিষয়ে অবগত থাকো এবং অন্যের সঙ্গে তথ্য আদান প্রদান করো।

গ্রিনহাউস গ্যাস নির্গমন হ্রাস করাই তোমাদের উদ্দেশ্য হোক

এটি লক্ষ্যে পৌঁছানোর জন্য সবচেয়ে ভালো পথ। আমাদের দেশে নতুন জাতীয় আইন ও বিধান করা উচিত যার দ্বারা আমরা দূষণমুক্ত গাড়ি এবং দূষণমুক্ত বিদ্যুৎ উৎপাদন কেন্দ্র বানাতে পারি। গৃহে সূর্য শক্তি বা বায়ু শক্তি কাজে লাগানোর জন্য সরকার অনুদান দিয়ে পৃষ্ঠপোষকতা করার উদ্যোগ নিয়েছে। তোমাদের দৈনন্দিন ও সামাজিক জীবনে আচরণিক পরিবর্তনের মাধ্যমে জলবায়ু পরিবর্তনের স্বাস্থ্য ঝুঁকিগুলো কমাতে হবে। সুযোগের সদ্ব্যবহার করো।

এই সমস্ত কাজগুলো আমরা নিজেরা করতে পারি। আমাদের সমাজে জলবায়ু পরিবর্তনের জন্য বড় ধরনের পরিবর্তন আনতে হলে গণমানুষের সম্পৃক্ততা খুবই জরুরি। যেমন, পাড়া-পড়শির সঙ্গে যৌথভাবে জলাবদ্ধতা বা ডোবা-নালা বালি দিয়ে ভর্তি করে মশা প্রজননের উৎসগুলো নষ্ট করতে পারি। একটি উপযুক্ত নিষ্কাশন ব্যবস্থা তৈরি করে স্থানীয় সরকারের মাধ্যমে স্থায়ী সমাধানের জন্য আবেদন করতে পারি।

পৃথিবীর সকল দেশ কিয়োটা চুক্তির দ্বারা গ্রিনহাউস গ্যাস নির্ধারিত নিক্ষেপের সীমাতে পৌঁছাতে দায়বদ্ধ। যে সব দেশ এই আন্তর্জাতিক চুক্তিতে স্বাক্ষর করেছে তারা শর্তাবলি বাস্তবায়নে অঙ্গীকারবদ্ধ। গ্রিনহাউস গ্যাস নির্গমন কমানোর ব্যাপারে বেসরকারি সংস্থাগুলোর একটি বিশেষ ভূমিকা আছে।

আরও উন্নত প্রযুক্তি ব্যবহার করে জৈব জ্বালানির উপর নির্ভরতা কমিয়ে আনতে হবে। গাছপালার উপর নির্ভরশীল 'বায়ো ফুয়েল' একটি বিকল্প জ্বালানি হতে পারে যতক্ষণ পর্যন্ত তার জন্য বন বিনাশ, খাদ্যের উৎসে আঘাত অথবা আরও বর্ধিতভাবে জলবায়ুর পরিবর্তন না হচ্ছে।

স্বাস্থ্য বিভাগ গ্রিণহাউস গ্যাস হ্রাসের সহযোগিতা ও স্বাস্থ্যের উপকারিতার উপর জোর দেবে এবং জনস্বার্থে গণপরিবহন ব্যবহার করতে উদ্বুদ্ধ করবে। এটি বায়ু দূষণ হ্রাস করবে এবং দুর্ঘটনার মাত্রা কমাবে। নির্মল বায়ু সেবন এবং গাড়ি কম ব্যবহার করার ফলে অনেক বেশি শারীরিক প্রক্রিয়া সচল হবে, ফলে মানুষের স্থূলতা কমে যাবে, অসংক্রামক ব্যাধি কমে যাবে।

অ্যাডাপটেশন বা অভিযোজনের উপায়

অভিযোজন হচ্ছে সম্ভাব্য জলবায়ু পরিবর্তনের ঘনঘটা বা প্রতিক্রিয়ার মুখে পূর্বের অবস্থার সঙ্গে বর্তমান বাস্তবতার সমন্বয় সাধন। অর্থাৎ জলবায়ু পরিবর্তনের ফলে নতুন প্রতিবেশিক, সামাজিক অথবা অর্থনৈতিক অবস্থার সাথে খাপ খাইয়ে নেওয়াকে অভিযোজন বলা হয়। এই সমন্বয় প্রাকৃতিক নিয়মাবলি ও মানুষের কর্ম পদ্ধতির মধ্যে হতে পারে। এই প্রক্রিয়ায় অনুশীলন এবং কাঠামোগত পরিবর্তনের মাধ্যমে সম্ভাব্য দুর্যোগজনিত ক্ষয়ক্ষতি কমানো সম্ভব হতে পারে বা নতুন তৈরি হওয়া সুযোগের সদ্বব্যবহার করে সুফল পাওয়া যেতে পারে। অভিযোজন বলতে খাপ খাইয়ে নেওয়ার এই ধারাবাহিক প্রক্রিয়া এবং খাপ খাইয়ে নেওয়ার উপযুক্ত পরিবেশকে বোঝায়। প্রতিবেশ ব্যবস্থার ক্ষেত্রে অভিযোজন বলতে এমন পরিবর্তনকে বোঝায় যাতে করে প্রাণিকূল টিকে থাকতে এবং আরও বেশি সময় বা পুনঃউৎপাদনময় হতে পারে। সমাজবিজ্ঞানে অভিযোজন বলতে ব্যক্তিবর্গের উপযোগিতা এবং আর্থসামাজিক ব্যবস্থার সামগ্রিক আচার আচরণকে বোঝায়। জলবায়ু পরিবর্তনের ক্ষেত্রে অভিযোজন বলতে সংঘটিত বা আসন্ন জলবায়ু পরিবর্তনের ঘনঘটা এবং এর প্রতিক্রিয়া বা প্রভাবের সঙ্গে বিদ্যমান প্রতিবেশিক, সামাজিক ও অর্থনৈতিক ব্যবস্থার সমন্বয় সাধনকে বোঝায়। এই অভিযোজন প্রক্রিয়া জলবায়ু পরিবর্তনের সঙ্গে সম্পর্কিত সমস্যাগুলোর সমাধানের পথ খোঁজার সঙ্গে সঙ্গে নতুন প্রাপ্ত সুযোগ থেকে সুফল বয়ে আনার লক্ষ্যে পরিচালিত হয়। পরিবর্তিত জলবায়ুর সঙ্গে খাপ খাইয়ে নেওয়া একটি ধারাবাহিক প্রক্রিয়া ও প্রতিদিনের কাজ।

অভিযোজনের উদাহরণ

জনস্বাস্থ্যের নিয়মনীতিগুলো শুধু সাম্প্রতিক রোগের ক্ষেত্রকে বোঝায় না, বরং ভবিষ্যতের রোগগুলোকে কমানো বা মোকাবেলার জন্যও প্রযোজ্য হবে। সেজন্য রোগ প্রতিরোধ, নিয়ন্ত্রণ ও প্রশমনে সচেতনতা বৃদ্ধি ও রোগ মোকাবেলা করা ও কার্যকর ব্যবস্থা গ্রহণ করা খুবই জরুরি। এ ক্ষেত্রে একটি ইতিবাচক দিক হচ্ছে প্রচলিত স্বাস্থ্য ব্যবস্থাপনা ও কার্যক্রম বড় ধরনের বিপর্যয় রোধ করতে সচেষ্ট। তবে আরও সুসংহত উদ্যোগ গ্রহণ ও জনস্বাস্থ্যের উপর গুরুত্ব দেওয়া প্রয়োজন। সমন্বিত উদ্যোগ ভবিষ্যতে জলবায়ু পরিবর্তন জনিত স্বাস্থ্য সমস্যাদি প্রশমন করতে পারে।

সরকারের পাশাপাশি স্থানীয় সংগঠন ও বেসরকারি সংস্থাগুলোর একযোগে কাজ করা উচিত। বিশেষ করে উপকূল ও দূর্গম এলাকাগুলোতে আচরণিক পরিবর্তনের জন্য কার্যক্রম শক্তিশালী করা ও গণসচেতনতা বৃদ্ধি করা যার মাধ্যমে রোগব্যাধির হাত থেকে পরিত্রাণ পাওয়া সম্ভব। যেমন– বিশুদ্ধ পানীয়জলের সংস্থান, স্যানিটেশন এবং রক্ষণাবেক্ষণ ইত্যাদি। বিদ্যালয় স্বাস্থ্য কার্যক্রম ও কমিউনিটি ক্লিনিক এক্ষেত্রে গুরুত্বপূর্ণ ভূমিকা পালন করতে পারে। স্থানীয়ভাবে সরকারি স্বাস্থ্যকর্মীদের সাথে বিদ্যালয়ের ছাত্রছাত্রী, শিক্ষক ও অভিভাবকগণের একত্রে বিদ্যালয় স্বাস্থ্য কার্যক্রম পরিচালনায় কার্যকর পদক্ষেপ গ্রহণ প্রশংসনীয় উদ্যোগ হতে পারে।

অন্যান্য উপায় যা জলবায়ু পরিবর্তনের নেতিবাচক প্রভাব কমাবে তা হলো এমন ধরনের শস্য উৎপাদন এবং খরা সহনশীল, অল্প পানি ও লোনামাটিতে ফসল উৎপাদনের প্রক্রিয়া উদ্ভাবন করা। এ সবের পাশাপাশি উপকূলে কার্যকরি বন্যা নিয়ন্ত্রণের ব্যবস্থা করা, বিপজ্জনক এলাকাগুলোতে বসতি গড়ে তুলতে না দেওয়া ইত্যাদি বিষয়ে সরকারকে পদক্ষেপ নিতে হবে।

মিটিগেশন : জলবায়ু পরিবর্তনের প্রভাব রোধ এবং হ্রাসের জন্য কার্বন নির্গমন ও প্রশমনের বিজ্ঞান ভিত্তিক হিসাব বিবেচনা করে গ্রিনহাউস গ্যাস নির্গমন সীমিত করার উপায়কে মিটিগেশন বলে।

অ্যাডাপটেশন : জলবায়ু পরিবর্তনের ক্ষেত্রে অভিযোজন বলতে সংঘটিত বা আসন্ন জলবায়ু পরিবর্তনের ঘনঘটা এবং এর প্রতিক্রিয়া বা প্রভাবের সঙ্গে বিদ্যমান প্রতিবেশিক, সামাজিক ও অর্থনৈতিক ব্যবস্থার সমন্বয় সাধন বা খাপ খাওয়ানোকে বোঝায়।

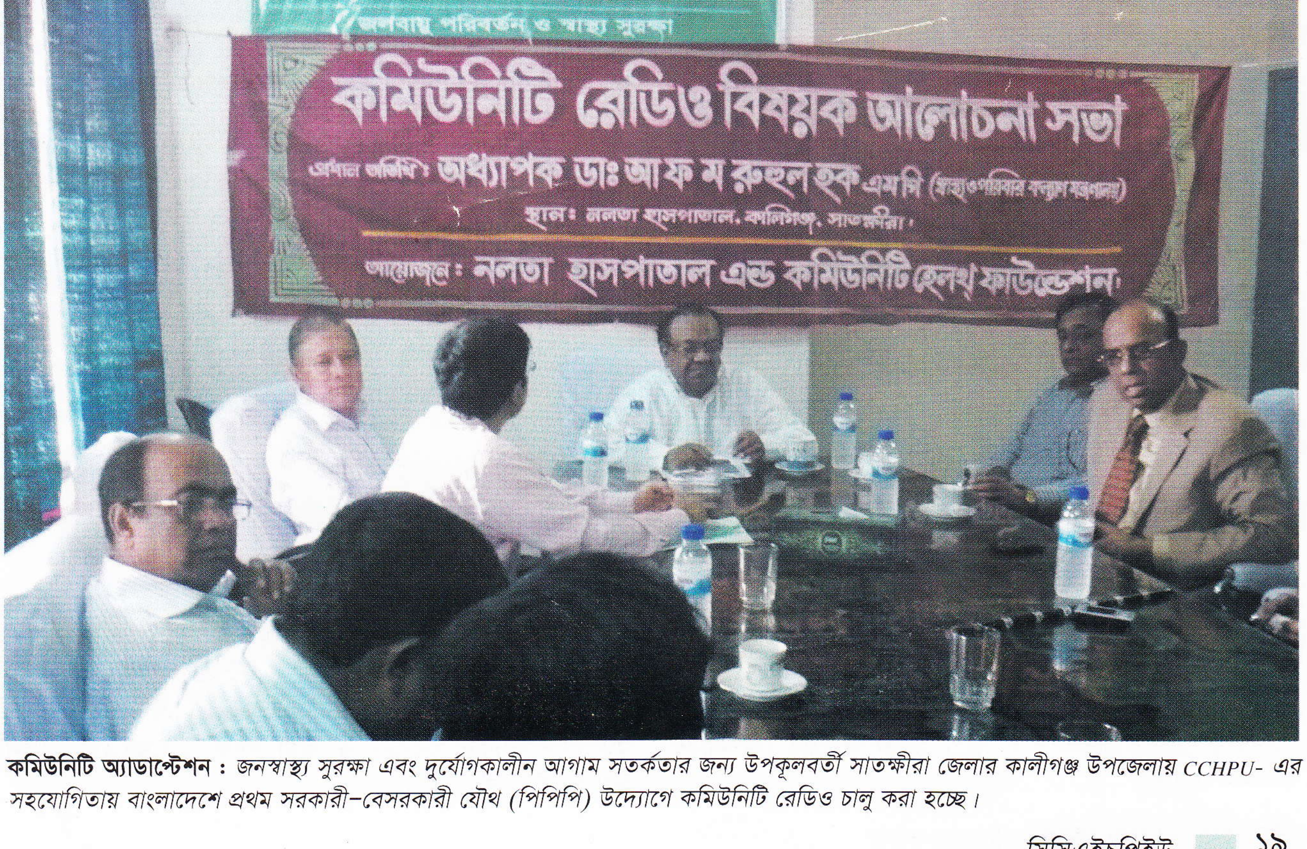

কমিউনিটি অ্যাডাপ্টেশন : *জনস্বাস্থ্য সুরক্ষা এবং দুর্যোগকালীন আগাম সতর্কতার জন্য উপকূলবর্তী সাতক্ষীরা জেলার কালীগঞ্জ উপজেলায় CCHPU- এর সহযোগিতায় বাংলাদেশে প্রথম সরকারী–বেসরকারী যৌথ (পিপিপি) উদ্যোগে কমিউনিটি রেডিও চালু করা হচ্ছে।*

সিসিএইচপিইউ-এর কিছু কর্মকান্ডের চিত্র

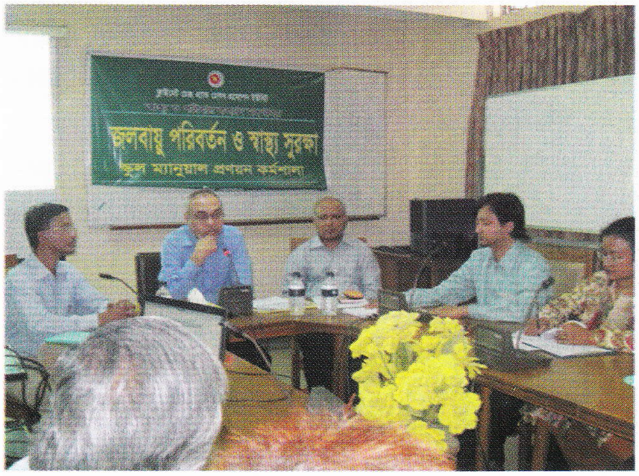

স্কুল ম্যানুয়াল তৈরির প্রথম কর্মশালায় তৎকালীন স্বাস্থ্য ও পরিবার কল্যাণ সচিব শেখ আলতাফ আলী

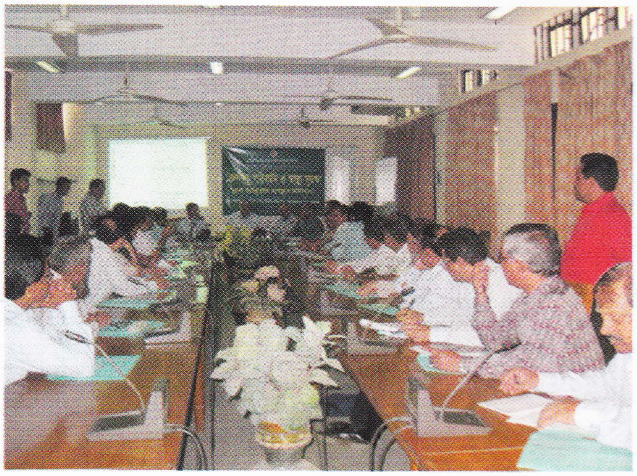

স্কুল ম্যানুয়াল তৈরির কর্মশালায় স্বাস্থ্য অধিদপ্তরের তৎকালীন মহাপরিচালক অধ্যাপক শাহ্ মনির হোসেনসহ বিশেষজ্ঞগণ

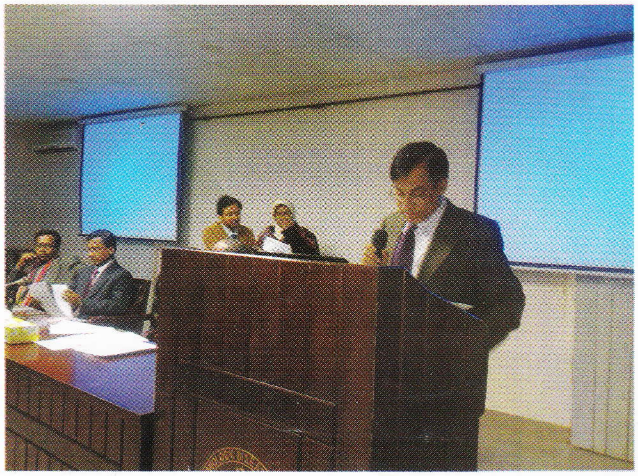

জলবায়ু পরিবর্তন ও স্বাস্থ্য সুরক্ষা প্রকল্পের বেইজলাইন সার্ভের তথ্য সংগ্রহকারীদের প্রশিক্ষণ কর্মশালায় প্রধান অতিথির বক্তব্য দিচ্ছেন স্বাস্থ্য ও পরিবার মন্ত্রণালয়ের সচিব মুহম্মদ হুমায়ুন কবির। অন্যান্যের মধ্যে উপস্থিত আছেন স্বাস্থ্য ও পরিবারকল্যাণ মন্ত্রণালয়ের প্রকল্প পরিচালক ও উপসচিব (জনস্বাস্থ্য) বেগম রাশেদা আকতার ।

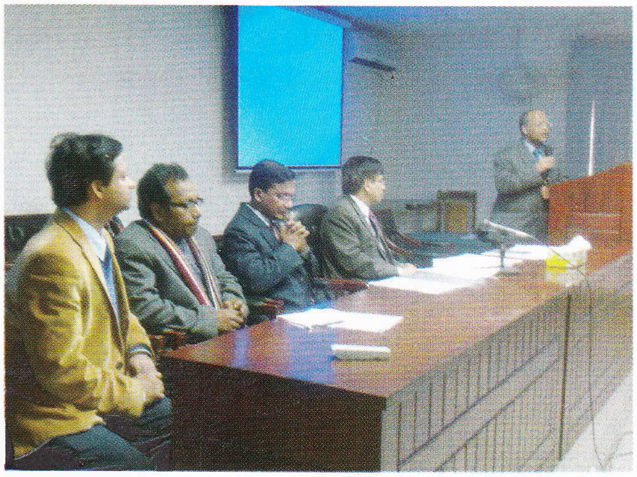

বেইজলাইন সার্ভের তথ্য সংগ্রহকারীদের প্রশিক্ষণ কর্মশালায় আইইডিসিআর এর পরিচালক অধ্যাপক মাহমুদুর রহমান। উপস্থিত নিপসমের পরিচালক অধ্যাপক সরোজ কুমার মজুমদার, অষ্ট্রেলিয়ার নিউ ক্যাসেল ইউনিভার্সিটির অধ্যাপক ও গবেষণা তত্ত্বাবধায়ক ড. মিল্টন হাসনাত, প্রধান গবেষক ডা. ইকবাল কবীরসহ প্রধান অতিথি স্বাস্থ্য ও পরিবার কল্যাণ মন্ত্রণালয়ের সচিব মুহম্মদ হুমায়ুন কবির

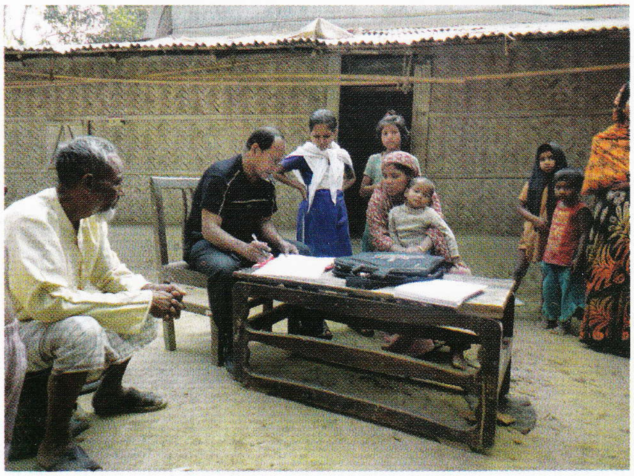

মাঠপর্যায়ে জলবায়ু পরিবর্তন ও স্বাস্থ্য সুরক্ষা প্রকল্পের বেইজলাইন সার্ভের তথ্য সংগ্রহ

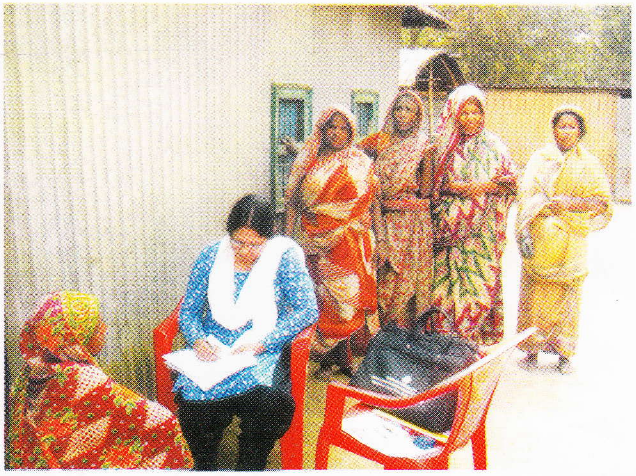

মাঠপর্যায়ে জলবায়ু পরিবর্তন ও স্বাস্থ্য সুরক্ষা প্রকল্পের বেইজলাইন সার্ভের তথ্য সংগ্রহ

অধ্যায় - ৪

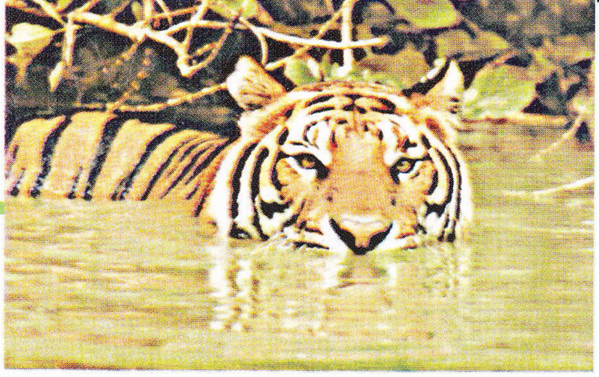

প্রাকৃতিক সম্পদ সংরক্ষণ

সুন্দরবন

সমুদ্র উপকূলবর্তী লোনা পরিবেশে বিশ্বের সবচেয়ে বড় ম্যাংগ্রোভ বনভূমি সুন্দরবন। এই বনভূমি গঙ্গা ও ব্রহ্মপুত্র মোহনায় অবস্থিত এবং বাংলাদেশ ও ভারতের পশ্চিমবঙ্গ জুড়ে বিস্তৃত। দশ হাজার বর্গ কিলোমিটারের ও অধিক জায়গা জুড়ে গড়ে ওঠা সুন্দরবনের ৬,০১৭ বর্গ কিলোমিটার বাংলাদেশে রয়েছে। প্রকৃতপক্ষে সুন্দরবনের আয়তন হওয়ার কথা ছিল প্রায় ১৬,৭০০ বর্গ কি.মি.(২০০ বছর আগের হিসাবে), কমতে কমতে এর বর্তমান আয়তন হয়েছে পূর্বের প্রায় এক-তৃতীয়াংশের সমান। বর্তমানে মোট ভূমির আয়তন ৪,১৪৩ বর্গ কিলোমিটার (বালুতট ৪২ বর্গ কি.মি. এর আয়তনসহ) এবং নদী, খাড়ি খালসহ বাকী জলাধারার আয়তন ১৮৭৪ বর্গ কি.মি। সুন্দরবন ১৯৭০ সালে ইউনেস্কো 'বিশ্ব ঐতিহ্যবাহী স্থান' হিসেবে স্বীকৃতি পায়। সুন্দরবনে জালের মতো জড়িয়ে রয়েছে সামুদ্রিক স্রোতধারা, কাদারচর এবং ম্যাংগ্রোভ বনভূমির ৩১.১ শতাংশ লবণাক্ততাসহ ছোট ছোট দ্বীপ। মোট বন (১৮৭৪ বর্গ কি.মি) জুড়ে রয়েছে নদী-নালা, খাল-বিল মিলিয়ে জলের এলাকা। বনভূমিটি স্বনামে বিখ্যাত। (রয়েল) বেঙ্গল টাইগার ছাড়াও নানান ধরনের পাখি, চিত্রা হরিণ, কুমির ও সাপসহ অসংখ্য প্রজাতি প্রাণির আবাসস্থল হিসেবে পরিচিত।

ভৌগলিক গঠন

দুই প্রতিবেশী দেশ বাংলাদেশ এবং ভারত জুড়ে বিস্তৃত সুন্দরবনের বৃহত্তর অংশটি (৬২%) বাংলাদেশের দক্ষিণ পশ্চিম দিকে অবস্থিত। দক্ষিণে বঙ্গোপসাগর, পূর্বে ধলেশ্বরী নদী আর উত্তরে সীমানা উঁচু এলাকার নদীর প্রধান শাখাগুলো ছাড়া অন্যান্য জলধারাগুলো সর্বত্রই বেড়ীবাধ ও নিচু জমি দ্বারা বহুলাংশে বাধাপ্রাপ্ত। বাংলাদেশের মোট আয়তনের ৪.০৭ শতাংশ জুড়ে রয়েছে সুন্দরবন যা বাংলাদেশ বনবিভাগের আওতাধীন বনাঞ্চলের ৪০ শতাংশ।

সুন্দরবনের নদীগুলো লোনা পানি ও মিঠা পানির মিলন স্থান। এ কারণে গঙ্গা থেকে আসা নদীর মিঠা পানি বঙ্গপোসাগরের লোনা পানি হয়ে ওঠার মধ্যবর্তী স্থান হলো সুন্দরবন এলাকাটি।

জীববৈচিত্র্য

সুন্দরবনের বাস্তুসংস্থান যথেষ্ট জটিল। জৈব উপাদানগুলো এখানে সামুদ্রিক বিষয়ের গঠন প্রক্রিয়া ও প্রাণী বৈচিত্র্যের ক্ষেত্রে গুরুত্বপূর্ণ ভূমিকা রাখে। সৈকত, মোহনা, স্থায়ী ও ক্ষণস্থায়ী জলাভূমি, কাদারচর, খাড়ি, বালিয়াড়ি, মাটির স্তূপের মতো বৈচিত্রময় অংশ গঠিত হয়েছে।

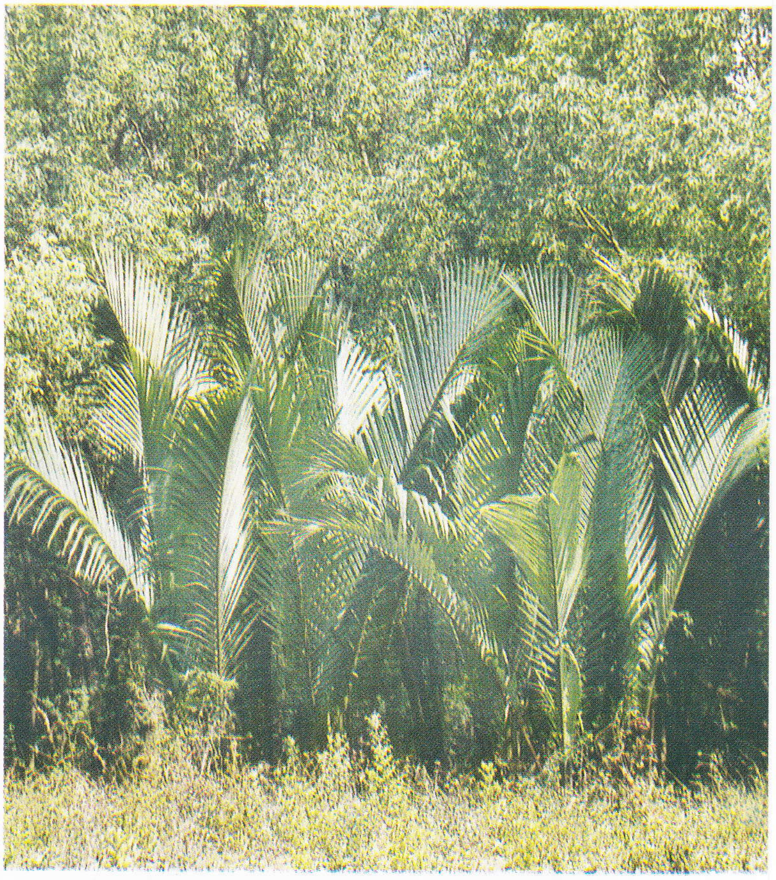

জলবায়ু পরিবর্তনের কারণে সুন্দরবনের গোলপাতা বিপন্নের পথে
ছবি: জাহাঙ্গীর সেলিম

এখানে ম্যাংগ্রোভ উদ্ভিদজগৎ নিজেই নতুন ভূমি গঠনে ভূমিকা রাখে। আবার আন্তঃস্রোতীয় উদ্ভিদজগৎ গুরুত্বপূর্ণ ভূমিকা পালন করে জলজ অঙ্গসংস্থান প্রক্রিয়ায়।

ম্যাংগ্রোভ প্রাণিজগতের উপস্থিতি আন্তঃস্রোতীয় কাদারচরে ব্যষ্টিক অঙ্গসংস্থানিক পরিবেশ তৈরি করে। এটি পলিকে বীজের জন্য আনুভূমিক উপশিলাস্তর সৃষ্টির জন্য ধরে রাখে । এখানে রয়েছে প্রায় ৪০০ প্রজাতির মাছ, ২৭০ প্রজাতির পাখি এবং প্রায় ৩০০ প্রজাতির গাছ। জরিপ অনুযায়ী সুন্দরবনে বাঘের সংখ্যা ৩০০ থেকে ৫০০ এর মধ্যে। কিন্তু এ সংখ্যা কমে আসছে। মানুষের সাথে বাঘের সংঘর্ষ, মানুষ খেকো বাঘের সংখ্যাধিক্য, ফলশ্রুতিতে স্থানীয় জনগণের বাঘের উপর প্রতিশোধপরায়ণ মনোভাব এর অন্যতম কারণ। মূলত খাদ্য সংকট এবং এর পিছনে জলবায়ু পরিবর্তনের প্রভাব অনেক বড় কারণ হিসেবে কাজ করছে।

কক্সবাজার

কক্সবাজার নৈসর্গিক সৌন্দর্যের জন্য বিখ্যাত। এখানে রয়েছে বিশ্বের দীর্ঘতম অভঙ্গুর প্রাকৃতিক বালুময় সমুদ্র সৈকত যা কক্সবাজার শহর থেকে বদরমোকাম পর্যন্ত একটানা ১২০ কি.মি.পর্যন্ত বিস্তৃত এবং চট্রগ্রাম বন্দর

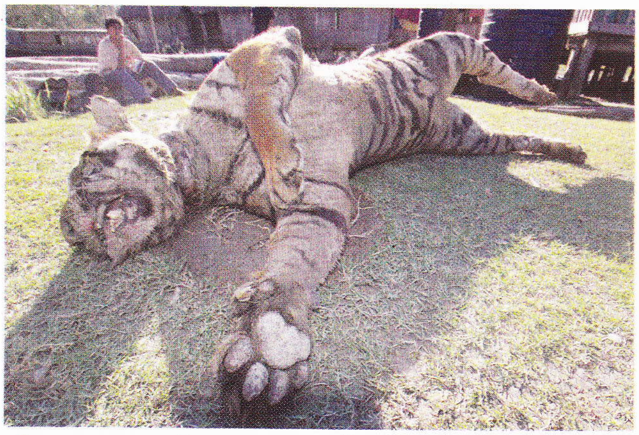

সিডরে সুন্দরবন বিধ্বস্ত হয় এবং জীব বৈচিত্রের ব্যপক ক্ষতি হয়। সিডরে রয়েল বেঙ্গল টাইগারের মৃত্যু। ছবি : জিয়া ইসলাম

থেকে ১৫২ কি.মি. দক্ষিণে অবস্থিত। এটি বাংলাদেশের সবচেয়ে বড় পর্যটন কেন্দ্র। এখানে রয়েছে রূপালি সোনার মতো বালুরাশি আর গ্রীষ্মমণ্ডলীয় বনঘেরা পাহাড়। প্রাকৃতিক খনিজ সম্পদের মধ্যে রয়েছে হমব্লেন্ড, গারনেট, এপিডটিক, ম্যাগনেটাইট, পাইরাইট বিভিন্ন হাইড্রোক্সাইড ইত্যাদি। জলবায়ু পরিবর্তন ও মানুষের অদুরদর্শীতার ফলে আজ আমাদের এই প্রাকৃতিক সম্পদ হুমকীর মুখে। জীববৈচিত্র্য সংরক্ষনে ও জলবায়ূ পরিবর্তনজনিত স্বাস্থ্যঝুকি কমাতে নিজ নিজ অবস্থান থেকে কাজ করার জন্য সকলকে এগিয়ে আসতে হবে।

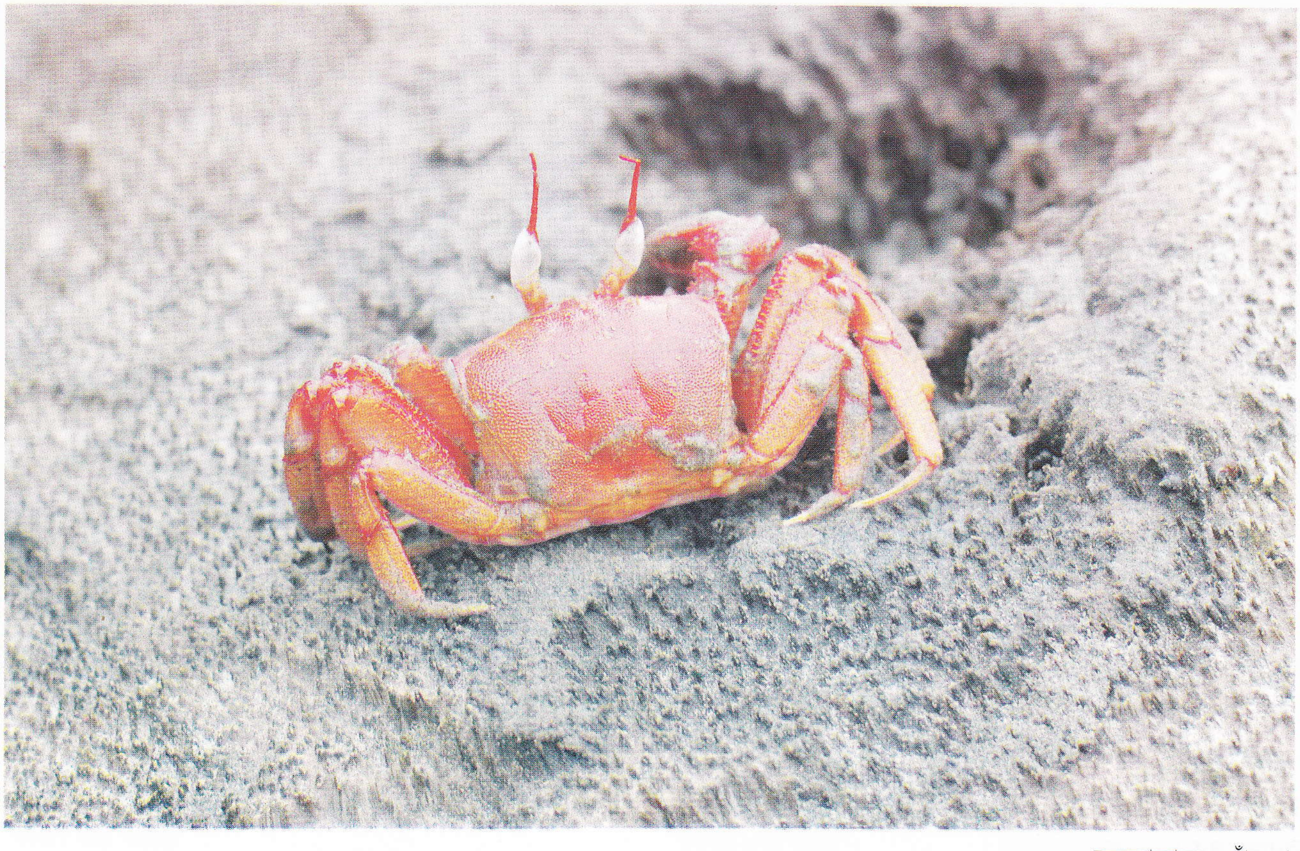

কক্সবাজারের কাঁকড়া

অধ্যায় – ৫

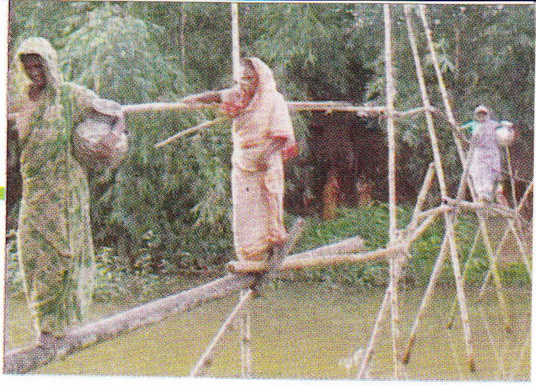

জলবায়ু পরিবর্তন ও স্বাস্থ্য সুরক্ষা

কেস স্টাডি-১

বৃষ্টির পানি সংরক্ষণ

গরমের সময় বেশির ভাগ গ্রামে পানির অভাব দেখা দেয়। গ্রামের বয়স্ক মানুষেরা বলেন যে সম্প্রতি নদী ও জলাশয় শুকিয়ে গেছে। সংরক্ষন ও বৃষ্টির পানি সদ্ব্যবহারের মাধ্যমে বিশুদ্ধ পানি সহজ লভ্য করা যায়। ছাদের উপর বৃষ্টির পানি ধরে রেখে চাষাবাদ করার বিভিন্ন প্রশিক্ষণ দেওয়া হয়েছে মাঠ পর্যায়ে। বিশেষ করে আর্সেনিক দূষণে ক্ষতিগ্রস্ত যে সব অঞ্চলে প্রশিক্ষণ দেওয়া হয় সেসব অঞ্চলে একটি করে জলাধার গড়ে তোলা হয় যাতে গরমকালের জন্য পর্যাপ্ত পানি ধরে রাখা যায়। সরকারিভাবে ঘরে ঘরে ওই জলাধার থেকে পানি নেওয়ার জন্য পাইপলাইন বসানো হয়। স্থানীয় লোকজন সেসব স্থাপনার আংশিক খরচ বহন করে এবং কীভাবে রক্ষণাবেক্ষণ করতে হবে সে বিষয়ে তাদেরকে প্রশিক্ষণ দেওয়া হয়। ছাদের উপরে বৃষ্টির পানি ব্যবহার করে দৈনন্দিন কার্যাবলী সম্পাদন ও চাষাবাদ মানুষের জীবন যাত্রা পাল্টে দিয়েছে এবং জলবায়ু পরিবর্তন জনিত স্বাস্থ্য ঝুঁকি কমাতে বৃষ্টির পানি ব্যবহার একটি কার্যকর উপায় হতে পারে।

কেস স্টাডি-২

জলবায়ু পরিবর্তনের ফলে স্বাস্থ্যের জন্য ক্ষতিকর প্রতিক্রিয়া ভুটান কীভাবে মোকাবিলা করছে

যদিও বিশ্বব্যাপী উষ্ণতা বৃদ্ধি গ্রিণহাউস গ্যাসের নির্গমণের ব্যাপারে ভুটানের অবদান খুবই সামান্য তবুও জলবায়ু পরিবর্তনের ফলে বাংলাদেশের মতো সে দেশটিও বর্তমানে ব্যাপক ক্ষতির সম্মুখীন হচ্ছে। ২০০০ সাল থেকে ভুটানে অস্বাভাবিক দীর্ঘস্থায়ী খরা, অনিয়মিত বৃষ্টিপাতের ধারা এবং বন্যার খবর পাওয়া যায়। অন্তত ২৫টি হিমবাহের হ্রদকে চিহ্নিত করা হয়েছে যা ফেটে পড়ার মতো অবস্থায় আছে। অদুর ভবিষ্যতে হিমবাহ গলা পানির পরিমাণ আরও বেড়ে যাবে। ইন্টারন্যাশনাল সেন্টার ফর ইন্টিগ্রেটেড মাউন্টেন ডেভেলপমেন্ট এর মতে হিমবাহগুলোর ঠাণ্ডা কমে যাওয়ার হার ১৯৯০ সাল থেকে দ্বিগুণ হয়ে ২০ মিটার থেকে ৪০ মিটারে দাড়িয়েছে।

কিছু দিনের মধ্যে দুইটি হ্রদের পানি বিপজ্জনকভাবে কূল ছাপিয়ে আশেপাশের এলাকাকে প্লাবিত করতে পারে। এর একমাত্র প্রতিকার ব্যয়বহুল 'স্পিলওয়ে' বানিয়ে প্রবাহিত নদীর ধারে বসবাসকারী মানুষ এবং ইকোসিস্টেমকে রক্ষা করা। ভুটানে যেহেতু ৮০% ভর্তুকি প্রাপ্ত কৃষিব্যবস্থা সেহেতু জলবায়ু পরিবর্তন প্রত্যক্ষভাবে খাদ্য সুরক্ষায়, স্বাস্থ্য এবং জীবিকা নির্বাহে আঘাত হানবে।

কেস স্টাডি-৩

কীটপতঙ্গবাহী রোগ সচেতনতা

গ্রামের চারপাশে ধানের ক্ষেতে বিভিন্ন ধরনের মশা জন্ম নেয়। শ্রীলংকার শিশুরা জলাশয়ের ধারে মশার লার্ভা দেখছে এবং তারা লার্ভা চিনতে শিখছে। কীটপতঙ্গবাহী রোগের জীবনচক্রটি পানি ও তাপমাত্রার মতো পরিবেশিক পরিস্থিতির গুরুত্ব অনুধাবন করে গ্রামের মানুষেরা সংঘবদ্ধভাবে কীটপতঙ্গনিধন পরিচালনায় পারদর্শী হয়ে উঠে।

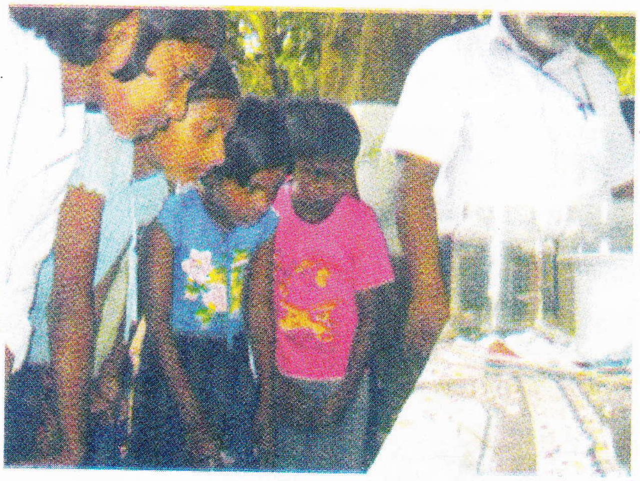

শিশুরা মশার লার্ভা দেখছে এবং চিনতে শিখছে

সমন্বিত কীটপত দমন ব্যবস্থাপনা স্থানীয় জনসাধারণকে জলবায়ু পরিবর্তনের ফলে স্বাস্থ্য সম্পর্কিত প্রতিক্রিয়ার হাত থেকে রক্ষা পেতে এবং সুস্থ থাকতে অবদান রাখে।

পুনরালোচনা

এ সকল গবেষণা থেকে জানা যায়, জলবায়ু পরিবর্তনে পানিবাহিত রোগের বৃদ্ধি বাংলাদেশের স্বাস্থ্য ক্ষেত্রে আরও সমস্যা বয়ে আনবে। এ সকল সমস্যা মোকাবেলা ও জলবায়ু সংবেদনশীল রোগ-বালাই হ্রাসে উপযুক্ত নীতি নির্ধারণ, বৈজ্ঞানিক কর্মপন্থা ও প্রচুর গবেষণার মাধ্যমে প্রাপ্ত বিষয়সমূহ নিশ্চিতকরণ এবং প্রাতিষ্ঠানিক অবকাঠামো গঠন করা প্রয়োজন।

জলবায়ু সংবেদনশীল রোগ এবং ভৌগলিক বিস্তারের উপর নির্ভর করে কীটপতঙ্গের তালিকা করতে হবে। জলবায়ু পরিবর্তন ও এর ভবিষ্যৎ ক্ষতিকর প্রভাব মোকাবেলায় স্বাস্থ্যকর্মীদের প্রশিক্ষণ ও প্রস্তুতি গ্রহণ করতে হবে। জলবায়ু সংক্রান্ত ঝুঁকিগুলো চিহ্নিত করে স্বাস্থ্যের উপর সরাসরি কী কী প্রভাব পড়তে পারে সে বিষয়গুলো গবেষণা করে বের করা জরুরী। ব্যক্তিগত ও দলগতভাবে স্বাস্থ্যসম্মত পরিষ্কার পরিচ্ছন্নতার চর্চা আবশ্যক। পানি সুরক্ষা ও স্যানিটেশন ব্যবস্থার উন্নয়নে কাজ করে যেতে হবে। স্বাস্থ্যের উপর জলবায়ু পরিবর্তনের ক্ষতিকর প্রভাব মোকাবেলায় সকলকে কার্যকর ভূমিকা পালনে উদ্বুদ্ধ করতে হবে।

ক্লাইমেট চেঞ্জ অ্যাণ্ড হেল্‌থ প্রমোশন ইউনিট (সিসিএইচপিইউ)

সাম্প্রতিক কালে জলবায়ু পরিবর্তনের ফলে বাংলাদেশ ব্যাপক ক্ষতির সম্মুখীন হচ্ছে। খরা, বন্যা, দিন দিন আরও প্রকট আকার ধারণ করছে। আইলা এবং সিডর এই ধারাবাহিকতার ছোট্ট দুইটি উদাহরণ। জলবায়ু পরিবর্তনের সাথে যে সমস্ত স্বাস্থ্য সংক্রান্ত সমস্যাগুলো দেশে আরও প্রকট আকার ধারণ করবে তার মধ্যে রয়েছে– অস্বস্তিকর আবহাওয়া, খাদ্য ও পানি বাহিত রোগ (যেমন কলেরা, ডায়রিয়া), বায়ু দূষণের কারণে শ্বাস প্রশ্বাস সংক্রান্ত রোগ, এ্যালার্জি, পানি ও খাদ্য নিরাপত্তা, অপুষ্টি, মনোসামাজিক অবস্থা ইত্যাদি। জলবায়ু পরিবর্তনের ফলে স্বাস্থ্যখাতে উদ্ভূত ঝুঁকিগুলো মোকাবেলায় সিদ্ধান্ত গ্রহণ, সমন্বয়সাধন ও কর্মপরিকল্পনা নির্ধারনের লক্ষ্যে স্বাস্থ্য ও পরিবার কল্যাণ মন্ত্রণালয়ের অধীনে ক্লাইমেট চেঞ্জ অ্যাণ্ড হেল্‌থ প্রমোশন ইউনিট (সিসিএইচপিইউ) নামে একটি আলাদা ইউনিট গঠন করা হয়েছে। জলবায়ু পরিবর্তনের কারণে স্বাস্থ্য সংক্রান্ত বিভিন্ন সমস্যাগুলো পর্যবেক্ষণ, গবেষণা ও কার্যকর পদক্ষেপ গ্রহণ করা, দুর্যোগের সময় জরুরি চিকিৎসা সেবা প্রদান এবং বিদ্যালয়গুলোতে এতদবিষয়ক প্রচারাভিযান চালানো, জলবায়ু পরিবর্তন সংক্রান্ত স্বাস্থ্য বিষয়ক সচেতনতা বৃদ্ধি, সেই সাথে ই-হেল্‌থ ও টেলিমেডিসিন ব্যবস্থার উন্নয়নের লক্ষ্যে এই ইউনিট কাজ করছে। কমিউনিটি ক্লিনিক এবং অন্যান্য প্রাথমিক স্বাস্থ্য পরিচর্যা কেন্দ্রের মাধ্যমে বিদ্যালয় স্বাস্থ্য কার্যক্রমকে আরও জোরদার করে ভবিষ্যৎ প্রজন্মকে জলবায়ু পরিবর্তনে স্বাস্থ্য সুরক্ষার জন্য প্রস্তুত করতে স্বাস্থ্য ও পরিবার কল্যাণ মন্ত্রণালয়ের ক্লাইমেট চেঞ্জ অ্যাণ্ড হেল্‌থ প্রমোশন ইউনিট কাজ করে যাচ্ছে।

সিসিএইচপিইউ, এর প্রকল্পের আওতায় 'বিদ্যালয় স্বাস্থ্য কর্মসূচী ও কমিউনিটি ক্লিনিক স্বাস্থ্য সেবা'র সমন্বয় পূর্বক একটি মডেল তৈরীর কার্যক্রম হাতে নিয়েছে। ভবিষ্যত প্রজন্মকে জলবায়ু পরিবর্তনের স্বাস্থ্য ঝুঁকি মোকাবেলায় যথাযথ ভাবে প্রস্তুত করার অভিপ্রায়ে সিসিএইচপিইউ এই সহায়িকাটি প্রকাশের উদ্যোগ নিয়েছে।

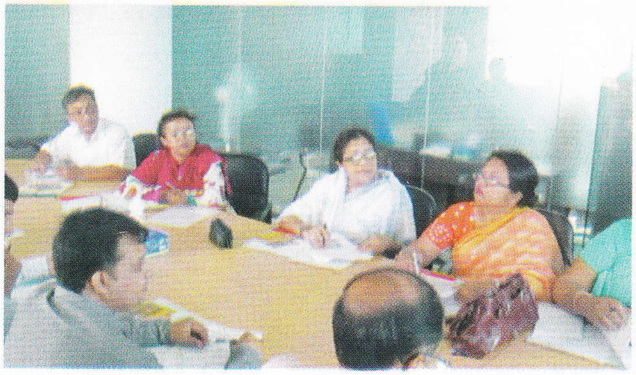

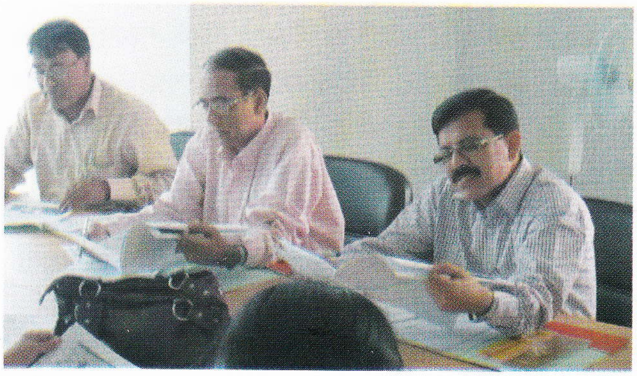

সহায়িকা তৈরির কর্মশালায় এনসিটিবি, পরিবেশ ও বন মন্ত্রণালয়, স্বাস্থ্য ও পরিবার কল্যাণ মন্ত্রণালয়ের প্রকল্প পরিচালক ও অন্যান্য কর্মকর্তাবৃন্দ

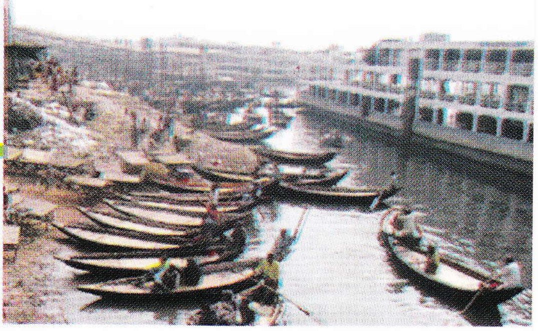

অধ্যায় – ৬

দূষণ কমাই সুস্থ থাকি

দূষণ

বায়ু দূষণ

আমাদের পৃথিবীটাকে ঘিরে যে সুন্দর আর পরিষ্কার বাতাস আছে, যা থেকে মানুষ, পশু-পাখি, গাছ-পালা সবাই নিঃশ্বাস নিয়ে থাকি তা ধীরে ধীরে গাড়ী, কল-কালখানার কালো ধোঁয়া মিশে দূষিত হয়ে পড়ছে। ঢাকা শহরের কিছু কিছু জায়গার বাতাসে এই দূষিত কালো ধোঁয়ার পরিমান এত বেশী যে মানুষজনকে নাক ঢেঁকে চলতে হয়। একেই বলে বায়ু দূষণ। ধোয়া, ধুলাবালি, দুর্গন্ধ ইত্যাদি মিশে বাতাস দূষিত হয়। দূষিত বাতাসের কারণে আমাদের বিভিন্ন অসুখ হয়। গ্রামের বেশীর ভাগ বাড়ীগুলোর ছাদ খুব নীচু হয় আর জানালাগুলোও ছোট থাকে। রাতে প্রায় প্রতিটি বাড়ীর হাঁস-মুরগী, ছাগল ইত্যাদি ঘরে রাখা হয়। এদের গন্ধে ঘরের বায়ু দূষিত হয়। তাছাড়াও অনেক গ্রামে পাকা পায়খানার ব্যবস্থা না থাকায় অনেকেই খোলা মাঠে কাজ সারেন। এর ফলেও বায়ু দূষিত হয়। লাকড়ির চুলায় ভিজা কাঠ, কয়লা, কাগজ, পলিথিন ইত্যাদি ব্যবহারের ফলে প্রচুর বিষাক্ত ধোঁয়া বের হয়। শহরে গাছের সংখ্যা কম কিন্তু মানুষের সংখ্যা অনেক বেশী। তাই এখানে বাতাসে অক্সিজেনের চাইতে কার্বণ-ডাই-অক্সাইডের পরিমান বেড়ে চলেছে। যা বায়ুকে দূষিত করছে। গাড়ীর কালো ধোঁয়াতে কার্বন বের হয় যা মানুষের শরীরের জন্য খুবই ক্ষতিকারক।

মাটি দূষণ

আজকাল চাষ করার সময় প্রচুর রাসায়নিক সার, কীটনাশক ইত্যাদি ব্যবহার করা হয়। এর ফলে মাটি দূষিত হয়। দূষিত মাটিতে ফসল উৎপাদন ক্ষমতা ধীরে ধীরে কমে যায়। এই ফসলের মানও খুব ভাল হয় না। এই সব রাসায়নিক সার ও কীটনাশক বৃষ্টির পানির সাথে সেচের পানির সাথে ধুয়ে নদী, খাল, পুকুরের পানির সাথে মিশে যায়। এর ফলে পানি দূষিত হচ্ছে।

নদী দূষণ

নদীগুলোতে মানুষ ময়লা ফেলছে, আবার শহরের অনেক আবর্জনাও ড্রেনের মধ্যে দিয়ে নদী ও সমুদ্রের পানিতে মিশছে। কল-কারখানার দূষিত রাসায়নিক আবর্জনাও নদীতে ফেলা হচ্ছে এই সব কারণে পানি দূষিত হচ্ছে। সবাই মনে করে যে নদীতো বয়ে চলেছে ময়লাগুলোও বয়ে গিয়ে সাগরের সাথে মিশে যাবে। কিন্তু নদীর চলার পথে আশেপাশে যে সব গ্রাম রয়েছে সেখানকার মানুষরা খাওয়ার পানি পায় নদী থেকে এমনকি অনেকেই কাপড় ধোয়া, গোসল করা সবই এই দূষিত পানি দিয়ে করে। পানি দূষিত হলে এতে বসবাসকারী জীব মরে যায়। দূষিত পানি খেলে ডাইরিয়া, টাইফয়েড ইত্যাদি রোগ হয়। দূষিত পানিতে গোসল করলে বিভিন্ন ধরণের চর্মরোগ হয়। তাই এই সব নদীর পাড়ে বসবাসকারী অনেক মানুষ বিভিন্ন মারাত্মক রোগ-ব্যাধিতে আক্রান্ত হয়।

পানি দূষণ

পৃথিবীতে দিন দিন মানুষ বেড়ে চলেছে। তাই পানির ব্যবহারও বেড়ে চলেছে। মাটির নীচে যে পানি আছে তা মানুষ কল ও টিউবওয়েল দিয়ে অনেক বেশী বেশী করে তুলে নিচ্ছে। কল-কারখানার জন্য, ধোয়া-মোছার বিভিন্ন কাজে দিন দিন মানুষ পানির ব্যবহার বাড়িয়েই চলেছে। আমরা জানি যে পানির আরেকটা নাম হচ্ছে "জীবন"। এভাবে যদি আমরা পানি নষ্ট করতে থাকি তবে কিছুদিন পরে আর পানিই পাবো না। পানি নষ্ট হওয়ার আরেকটা মূল কারণ অপচয়। যেমন আমরা প্রায়ই একটা জিনিস দেখি কিন্তু খেয়াল করে সেটা থামাই না। সেটা কি বলতে পারো? একটু চিন্তা করে দেখ আমাদের প্রত্যেকের বাড়িতে প্রায়ই কল থেকে ফোঁটা ফোঁটা পানি পড়তে থাকে বা হয়তো পাইপের কোণা থেকে ফোঁটা ফোঁটা পানি পড়ে। এটা হয়তো এক মাস হয়ে যায় কেউই খেয়াল করে না। কিন্তু তুমি স্কুলে আসার সময় এই নষ্ট কলের নীচে একটা বালতি বা হাঁড়ি রেখে এসো ফিরে গিয়ে দেখবে এইটুকু সময়েই কত পানি জমেছে।

তাহলে বুঝতেই পারছো আমরা নিজেরাই একটু খেয়াল না করে কত পানি নষ্ট করছি। এভাবে যদি অনেকগুলো বাসাতে এমন নষ্ট কল থাকে তবে প্রতিদিন কত পানি অপচয় হচ্ছে ভাবতে পারো?

শব্দ দূষণ

জোরে শব্দ হলেও পরিবেশ দূষণ ঘটে। যেমন, খুব জোরে গাড়ীর হর্ণ বাজালে বা বোমা ফাটালে। একে শব্দ দূষণ বলে। শব্দ দূষণ হলে মাথা ব্যাথা হয়, কাজ করতে অনিচ্ছা হয়, তাছাড়াও নানা অসুখ হয়। আমরা বাড়ীর আশেপাশে, রাস্তাঘাটে, কাগজ, ময়লা, বাদামের খোসা ইত্যাদি ফেলেও পরিবেশ দূষণ করি।-

পৃথিবীতে পানি

নদীর পানিতে যেমন বাঁধ দিয়ে বিদ্যুৎ তৈরী করা যায় তেমনই নদী আমাদের যাতায়াতের রাস্তাও বটে। আমাদের দেশের অনেক গ্রামে পৌঁছাতে হলে নৌকায় করে নদী পথে যেতে হয়। আবার সমুদ্র পথে জাহাজে করে এক দেশ থেকে অন্য দেশে জিনিস-পত্র আনা নেওয়া করা হয়। পানি আমাদের জন্য এত প্রয়োজনীয় হলেও পৃথিবীতে যে পরিমান পানি আছে তার কিন্তু মাত্র ১০০ ভাগের ১ ভাগ আমাদের ব্যবহার করার উপযোগী। কারণ বাকী ৯৯ ভাগ যে পানি আছে তার বেশীর ভাগ রয়েছে সমুদ্রের লোনা পানি হিসাবে, আর কিছুটা শীতের দেশগুলোতে বা উঁচু পাহাড়ের উপরে বরফ বা হিমবাহ হিসাবে জমে রয়েছে। সুতরাং সমুদ্রের তলার প্রাণী ও উদ্ভিদ ছাড়া পৃথিবীর অন্যান্য সকল প্রাণী ও উদ্ভিদ কিন্তু মাত্র ১ ভাগ পানির উপর নির্ভর করে বেঁচে আছে। আমরা কথায় বলি যে পৃথিবীর ৩ ভাগ জল আর ১ ভাগ স্থল। পৃথিবীর মোট পানির ১০০ ভাগের ৯৭ ভাগ পানি রয়েছে সমুদ্রে। এই সম্পূর্ণ পানিই লোনা যা মানুষ বিশেষ ব্যবহার করতে পারে না। কারণ লোনা পানি থেকে লবন তুলে মিঠা পানি তৈরী করতে হলে অনেক টাকার প্রয়োজন এবং এটা করা খুব সহজও নয় বাকী যে ৩ ভাগ পানি আছে সেটা মিঠা বা স্বাদু পানি যা মানুষের ব্যবহারের উপযোগী। কিন্তু এই মিঠা পানিরও ১০০ ভাগের ৭৫ ভাগ মিঠা পানিই শীতের দেশগুলোতে ও উঁচু পাহাড়ের চুড়ায় বরফ আর হিমবাহ হিসাবে আটকে আছে। মাত্র বাকী ২৫ ভাগ পানি মাটির নীচে, নদী-নালা, খাল-বিল, পুকুর ইত্যাদিতে রয়েছে।

একটি তালিকাতে পৃথিবীর কোথায় কত পানি আছে তার হিসাব দেওয়া হলো ঃ

মহাসাগর	৯৭.২%
বরফ ও হিমবাহ	০২.০%
মাটির নীচের পানি	০০.৬২%
খাল-বিল	০০.০০৯%
সাগর ও হ্রদ	০০.০০৮%
বায়ুমন্ডলে জলীয় বাষ্প	০০.০০১%
নদী	০০.০০০১%
**পৃথিবীর মোট পানি**	**৯৯.৮৩৮১%**

সামান্য যে পরিমান পানি হিসাবের বাইরে আছে মনে করা হয় যে সেটুকু পানি চক্রে ব্যস্ত আছে। আমরা তো এবার জানলাম আমাদের ব্যবহারের জন্য কত অল্প পানি পৃথিবীতে আছে। আমরা প্রতিদিন বিভিন্ন কাজে পানি ব্যবহার করি। যেমন পানি খাই, ক্ষেতের ফসলে পানি সেচ দেই, ফুল ও ফল গাছে পানি দেই, কাপড় ধুই, বাসন-পত্র ধুই, ঘর মুছি, গোসল করি আবার পুকুর ও নদী থেকে মাছ ধরি। সুতরাং দেখা যাচ্ছে আমাদের প্রতিটি কাজে পানির খুবই প্রয়োজন। আমরা অনেক সময় খেয়াল করি না তাই এই সামান্য পরিমান পানিরও যত্ন নেই না। আমরা পানিতে ময়লা ফেলি, কল-কারখানার আবর্জনা ফেলি, ক্ষেতে যে রাসায়নিক সার ও কীটনাশক দিই তাও পানিতে মিশে। এভাবে পানি দূষিত হয়। এতে নদীর মাছ মরে যায়। দূষিত পানি ও দূষিত মাছ খেয়ে আমাদের শরীর খারাপ হয়। তাই আমাদের সবার কর্তব্য এই মূল্যবান পানি পরিষ্কার ও নিরাপদ রাখা।

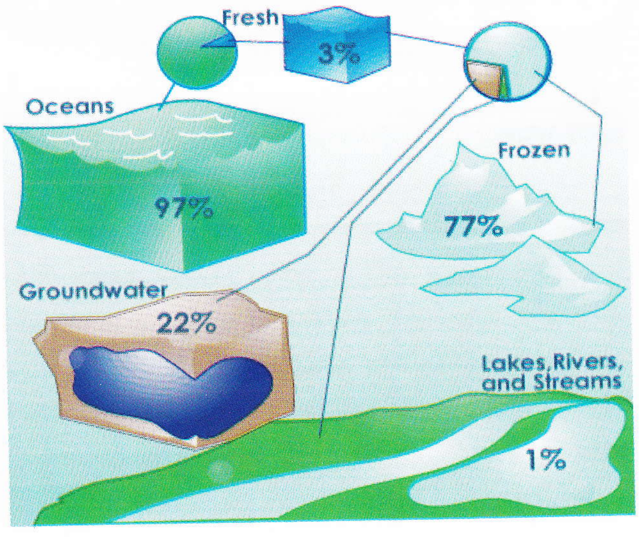

অধ্যায় – ৭

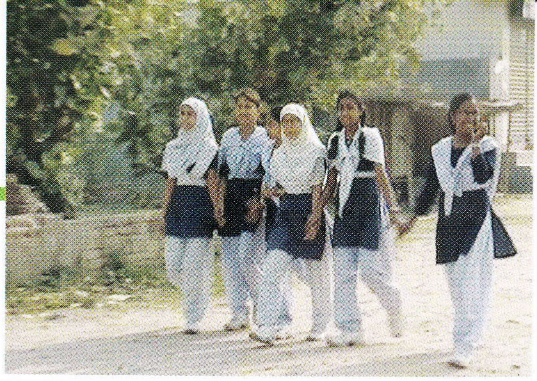

শিক্ষার্থীদের জন্য ৩ দিনের পাঠ পরিকল্পনা

ক্রিয়াকলাপ : প্রথম দিন

নিচের দ্রব্যগুলো তোমার ব্যবহার শেষে কী করবে, সঠিক উত্তরের পাশে টিক (✓) চিহ্ন দাও –

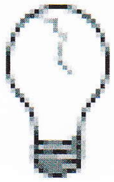

- ☐ রিসাইকেল করানো
- ☐ জৈব সার হিসেবে ব্যবহার
- ☐ যার প্রয়োজন তাকে দিয়ে দেওয়া, রিইউজ করা
- ☐ আবর্জনা হিসেবে ফেলে দেওয়া

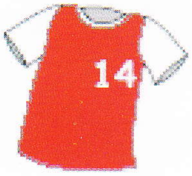

- ☐ রিসাইকেল করানো
- ☐ জৈব সার হিসেবে ব্যবহার
- ☐ যার প্রয়োজন তাকে দিয়ে দেওয়া, রিইউজ করা
- ☐ আবর্জনা হিসেবে ফেলে দেওয়া

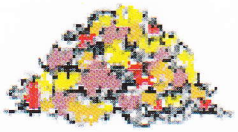

- ☐ রিসাইকেল করানো
- ☐ জৈব সার হিসেবে ব্যবহার
- ☐ যার প্রয়োজন তাকে দিয়ে দেওয়া, রিইউজ করা
- ☐ আবর্জনা হিসেবে ফেলে দেওয়া

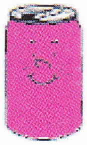

- ☐ রিসাইকেল করানো
- ☐ জৈব সার হিসেবে ব্যবহার
- ☐ যার প্রয়োজন তাকে দিয়ে দেওয়া, রিইউজ করা
- ☐ আবর্জনা হিসেবে ফেলে দেওয়া

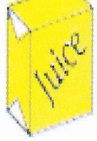

- ☐ রিসাইকেল করানো
- ☐ জৈব সার হিসেবে ব্যবহার
- ☐ যার প্রয়োজন তাকে দিয়ে দেওয়া, রিইউজ করা
- ☐ আবর্জনা হিসেবে ফেলে দেওয়া

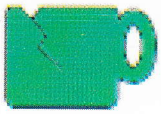

- ☐ রিসাইকেল করানো
- ☐ জৈব সার হিসেবে ব্যবহার
- ☐ যার প্রয়োজন তাকে দিয়ে দেওয়া, রিইউজ করা
- ☐ আবর্জনা হিসেবে ফেলে দেওয়া

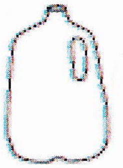

☐ রিসাইকেল করানো
☐ জৈব সার হিসেবে ব্যবহার
☐ যার প্রয়োজন তাকে দিয়ে দেওয়া, রিইউজ করা
☐ আবর্জনা হিসেবে ফেলে দেওয়া

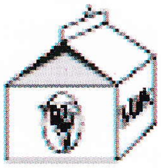

☐ রিসাইকেল করানো
☐ জৈব সার হিসেবে ব্যবহার
☐ যার প্রয়োজন তাকে দিয়ে দেওয়া, রিইউজ করা
☐ আবর্জনা হিসেবে ফেলে দেওয়া

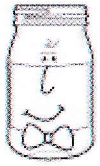

☐ রিসাইকেল করানো
☐ জৈব সার হিসেবে ব্যবহার
☐ যার প্রয়োজন তাকে দিয়ে দেওয়া, রিইউজ করা
☐ আবর্জনা হিসেবে ফেলে দেওয়া

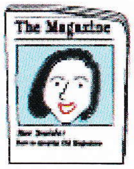

☐ রিসাইকেল করানো
☐ জৈব সার হিসেবে ব্যবহার
☐ যার প্রয়োজন তাকে দিয়ে দেওয়া, রিইউজ করা
☐ আবর্জনা হিসেবে ফেলে দেওয়া

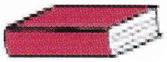

☐ রিসাইকেল করানো
☐ জৈব সার হিসেবে ব্যবহার
☐ যার প্রয়োজন তাকে দিয়ে দেওয়া, রিইউজ করা
☐ আবর্জনা হিসেবে ফেলে দেওয়া

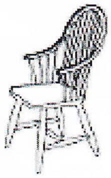

☐ রিসাইকেল করানো
☐ জৈব সার হিসেবে ব্যবহার
☐ যার প্রয়োজন তাকে দিয়ে দেওয়া, রিইউজ করা
☐ আবর্জনা হিসেবে ফেলে দেওয়া

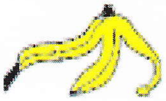

☐ রিসাইকেল করানো
☐ জৈব সার হিসেবে ব্যবহার
☐ যার প্রয়োজন তাকে দিয়ে দেওয়া, রিইউজ করা
☐ আবর্জনা হিসেবে ফেলে দেওয়া

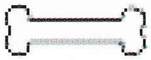

☐ রিসাইকেল করানো
☐ জৈব সার হিসেবে ব্যবহার
☐ যার প্রয়োজন তাকে দিয়ে দেওয়া, রিইউজ করা
☐ আবর্জনা হিসেবে ফেলে দেওয়া

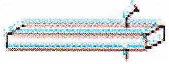

☐ রিসাইকেল করানো
☐ জৈব সার হিসেবে ব্যবহার
☐ যার প্রয়োজন তাকে দিয়ে দেওয়া, রিইউজ করা
☐ আবর্জনা হিসেবে ফেলে দেওয়া

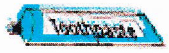

☐ রিসাইকেল করানো
☐ জৈব সার হিসেবে ব্যবহার
☐ যার প্রয়োজন তাকে দিয়ে দেওয়া, রিইউজ করা
☐ আবর্জনা হিসেবে ফেলে দেওয়া

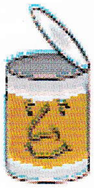

☐ রিসাইকেল করানো
☐ জৈব সার হিসেবে ব্যবহার
☐ যার প্রয়োজন তাকে দিয়ে দেওয়া, রিইউজ করা
☐ আবর্জনা হিসেবে ফেলে দেওয়া

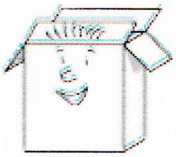

☐ রিসাইকেল করানো
☐ জৈব সার হিসেবে ব্যবহার
☐ যার প্রয়োজন তাকে দিয়ে দেওয়া, রিইউজ করা
☐ আবর্জনা হিসেবে ফেলে দেওয়া

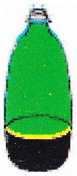

☐ রিসাইকেল করানো
☐ জৈব সার হিসেবে ব্যবহার
☐ যার প্রয়োজন তাকে দিয়ে দেওয়া, রিইউজ করা
☐ আবর্জনা হিসেবে ফেলে দেওয়া

তোমার উত্তরগুলো শিক্ষককে দেখাও এবং সঠিক উত্তরগুলো জেনে নাও। সঠিক উত্তরগুলো শিক্ষকদের জন্য প্রণীত সহায়িকাটিতে দেওয়া আছে।

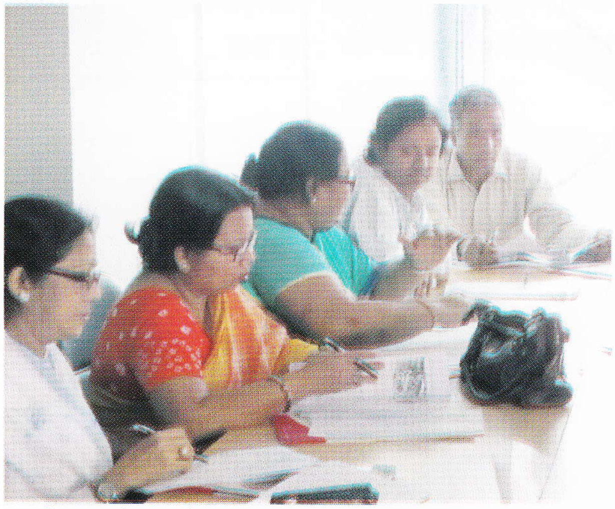

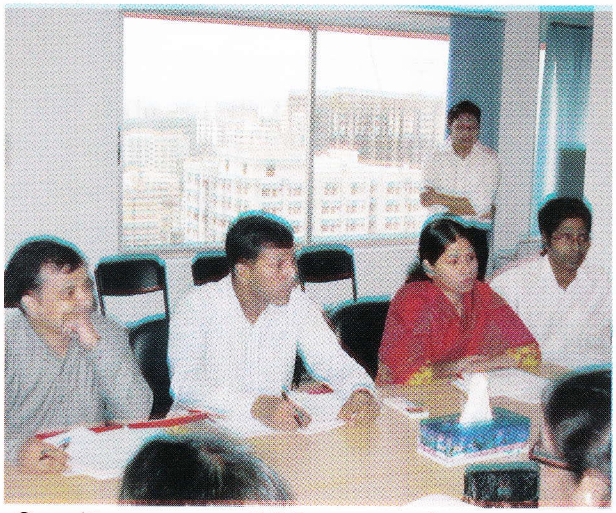

সহায়িকা তৈরির কর্মশালায় স্বাস্থ্য ও পরিবার কল্যাণ মন্ত্রণালয়ের যুগ্ম সচিব শফিকুল ইসলাম লস্কর, এনসিটিবির সদস্য (কারিকুলাম) প্রফেসর তাহেরা আখতার জাহান, প্রফেসর ড. পারভিন সুলতানা, শাহিনারা বেগম এবং পরিবেশ ও বন মন্ত্রণালয়ের ক্লাইমেট চেঞ্জ ইউনিটের কর্মকর্তাবৃন্দ

খেলতে খেলতে শেখা

উদ্দেশ্য	ঃ পৃথিবীর মোট পানির কতটুকু মানুষ ব্যবহার করতে পারে তার সম্পর্কে ধারণা পাওয়া
দলের সদস্য	ঃ ১০ থেকে ৩০ জন
সময়	ঃ ১০ থেকে ১৫ মিনিট
স্থান	ঃ যে কোন জায়গায় যেখানে ক্লাসের সবাই একত্রে গোল হয়ে বসতে পারে
কি কি লাগবে	ঃ একটি মাঝারি খালি বালতি, দুইটি বাটি (স্বচ্ছ যা বাইরে থেকে দেখা যায়), একটি চায়ের চামচ, একটি বিকার অথবা ২৫০ মিলি খালি বোতল, পানি, একটি ড্রপার (যদি পাওয়া যায়)

খেলা (শিক্ষক / শিক্ষিকার প্রতি নির্দেশনা)

১। ছাত্র-ছাত্রীদেরকে গোল হয়ে বসতে বলুন। বলুন যে একটি সহজ পরীক্ষা করলেই আমরা পৃথিবীর পানি সম্পর্কে ধারনা পেতে পারি।

২। সবার বৃত্তের মাঝে বালতিটি রাখুন। ওদেরকে বলুন যে এই বালতিতে ২২০০ মি.লি. লিটার পানি ভরতে হবে। ছোট পেট বোতলগুলোতে মোটামোটি ২৫০ মি.লি. পানি ধরে। সুতরাং এই বোতলের সাড়ে আট বোতল পানি নিলে আনুমানিক ২২০০ মি.লি. পানি হবে। একজন বা দুইজন ছাত্র/ছাত্রীকে দায়িত্ব দিন তারা সাড়ে আট বোতল পানি এনে বালতিতে ঢালবে। সবাইকে বলুন যে এই বালতিতে যে পানি আছে মনে করি এটা পৃথিবীতে মোট পানির পরিমান। অন্য একজন ছাত্র/ছাত্রীকে বলুন যে এই বালতির পানি থেকে একটি চায়ের চামচ দিয়ে ১২ চামচ পানি কাঁচের একটি বাটিতে রাখতে। এবার সবাইকে বলুন বাটিতে যতটুকু পানি আছে মাত্র ততটুকট স্বাদু বা মিঠা পানি পৃথিবীতে আছে। এই পানি আছে নদী-নালা, খাল-বিল, পাহাড় চুড়ার বরফ এবং মাটির নীচের পানি হিসাবে। আর বালতিতে যে পানি থাকলো তা হচ্ছে সাগর আর মহাসাগরের লোনা পানির পরিমান। ছাত্র/ছাত্রীকে বলুন এই বাটি থেকে আড়াই চামচ পানি নিয়ে অপর একটি বাটিতে রাখতে। এবার বলুন আগের বাটিতে যে সাড়ে ৯ চামচ পানি রইলো ততটুকু পরিমান স্বাদু পানি রয়েছে মাটির নীচের পানি, পুকুর, খাল-বিলের পানি হিসাবে। এবার যে বাটিতে আড়াই চামচ পানি রয়েছে তা থেকে আধা ড্রপার পানি ড্রপারে রাকুন অথবা আধা চা চামচ পানি একটি চা চামচে রাখুন। এবার বলুন ড্রপারের অথবা চা চামচের পানিটুকু হচ্ছে নদরি পানি আর বাটিতে যে দুই চামচ পানি রইলো তা হচ্ছে পাহাড়ের চুড়ার বরফ। এবার ছাত্র/ছাত্রীদেরকে প্রতিটি জায়গায় রাখা পানির পরিমান তুলনা করে অনুমান করতে বলুন এবং দেখতে বলুন যে মাত্র কতটুকু পানি মানুষ তার খাওয়া বা ব্যবহারের জন্য পায় অর্থাৎ এইটুকু পানি দিয়েই খাওয়া, ঘর মোছা, কাপড় ধোয়াসহ সব কাজ করতে হয়।

৩। ফ্লিপ চার্টের সাহায্য নিয়ে পানি চক্র সম্পর্কে বুঝিয়ে বলুন। পৃথিবীতে মিঠা পানির পরিমান যে কত কম তা বুঝিয়ে বলুন। পানি ও দূষণ ও অপচয়ের ব্যাপারে সবাইকে সতর্ক হতে বলে আলোচনা শেষ করুন।

এই খেলা থেকে আমরা কী শিখলাম ঃ

পৃথিবীর সব জায়গায় বিভিন্ন অবস্থায় পানি রয়েছে। এ পানি চক্রাকারে পৃথিবীতে ঘুরতে থাকে। পানি না হলে কোন প্রাণী বা গাছ-পালা বাঁচতে পারে না। পৃথিবীর তিন ভাগ পানি এক ভাগ স্থল। আবার এই এক ভাগ পানির মাত্র তিন ভাগ মিঠা পানি। ফলে আমাদের ব্যবহারের জন্য মিঠা পানির পরিমান খুবই কম। আমরা যদি পানি অপচয় করি, পানি দূষিত করি তবে একদিন আমরা আর ভাল পানি পাবো না। আমাদেরকে পানির জন্য কাঁদতে হবে। আমাদের সবার কর্তব্য পানি সম্পদ রক্ষা করা।

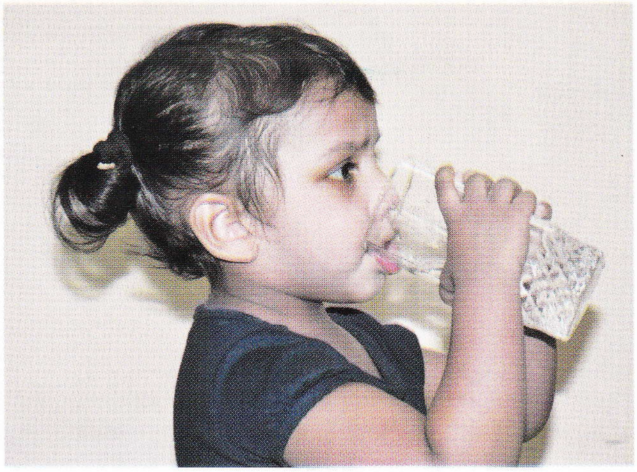

বাতাসের খেলা

খেলতে খেলতে শেখা

উদ্দেশ্য	ঃ বায়ু দূষণ সম্পর্কে ধারনা পাওয়া
দলের সদস্য	ঃ ১৫ থেকে ২০ জন
সময়	ঃ ১০ থেকে ১৫ মিনিট
স্থান	ঃ ক্লাসের বাইরে রাস্তার ধারে খোলা জায়গা
কি কি লাগবে	ঃ একটুকরা সাদা কাগজ, ভেজলিন বা পমেড

খেলা (শিক্ষক / শিক্ষিকার প্রতি নির্দেশনা)

১। ছাত্র-ছাত্রীদেরকে ৪টি দলে ভাগ করুন। প্রতিটি দল একটি সাদগা কাগজ নেবে। কাগজে ভেজলিন বা পমেড লাগিয়ে রাস্তার ধারে অথবা স্কুলের আশেপাশে যদি কোন কলকারখানা থাকে তার কাছে অথবা ক্ষেতে যেখানে খড় পোড়ানো হচ্ছে তা কাছে রাখবে। এমনভাবে কিছু চাপা দিয়ে রাখবে যাতে কাগজটা উড়ে না যায় কিন্তু ভেজলিন লাগানো অংশটা খোলা থাকবে।

২। ১৫ মিনিট পর দলের একজন গিয়ে কাগজটা নিয়ে আসবে। দেখা যাবে কাগজটা কালো হয়ে গেছে। ভেজলিন বা পমেডের সাথে ময়লা আটকে আছে।

৩। ছাত্র-ছাত্রীদের বুঝিয়ে বলুন যে শ্বাস নেওয়ার সময় এই রকম কালো ধোঁয়া আমাদের দেহে প্রবেশ করে এবং ধীরে ধীরে তা আমাদের শরীরের ক্ষতি করে।

এই খেলা থেকে আমরা কী শিখলাম ঃ
পরিবেশে সব সময়ই ক্ষতিকারক পদার্থ মিশে তাকে দূষিত করছে। গাছ কাটা, অতিরিক্ত কল-কারখানা তৈরী, মাটির চুলা ও যানবাহনের কালো ধোঁয়া, যেখানে ময়লা ফেলা ইত্যাদি কারণে বিভিন্নভাবে আমরা পরিবেশ দূষণ দেখতে পাই। যেমন বায়ু দুষণ, পানি দূষণ, মাটি দূষণ, শব্দ দূষণ ইত্যাদি। আমাদের কর্তব্য এই দূষণের পরিমান কমানো ও পরিবেশকে রক্ষা করা।

অক্সিজেন ও কার্বন-ডাই-অক্সাইডের খেলা

খেলতে খেলতে শেখা

উদ্দেশ্য	ঃ বাতাস এবং বাতাসে অক্সিজেন ও কার্বন-ডাই-অক্সাইডের উপস্থিতি সম্পর্কে ধারণা পাওয়া
দলের সদস্য	ঃ ২০ থেকে ২৫ জন
সময়	ঃ ৫ থেকে ১০ মিনিট
স্থান	ঃ ক্লাসের ভিতরে
কি কি লাগবে	ঃ একটি মোমবাতি, একটি দিশাইলাই একটি কাঁচের গ্লাস যা মোমবাতিটির চেয়ে আকারে বড়, একটি মোমদানি

খেলা (শিক্ষক / শিক্ষিকার প্রতি নির্দেশনা)

১। ক্লাসের নিজেদের জায়গায় বসতে বলুন। এই খেলাটি অপেক্ষাকৃত ছোট ক্লাসের জন্য। সবাই দেখতে পারে এমন জায়গায় টেবিলের উপর মোমদানিতে মোমবাতিটি লাগান। এবার ছাত্র-ছাত্রীদের বলুন অক্সিজেন ছাড়া কোন প্রাণী বাঁচতে পারে না, অক্সিজেন ছাড়া আগুনও জ্বলতে পারে না। এটি আমরা পরীক্ষা করে দেখব।

২। এবার মোমবাতিটি জ্বালান। গ্লাস দিয়ে মোমবাতিটি ঢেকে দিন। ছাত্র-ছাত্রীদের দেখতে বলুন, কিছুক্ষণ পর মোমবাতিটি নিভে যাবে।

৩। সবাইকে বুঝিয়ে বলুন গ্লাসের ভিতর যেটুকু অক্সিজেন ছিল তা শেষ হয়ে যাওয়ায় মোমবাতিটি নিভে গেল। আর মোমবাতিটি পোড়ার সময় কার্বন-ডাই-অক্সাইড নির্গত হয়েছে। মানুষ নিঃশ্বাসের সাথে অক্সিজেন আর কার্বন-ডাই-অক্সাইড ত্যাগ করে। বায়ুমন্ডলে কার্বন-ডাই-অক্সাইডের পরিমাণ বেড়ে গেলে অক্সিজেনের পরিমাণ কমে যায়। ফলে শ্বাস নিতে কষ্ট হয়।

এই খেলা থেকে আমরা কী শিখলাম ঃ
বাতাস হচ্ছে বিভিন্ন রকম গ্যাসের মিশ্রণ। প্রতিটি প্রাণী বাতাস হতে নিঃশ্বাসের সাথে অক্সিজেন নেয় ও কার্বন-ডাই-অক্সাইড ত্যাগ করে। গাছ-পালা নিঃশ্বাসের সাথে কার্বন-ডাই-অক্সাইড নেয় ও অক্সিজেন ত্যাগ করে। প্রাণী ও বৃক্ষের মধ্যে এভাবেই গ্যাসের ভারসাম্য রক্ষা হয়। বায়ুমন্ডল সূর্যের অতিরিক্ত তাপ পৃথিবীতে আসতে বাধা দেয়। তাই বায়ুমন্ডল এবং গাছ-পালা আমাদের জন্য, বিশেষত্ব অক্সিজেনের জন্য খুবই দরকারি এবং উপকারী।

অক্সিজেন ও কার্বন-ডাই-অক্সাইডের খেলা

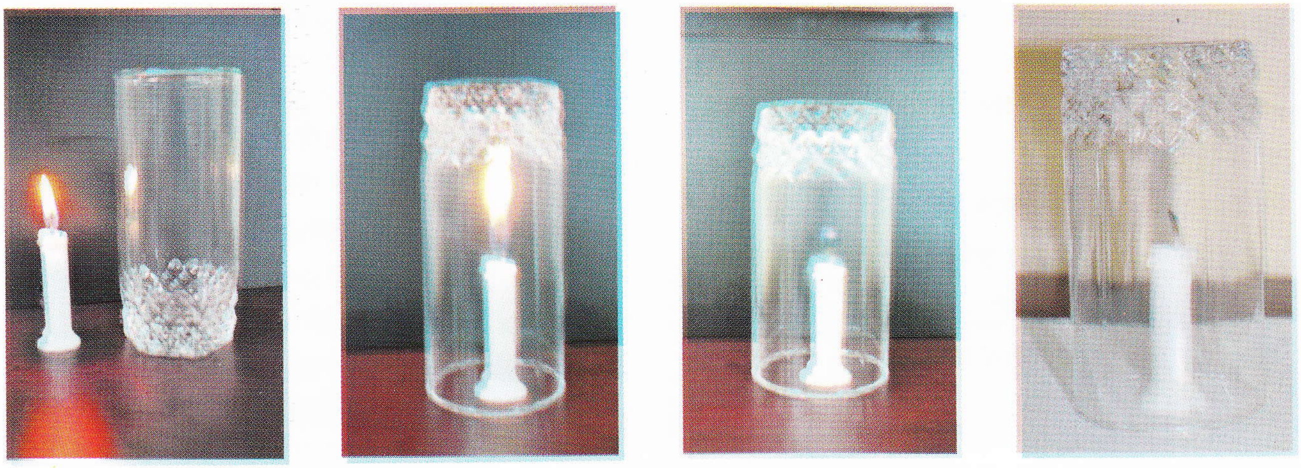

নিজেকে জানো, এসো আচরণ পরিবর্তন করি

প্রতিদিন আমি যা করি বা তুমি যা করো –

তালিকা	সবসময়	কখনো কখনো	কখনোই নয়
ঘর থেকে বের হওয়ার সময় প্রতিবার বাতিটি নিভিয়ে যাও ?			
বাজারে যাওয়ার সময় বাড়ি থেকে চট, কাপড় অথবা কাগজের ব্যাগ নিয়ে যাও ?			
কাগজের উভয় পৃষ্ঠা লেখার জন্য ব্যবহার কর ?			
পুরোনো কাপড় অন্যকে ব্যবহারের জন্য দিয়ে দাও ?			
অব্যবহৃত বা অপ্রয়োজনীয় জিনিস দান করে দাও ?			
হাত মুখ ধোয়ার সময় পানির কল বন্ধ রাখো ?			
পুরানো খেলনাগুলো অন্য শিশুদের খেলার জন্য দিয়ে দাও ?			
অপ্রয়োজনীয় জিনিসগুলো সংগ্রহ করে শিল্প বস্তু তৈরি কর ?			
জৈব বর্জ্যগুলো একত্র করে সার হিসেবে কাজে লাগাও ?			

শিক্ষার্থীদের জন্য বুদ্ধির খেলা

১. জলবায়ু পরিবর্তন কী ? (৫মিনিট)

২. কেন এটি হয় ? (৫ মিনিট)

৩. আমাদের উপরে এর কী কী প্রভাব পড়ে ? (৫ মিনিট)

৪. স্বাস্থ্যের উপর জলবায়ুর সবচেয়ে গুরুত্বপূর্ণ প্রভাব কী ? (১০ মিনিট)

ক্রিয়াকলাপ : দ্বিতীয় দিন

৬ষ্ঠ ও ৭ম শ্রেণি

প্রত্যেককে জলবায়ু পরিবর্তন স্বাস্থ্যের উপর এর প্রভাব বিষয়ক ছবি আঁকতে হবে অথবা মডেল তৈরী করবে। স্বাস্থ্য সমস্যাগুলোর মাঝে থাকতে পারে নিম্নোক্ত রোগগুলো:

১. শ্বাস-প্রশ্বাসজনিত অসুখগুলো এবং/অথবা

২. পুষ্টির অভাব এবং/অথবা

৩. আঘাত এবং/অথবা

৪. উদরঘটিত রোগ এবং/অথবা

৫. ডেঙ্গু এবং ম্যালেরিয়া এবং/অথবা

৬. দুর্যোগকালে মনোসামাজিক চাপ

৮ম, ৯ম, ১০ম শ্রেণি

জলবায়ু পরিবর্তন ও স্বাস্থ্য সুরক্ষা বিষয়ক প্রকল্প বা দেয়াল পত্রিকা প্রকাশ করতে হবে। এজন্য চার থেকে ছয় জন ছাত্র-ছাত্রী মিলে এক একটি দল গঠন করবে।

দেয়াল পত্রিকা ঃ জলবায়ু পরিবর্তনের উপর একটি ছবি এঁকে তার সাথে সংযুক্ত করতে হবে, এজন্য চার থেকে ছয় জন ছাত্র-ছাত্রী মিলে একটি দল গঠন করবে। বিষয়গুলোর মধ্যে অন্তর্ভুক্ত থাকতে পারে–

১. শ্বাস-প্রশ্বাসজনিত অসুখগুলো এবং/অথবা

২. পুষ্টির অভাব এবং/অথবা

৩. আঘাত এবং/অথবা

৪. উদরঘটিত রোগ এবং/অথবা

৫. ডেঙ্গু এবং ম্যালেরিয়া এবং/অথবা

৬. সাইকোসোশাল স্ট্রেস

৭. জনস্বাস্থ্যের উপর জলবায়ুর পরিবর্তনের প্রভাব

৮. স্থানীয় কমিউনিটি ক্লিনিক ও স্কুল স্বাস্থ্য কর্মসূচী

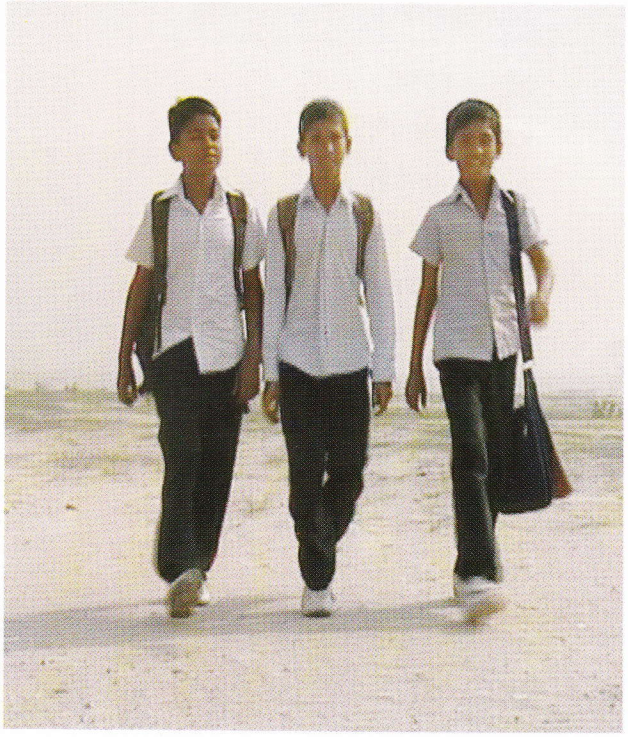

ক্রিয়াকলাপ : তৃতীয় দিন

জলবায়ু পরিবর্তনের সাথে সম্পর্কিত স্বাস্থ্য সমস্যা

ওয়ার্কশিট

কীটপতঙ্গের (ভেক্টর) সাথে রোগ মিলিয়ে দেখা

রোগ	ভেক্টর
১. ডেঙ্গুজ্বর	১. এডিস মশা
২. জাপানিজ এনকেফালাইটিস	২. কিউলেক্স মশা
৩. ম্যালেরিয়া	৩. স্যাণ্ড ফ্লাইস বা বেলে মাছি
৪. ইয়েলো ফিভার	৪. অ্যানোফিলিস মশা
৫. কালাজ্বর	৫. কিউলেক্স মশা

কোনটি পানিবাহিত রোগ ও কোনটি কীটপতঙ্গবাহিত রোগ–

রোগের নাম	পানিবাহিত	কীটপতঙ্গবাহিত
১. কলেরা		
২. হেপাটাইটিস		
৩. ডায়রিয়া		
৪. কালাজ্বর		
৫. ম্যালেরিয়া		
৬. ফাইলেরিয়া		
৭. টাইফয়েড		
৮. নিউমোনিয়া		
৯. ডেঙ্গু		
১০. চিকুনগুণিয়া		

তোমার উত্তরগুলো শিক্ষককে দেখাও এবং সঠিক উত্তরগুলো জেনে নাও। সঠিক উত্তরগুলো শিক্ষকদের জন্য প্রণীত সহায়িকাটিতে দেওয়া আছে।

শিক্ষার্থীদের জন্য শব্দজট

১.				২.		৩.
৪.	৫.				১০.	
	৬.	৭.	৮.	৯.		
	১১.				১২.	
১৩.			১৪.			১৫.
		১৬.			১৭.	
১৮.	১৯.		২০.			

উপর-নীচ :

৩. পর্বতমালা
৪. আবহাওয়ার সমষ্টিগত গড়, যা বেশ কয়েক বছর ধরে চলে
৬. যার আরেক নাম জীবন
৭. কার্বন ডাই অক্সাইড এর একটি উপাদান
৮. একটি প্রাকৃতিক দুযোর্গ
৯. প্রাকৃতিক একটি অবস্থা যেখানে রোদ, বৃষ্টি, ঝড় প্রতিনিয়ত বদলে যাচ্ছে
১০. পুনরায় ব্যবহার উপযোগী
১১. তুফান
১৩. একটি প্রাকৃতিক দুর্যোগ
১৫. আমরা খাবার খেয়ে যা অর্জন করি

পাশাপাশি :

১. সূর্য থেকে আমরা যে শক্তি পাই
২. যা আমাদের সকল সুখের মূল
৫. ভালোভাবে প্রস্তুত থেকে জলবায়ু পরিবর্তনজনিত স্বাস্থ্যহানি প্রতিহত করার ক্ষমতা বৃদ্ধি
১২. পানির একটি উৎস
১৪. বায়ু
১৬. এডিস মশাবাহিত একটি রোগ
১৭. প্রাকৃতিক একটি অবস্থা
১৯. গরম
২০. বন্যা পরবর্তি যে রোগে মৃত্যর হার সবচেয়ে বেশি

কোনাকুনি :

১৮. অ্যানোফিলিস মশা বাহিত রোগ

তোমার উত্তর শিক্ষককে দেখাও এবং সঠিক উত্তরগুলো জেনে নাও। সঠিক উত্তর শিক্ষকদের জন্য প্রণীত সহায়িকাটিতে দেওয়া আছে।

শব্দকোষ

মানুষের দ্বারা জলবায়ু পরিবর্তন

অ্যানথ্রোপোজেনিক মানে 'মানবসৃষ্ট'। জলবায়ু পরিবর্তনের ক্ষেত্রে এটি গ্রিনহাউস গ্যাসকে বোঝায়, অথবা গ্যাস নির্গমন যা দৈনন্দিন কাজ কর্মের জন্য নির্গত হয়। এগুলো হলো শক্তির জন্য জীবাশ্ম জ্বালানি পোড়ানো, গাছ কাটা এবং জমি ব্যবহার পরিবর্তন যা সম্যকভাবে নির্গমন বাড়ায়।

বায়ুপরিমণ্ডল

পৃথিবীর চারপাশে গ্যাসের বিভিন্ন স্তরের আবরণ আছে। শুষ্ক বায়ুমণ্ডল পুরোটাই নাইট্রোজেন এবং অক্সিজেন দিয়ে তৈরি তার সাথে কতগুলো ট্রেস গ্যাস আছে। যেমন আরগন, হিলিয়াম এবং সূর্যরশ্মিযুক্ত, গ্রিনহাউস গ্যাস যথা কার্বন-ডাই-অক্সাইড, মিথেন এবং ওজোন। এর উপরে, বায়ুমণ্ডলে আছে জলীয় বাষ্প, মেঘ এবং এ্যারোসল।

বায়োফুয়েল

সমগ্র জীবের কোষময় পদার্থ (সেলুলোয়িক বায়োমাস) দিয়ে যে জ্বালানি তৈরি হয় তাকে বায়োফুয়েল বলে। বায়োফুয়েলের মধ্যে আছে ইথানল, বায়োডিজেল এবং মিথানল। জীবাশ্ম জ্বালানির পরিবর্তে বায়োফুয়েল উৎপাদনের জন্য জমির ব্যবহার বেশি হলে সে জমিতে শস্য উৎপাদন করা যাবে না। এছাড়া বনাঞ্চল, পিটের জমি, পতিত বা খাস জমি (গ্রাসল্যান্ড) থেকে যদি খাদ্যজনিত বায়োফুয়েল উৎপাদন করা হয় তাহলে ৪২০ গুণ বেশি কার্বন-ডাই-অক্সাইড নিক্ষিপ্ত হয় যা জীবাশ্ম জ্বালানির বদলে বায়োফুয়েলের ব্যবহার করলে বছরে যে গ্রিনহাউস গ্যাস (জিএইচজি) কমবে তার চেয়ে অনেক বেশি।

কার্বন-ডাই-অক্সাইড (CO_2)

স্বাভাবিকভাবে উৎপন্ন গ্যাস যা জীবাশ্ম জ্বালানি পোড়ালে, জমির ব্যবহার করলে এবং অন্যান্য শিল্পে ক্রিয়াকলাপজনিত কারণে উদ্ভূত হয়। এটি একটি প্রধান গ্রিনহাউস গ্যাস যা পৃথিবীর রশ্মিসংক্রান্ত ভারসাম্যকে প্রভাবিত করে এবং এর দ্বারা অন্যান্য গ্রিনহাউস গ্যাস মাপা হয়। মার্চ ২০০৬ সালে কার্বন-ডাই-অক্সাইড মাত্রা ছিল প্রতি ১০ লাখে ২৮১ (পিপিএম) যা প্রাক-শিল্পকরণ গড়ের চেয়ে ১০০ পিপিএম বেশি।

কার্বন ফুটপ্রিন্ট

এটি হলো কোনো বস্তু বানানোর জন্য যে জীবাশ্ম জ্বালানি পোড়ানো হবে তার থেকে কী পরিমাণ কার্বন-ডাই-অক্সাইড বা CO_2 নির্গমন হবে তার পরিমাপ। কার্বন ফুটপ্রিন্টের স্থুল এবং তার সীমা নানারকম হতে পারে এবং এটি যখন সমস্ত জিএইচজিকে অন্তর্ভুক্ত করবে তখন ফুটপ্রিন্টকে CO_2 সমার্থক একক হিসাবে বলা হবে। করপোরেট ক্ষেত্রের কার্যক্ষমতা পরিমাপের জন্য এটি মুখ্য সহায়। কোনো ব্যক্তির কার্বন ফুটপ্রিন্ট তার ব্যক্তিগত ক্রিয়াকলাপের ইঙ্গিত দেয়, যেমন– বাড়িতে বিদ্যুতের ব্যবহার, নিজস্ব যানবাহন এবং কতটা শক্তি খরচ করে তার উপর।

জলবায়ু পরিবর্তন

পরিসংখ্যান অনুযায়ী গুরুত্বপূর্ণ বিভিন্নতা যা জলবায়ুর মধ্যবর্তী অবস্থা বা তার বৈষম্যের মধ্যে, যা দীর্ঘ সময় ধরে টিকে আছে (বিশেষ করে যুগ যুগ ধরে অথবা তারও বেশি)। জলবায়ু পরিবর্তন প্রকৃতির নিজস্ব অন্তর্ভুক্ত প্রক্রিয়ার কারণে হতে পারে, বাইরের চাপে অথবা ক্রমাগত মানুষের ক্রিয়াকর্মের দ্বারা হতে পারে। ইউএনএফসিসি জলবায়ু পরিবর্তন– এর কারণ প্রত্যক্ষ অথবা পরোক্ষভাবে মানুষের কাজ কর্মকেই দায়ী করেছে যা পৃথিবীর বায়ুমণ্ডলের গঠনকে বদলে দিয়েছে এবং অতিরিক্তভাবে তুলনামূলক সময়সীমার মধ্যে প্রাকৃতিক জলবায়ু বৈষম্যের কারণ হচ্ছে।

কার্বন সিঙ্ক

কার্বন সিঙ্ক হচ্ছে এমন একটি স্বাভাবিক প্রক্রিয়া যা বায়ুমণ্ডল থেকে কার্বন-ডাই-অক্সাইড শোষণ করে নেয়। যেমন, গাছ লাগিয়ে এবং বনাঞ্চল রক্ষার মাধ্যমে কার্বন-ডাই-অক্সাইড বনভূমিতে শোষিত হয়- এর মাধ্যমে বনাঞ্চলে এবং বৃক্ষরাজিতে কার্বন জমা থাকে।

গ্রিনহাউস গ্যাস (জিএইচজি)

বায়ুমণ্ডলের যে গ্যাসগুলো সূর্যরশ্মিকে একটি বিশেষ ব্যবধানে গ্রহণ এবং নিক্ষেপ করে। পৃথিবীর সমতল বায়ুমণ্ডল এবং মেঘ যে অবলোহিত রশ্মি বিকিরণ (রেডিয়েশন) করে তা এই গ্যাস গ্রহণ এবং নির্গমন করে। জলীয়বাষ্প, কার্বন-ডাই-অক্সাইড, মিথেন এবং ওজোন হলো বায়ুমণ্ডলের প্রধান গ্রিনহাউস গ্যাস। সমপরিমাণ CO_2 এর তুলনায় মিথেন পৃথিবীকে ২৩ গুণ এবং নাইট্রাস অক্সাইড ২৯৬ গুণ বেশি উষ্ণ করে।

বিশ্বব্যাপী উষ্ণতা বৃদ্ধি

বায়ুমণ্ডলের গড় তাপমাত্রা বেড়ে যাওয়া বিশেষ করে এর ক্রমাগত বৃদ্ধি যা জলবায়ু পরিবর্তনে যথেষ্ট সক্ষম।

ন্যাশনাল অ্যাডাপটেশন প্রোগ্রামস অফ অ্যাকশন

নাপা (NAPA) সর্বাগ্রে করণীয় কাজগুলো চিহ্নিতকরণের একটি পদ্ধতি চালু করে। যা জলবায়ু পরিবর্তনের সময়ে অ্যাডাপ্টেশনের ব্যাপারে জরুরি এবং তাৎক্ষণিক চাহিদা পূরণ করছে। দৃশ্য বিবরণীমূলক মডেলিং এ সাহায্যে ভবিষ্যৎ বিপজ্জনকতা এবং রাষ্ট্রভিত্তিক দীর্ঘস্থায়ী নিয়মনীতি বানানোর বদলে এনএপিএর তৃণমূল স্তরের বর্তমান কৌশলগুলো ব্যবহার করে এবং সর্বাগ্রে করণীয় কাজের জন্য তাকে আরও উন্নত করে। এনএপিএ পদ্ধতিতে জরুরি তথ্য হিসেবে তৃণমূল পর্যায়কে প্রাধান্য দেওয়া হয়, কারণ তৃণমূলে বসবাসরত জনগণ সবচেয়ে বেশি বিপদগ্রস্ত হয়।

ইউএন ফ্রেমওয়ার্ক কনভেনশন অন ক্লাইমেট চেঞ্জ

১৯৯২ সালে ব্রাজিলের ধরিত্রী সম্মেলনে জাতিসংঘের ইউনাইটেড নেশনস কনফারেন্স অন এনভায়রনমেন্ট অ্যাণ্ড ডেভেলপমেন্ট অধিবেশনে এই কনভেনশনটি স্বাক্ষরিত হয়।

যে সরকারগুলো এই চুক্তিতে সম্মত হয়েছে সে সব দেশ বায়ুমণ্ডলে গ্রিনহাউস গ্যাস -এর ঘনত্বের একটি ভারসাম্য আনতে সম্মত হয়েছে যা জলবায়ু পরিবর্তনে মনুষ্যসৃষ্ট্য বিপজ্জনক হস্তক্ষেপ বন্ধ করবে। বিভিন্ন দেশকে জলবায়ু পরিবর্তনের প্রভাব ও বিপদজ্জনকতা বোঝাতে সাহায্য করার জন্য একটি আন্তর্জাতিক কাঠামো তৈরি হয়েছে যেন তারা নিজেদের ক্ষমতা বাড়াতে পারে এবং জ্ঞাত সিদ্ধান্তের দ্বারা সফল ভাবে অভিযোজিত হতে পারে।

কিয়োটো প্রোটকল/ ইউএনএফসিসিসি

ইউএনএফসিসিসি দর্শিত যে জিএইচজি-র লক্ষ্য নির্ধারিত হয়েছে সেই ব্যাপারে আন্তর্জাতিক চুক্তি। ১৯৯৭ সালে স্বাক্ষরিত এই চুক্তি ২০০৫ সালে বলবৎ হয় এবং এই বায়ূমণ্ডলে প্রাকৃতিক এবং মনুষ্যসৃষ্ট যে গ্যাসগুলো অপবিকিরণ বা অবলোহিত রশ্মি শোষণ বা নির্গমন করে তার মাধ্যমে গ্রিণহাউস প্রতিক্রিয়া সৃষ্টি হয়। এই চুক্তি অনুযায়ী উন্নত দেশগুলোতে ১৯৯০ সালের সাপেক্ষে ২০১২ সালের মধ্যে ৫ শতাংশ নির্গমন কমাতে হবে।

কনফারেন্স অব পার্টিস (COP)

জলবায়ু পরিবর্তনের ফলে উদ্ভূত সমস্যা মোকাবেলায় পৃথিবীর ১৯৪টি দেশ একে অপরের স্বার্থরক্ষায় সহযোগিতার হাত বাড়াতে কিয়োটো (CMP) নামে একটি চুক্তি স্বাক্ষর করে অনুমোদন করে। ইউএনএফসিসি-র সদস্য রাষ্ট্রগুলোকে পার্টি বলা হয়। রাষ্ট্রসমূহর অংশগ্রহণে বার্ষিক যে সম্মেলন হয় তাকে বলে কনফারেন্স অব পার্টিস (COP)। কার্যকর কর্মসূচির প্রচারণা ও প্রয়োজনীয় সিদ্ধান্ত গ্রহণের জন্য সদস্য রাষ্ট্রগুলোর মধ্যে থেকে একটি উচ্চ পর্যায়ের দল গঠিত হয়েছে। ইউনাইটেড ন্যাশনস ফ্রেমওয়ার্ক কনভেনশন অন ক্লাইমেট চেঞ্জ (UNFCC) এর ১৬ তম কনফারেন্স অফ পার্টিস (COP-16) -এর সদস্য রাষ্ট্রগুলোর প্রতিনিধিগণ ২০১০ সালে মেক্সিকোর কানকুনে সমবেত হয়। এ সম্মেলন চলাকালে বেশিরভাগ শক্তির উৎসই ছিল নবায়নযোগ্য শক্তি। সম্মেলনে জলবায়ু পরিবর্তন জনিত বর্তমান ও আসন্ন সমস্যাগুলোর সমাধানে এক যোগে কাজ করার সিদ্ধান্ত গৃহীত হয়।

কপ-১৭ অনুষ্ঠিত হবে দক্ষিণ আফ্রিকার ডারবানে, ২০১১ সালে। আর ধরিত্রী সম্মেলনের ২০ বছর পূর্তিতে ২০১২ সালে ব্রাজিলের রিও-ডি জেনেরোতে অনুষ্ঠিত হবে কপ-১৮, রিও ২০+ সম্মেলন।

ভালনারেবিলিটি

এটি এমন একটি বিপদসীমা যে সীমা পর্যন্ত কোনো ব্যবস্থা জলবায়ু পরিবর্তনের বৈরি প্রভাব, এর বিপদজ্জনকতা ও চরম অবস্থার প্রতি সংবেদনশীল বা সামঞ্জস্য রাখতে অপারগ। ভালনারেবিলিটি জলবায়ু তারতম্যের বৈশিষ্ট্য, বিস্তার ও ভিন্নতার হার এবং যে পদ্ধতিটি বা ব্যবস্থাটি এই জলবায়ু তারতম্যের সম্মুখীন তার সংবেদনশীলতা এবং সংযোজনের ক্ষমতার উপর নির্ভরশীল।

জলবায়ু পরিবর্তনজনিত জরুরী অবস্থার
First Aid ব্যাগ প্রস্তুতি

ক্রমিক	বিবরণ	পরিমাণ
১.	কাঁধে ঝুলানো ব্যাগ	১টি
২.	ব্যান্ডেজ রোল (২ ইঞ্চি x ৬ গজ)	রোল ১২টি
৩.	ব্যান্ডেজ রোল (৪ ইঞ্চি x ৬ গজ)	রোল ১২টি
৪.	ক্রেপ রোল (৪ ইঞ্চি x ৬ গজ)	২টি
৫.	তুলা (২০০ গ্রাম/রোল)	১টি
৬.	লিউকোপ্লাস্ট (১ ইঞ্চি x ১৫৭ ইঞ্চি)	২ রোল
৭.	লিউকোপ্লাস্ট (২ ইঞ্চি x ১৫৭ ইঞ্চি)	১ রোল
৮.	ড্রেসিং ফোরসেপ (৬ ইঞ্চি)	১টি
৯.	কাঁচি (৫ ইঞ্চি)	১টি
১০.	ছুরি (ছোট)	২টি
১১.	রাবার গ্লাভস্	২ জোড়া
১২.	থার্মোমিটার (ফারেনহাইট)	১টি
১৩.	আই শিল্ড	৩টি
১৪.	প্লাস্টিকের বাক্স ও সাবান	১টি
১৫.	টাওয়েল (মাঝারি)	২টি
১৬.	প্লাস্টিকের বাটি (৮ ইঞ্চি)	১টি
১৭.	ত্রিকোণী কাপড় (৬০ ইঞ্চি)	৫টি
১৮.	টুর্নিকেট	৫টি
১৯.	কাঠের স্প্লিন্টস্ (৬/৮/১০ ইঞ্চি) তিন রকমের	৯টি
২০.	এন্টিবায়োটিক ক্রিম (টেট্রাসাইক্লিন ৩%)	৫ টিউব
২১.	এন্টিসেপ্টিক তরল (ডেটল/স্যাভলন)	১০০ মিঃ গ্রাঃ
২২.	খাবার স্যালাইন (ওআরএস)	২০ প্যাকেট
২৩.	প্যারাসিটামল ট্যাবলেট	৫০টি
২৪.	নেবানল পাউডার (৫ গ্রাম)	৫টি
২৫.	সেফটিপিন (ছোট ও বড়)	১২টি
২৬.	পেপার প্যাড ও পেনসিল	১ সেট
২৭.	টর্চ লাইট (২ ব্যাটারি)	১টি
২৮.	গ্যাস লাইটার	১টি
২৯.	খাবার পানির বোতল (১ লিটার)	৫টি
৩০.	টিস্যু পেপার	১ রোল
৩১	ক্যাপসুল (এ্যামোক্সাসিলিন)	৫০টি

CCHPU

Climate Change & Health Promotion Unit

যোগাযোগের জন্য :

ক্লাইমেট চেঞ্জ অ্যাণ্ড হেলথ প্রমোশন ইউনিট (সিসিএইচপিইউ)

স্বাস্থ্য ও পরিবার কল্যাণ মন্ত্রণালয়

আনসারি ভবন (পঞ্চম তলা)

১৪/২, তোপখানা রোড, ঢাকা - ১০০০

টেলিফোন : +৮৮-০২-৯৫১৩৯৪২

ফ্যাক্স : +৮৮-০২-৯৫১৩৯৪১

ই-মেইল : info@cchpu-mohfw.gov.bd

ওয়েব সাইট : www.cchpu-mohfw.gov.bd
