# Supplementary material for: Child Centred Approach to Climate Change and Health Adaptation through Schools in Bangladesh: A Cluster Randomised Intervention Trial
Source: PLoS One. 2015 Aug 7;10(8):e0134993. doi: 10.1371/journal.pone.0134993 (PMC4529232; doi:10.1371/journal.pone.0134993)
Supplement: S1 Leaflet — (DOC) [file pone.0134993.s006.doc]

Leaflet for the Control School

In our everyday life we say either today is hot or today is cold, the humidity is more or it's windy. Actually we do speak of weather by that. We live in an atmosphere. The daily temperature, wind condition, humidity etc comprises the weather. The surroundings where do we live in comprise the eco-system. Environment is naturally the eco-system of our surroundings.

The soil, water, air are the natural components of our environment and eco-system.

We live in an ecological condition where the weather influences our environment. The average weather condition of last 30 years constitutes the climate. In our planet Earth we have animal and plant kingdom. There is a natural ecological balance between this two.

In our daily life we the human being inhale oxygen and expel carbon-di-oxide through our respiratory system. The plant kingdom does the reverse, they inhale carbon-di-oxide and expel oxygen, and thus the ecological balance of life is maintained in an ideal natural condition.

When the weather condition changes for a longer span of time the climate is influenced over the years. This is called climate change. Our Climate is changing continuously. Sometimes it's natural and sometimes it's man maid. With the modernization of civilization our climate has been changed rapidly. Population is growing, energy use has been increased and deforestation resulted imbalance in the eco-system.So the climate is changing.

Climate includes patterns of temperature, precipitation, humidity, wind and seasons. "Climate change" affects more than just a change in the weather, it refers to seasonal changes over a long period of time. These climate patterns play a fundamental role in shaping natural ecosystems, and the human economies and cultures that depend on them.

Because so many systems are tied to climate, a change in climate can affect many related aspects of where and how people, plants and animals live, such as food production, availability and use of water, and health risks.

For example, a change in the usual timing of rains or temperatures can affect when plants bloom and set fruit, when insects hatch or when streams are their fullest. This can affect historically synchronized pollination of crops, food for migrating birds, spawning of fish, water supplies for drinking and irrigation, forest health, and more.

There is now widespread agreement that current trends in energy use development and population growth will lead to continuing – and more severe – climate change. The changing climate will inevitably affect the basic requirements for maintaining health: clean air and water, sanitary environments, sufficient food and adequate shelter. Many diseases and health problems may be exacerbated by climate change.

The average temperature of the world is increasing day by day. This is called global worming now days. It is predicted that if the current trend continues the earth will be much more warmer in this century and it will be beyond the tolerance level for human being.

There is new and stronger evidence that most of the warming over the last 50 years is due to human activities. Ice cores taken from deep in ancient ice of Antarctica show that carbon dioxide levels are higher now than at any time in the past 650,000 years. More carbon dioxide in the atmosphere means warming temperatures. In 2007 report to the United Nations, the Intergovernmental Panel on Climate Change (IPCC) concluded that it is more than 90 percent likely that the accelerated warming of the past 50-60 years is due to human contributions.

In the solar system in our everyday life we are emitting some gases in the atmosphere. These contributions include increased levels of “heat-trapping” gases (a.k.a. “greenhouse gases”) such as carbon dioxide in the Earth’s atmosphere. One of the biggest ways people contribute to greenhouse gases is by burning fossil fuels. We use coal, oil, and natural gas to generate electricity, heat our homes, power our factories, and run our cars.

Changing land use patterns contribute, too. Trees and other plants use carbon dioxide and give off oxygen. When trees are cut down for development, agriculture, and other purposes, they’re no longer available to take carbon dioxide out of the air, and actually release carbon dioxide as they decay or burn.

As the levels of carbon dioxide and other greenhouse gases increase, more heat is “trapped” and global temperatures rise. Although many “greenhouse gases” occur naturally, human activities have increased their levels and added new ones. The ozone hole is a thinning of the stratosphere's ozone layer, which is roughly 9 to 31 miles above the earth's surface. The depletion of this ozone layer is due to man-made chemicals like chlorofluorocarbons (CFCs). A thinner ozone layer lets more harmful ultraviolet (UV) radiation reach the earth's surface.

Weather and climate have affected human health for millennia. The direct effects of extreme weather events include drowning from floods, injuries from floods and other extreme events. Heat exposure has a range of health effects, from mild heat rashes to deadly heat stroke. Climate change is a complex phenomenon and a range of unanticipated ecological effects may result. Many of these ecosystem effects could have indirect health effects.

Many of the major killers such as diarrhoeal diseases, malnutrition, malaria and dengue are highly climate-sensitive and are expected to worsen as the climate changes. The overall health effects of a changing climate are likely to be overwhelmingly negative. Climate change affects the fundamental requirements for health – clean air, safe drinking water, sufficient food and secure shelter.
